# Supplementary material for: Systematic assessment of long-read RNA-seq methods for transcript identification and quantification
Source: Nat Methods. 2024 Jun 7;21(7):1349–63. doi: 10.1038/s41592-024-02298-3 (PMC11543605; doi:10.1038/s41592-024-02298-3)
Supplement: Supplementary file 1 — Supplementary Results, Discussion, Methods, Tables 1–13 and Figs. 1–79. [file 41592_2024_2298_MOESM1_ESM.pdf]

nature methods

Supplementary information https://doi.org/10.1038/s41592-024-02298-3

Systematic assessment of long-read RNA-seq methods for transcript identification and quantification

In the format provided by the authors and unedited

LRGASP Supplementary Information

This document contains supplementary information, figures, and tables supporting *Systematic assessment of long-read RNA-seq methods for transcript identification and quantification*.

Supplementary Results 2
Quality of LRGASP data 2
Challenge 1 Results and Evaluation: Transcript isoform detection with a high-quality genome 2
Experimental Validation of Transcript Isoform Predictions 5
Supplementary Discussion 5
Supplementary Methods 6
LRGASP Challenge Details 6
Submissions and Timeline 6
Requirements for Challenge 1 and 2 7
Requirements for Challenge 3 9
Capping SIRVs 10
Mouse and human RNA sample preparation 10
Manatee RNA sample preparation 10
Manatee genome sample preparation 11
cDNA preparation for Illumina and PacBio sequencing of human and mouse 12
PacBio library preparation of human and mouse libraries 12
CapTrap preparation for PacBio and ONT sequencing of human and mouse 13
R2C2 preparation for ONT sequencing of human and mouse 14
cDNA preparation for ONT sequencing of human and mouse 15
Direct RNA (dRNA) preparation for ONT sequencing of human and mouse 15
Manatee ONT genome sequencing and assembly 16
Manatee cDNA PacBio library preparation and sequencing 16
Manatee cDNA Nanopore library preparation and sequencing 18
Long-read data processing 18
Reference genome and annotations 19
Simulated data 19
CAGE data of WTC11 samples for validation of transcript 5’ ends 20
LRGASP Data QC 21
GENCODE Benchmarks and Computational Evaluation 22
Primers-JuJu bulk RT_PCR primer design tool 23
Computational Pipeline Description from Submitters 25
Challenge 1 25

Challenge 2 32
Challenge 3 37
Method changes from registered report phase 1 38
Supplementary Tables 39
Supplementary Table 1: Overview of LRGASP sequencing data 39
Supplementary Table 2: Summary statistics for ES sequencing data 39
Supplementary Table 3: Error rates in percentage for real and simulated data of different types obtained via read alignment 40
Supplementary Table 4: Summary statistics for WTC11 sequencing data 41
Supplementary Table 5: Summary statistics for H1-mix sequencing data 41
Supplementary Table 6: Summary statistics for Manatee sequencing data 42
Supplementary Table 7: LRGASP participation summary 43
Supplementary Table 8: Metrics for evaluation against GENCODE annotation 44
Supplementary Table 9: Metrics and definitions for evaluation against SIRVs 45
Supplementary Table 10: Metrics and definitions for evaluation against simulated data 46
Supplementary Table 11: Metrics for evaluation of manually annotated transcript models 47
Supplementary Table 12: WTC-11 Validation Batches 47
Supplementary Table 13: Method Changes 48
References 50
Supplementary Figures 51

Supplementary Results

Quality of LRGASP data

The quality of the simulated data was verified by mapping both real and simulated reads to their respective genomes using minimap2[5] in spliced mode, and empirical error rates were computed (**Supplementary Table 3**). As indicated by the table, error rates were found to be similar across the board, with the exception of cDNA-ONT data. For cDNA-ONT data, it was observed that real data sequenced in this study exhibited greater accuracy compared to reads generated by NanoSim[1].

Challenge 1 Results and Evaluation: Transcript isoform detection with a high-quality genome

When looking at predictions using datasets of real samples, we noticed that the fraction of the long reads that were used to build the transcript model (Percentage of Reads Used, PRU) greatly varied among and within

methods, with tools such as LyRiC13 only utilizing less than 20% of the available reads and others, e.g., Iso_IB[2], FLAMES[3], and IsoQuant[4] showing PRU greater than 100%, indicating that the same read supported more than one transcript model (Supplementary **Fig. 52**), and revealing very different strategies in the reconstruction of the transcriptomes.

In general, pipelines detected more genes using cDNA library preparations and the PacBio sequencing platform. At the same time, there was not a clear best library preparation method for ONT in terms of the number of detected genes (Supplementary **Fig. 53**) or transcripts (Supplementary **Fig. 54)**. The same trend was observed when comparing results within each analysis tool **(Supplementary Figs. 55-58**).

To understand which characteristics were driving differences in the detection of transcripts by the long-read technologies, we compared expression level, transcript length, and the number of exons of transcripts exclusively detected by each experimental method. We found that transcripts found only by pipelines processing PacBio reads were longer and had more exons and lower expression values than transcripts detected by Nanopore only or by both technologies (Supplementary **Fig. 59**). This pattern was also seen in the transcripts exclusively identified when tools analyzed cDNA data –and to a lesser extent R2C2-ONT, compared to those only detected by other library preparations methods (Supplementary **Fig. 60**). The combination cDNA-PacBio was the experimental procedure where their exclusively detected transcripts were the longest and had significantly lower expression (Supplementary **Fig. 61**). These results reveal that global capacity for transcript detection is associated with quality parameters of the sequencing reads. Interestingly, although these differences in transcript length were broadly recapitulated by analysis method, large transcripts (>10,000 bases in length) were exclusively reported by Bambu, IsoQuant, StringTie2, and TALON-LAPA (Supplementary **Fig. 62**).

Upon comparing 47 analysis pipelines, it was observed that the majority of transcripts were consistently detected by only a few pipelines. This prompted us to ask whether such inconsistency stemmed from differences in transcript capture by the diverse experimental protocols or because of the analytical approaches employed. To address this question, we assessed the consistency among tools for the detection of known and novel Unique Intron Chains (UICs) within datasets generated from each unique combination of library preparation methods and sequencing platforms. We found that FSM was the transcript type most reliably identified across tools, with tens of thousands of distinct UICs being detected in datasets from cDNA-PacBio and R2C2-ONT, whereas consistent detection of ISM, NIC, and NNC in these protocols was only in the range of hundreds. Other experimental combinations, such as CapTrap-PacBio, CapTrap-ONT, and direct RNA-ONT, yielded concordance rates significantly lower, by order of magnitude, with FSM detections in the thousands and ISM and NNC in the tens.

Even though every individual tool detected other structural categories like Fusion, Antisense, Genic Genomic, and Genic Intron, these types of transcripts vanished when replicability across analysis methods was enforced. Further examination of UIC detection consistency by the same tool across various experimental datasets yielded similar findings: FSM transcripts showed the highest consistent detection rates across experimental protocols, whereas ISM, NIC, and NNC counts significantly declined as more protocols were included, with just a few hundred transcript models being identified by more than three datasets. Mandalorion was a notable exception, demonstrating consistent UIC detection numbers across different experimental conditions, including in the ISM, NIC, and NNC categories. Multiple experimental protocols failed to consistently detect UICs within other SQANTI categories (Supplementary **Fig. 63**). LyRic exhibited the lowest count of consistently detected transcript models.

Subsequently, a set of "frequently detected transcripts" (FDT) was defined, comprising UICs identified in at least three experimental datasets and by a minimum of three analysis tools. This collection included approximately 45,000 UICs per biological sample, accounting for roughly 8% of the total UICs detected by any method. Within this set, the vast majority (~80%) were FSMs, with NIC transcripts representing about 10% of the FDT (Supplementary **Fig. 64)**. This indicates that FSMs were enriched four to fivefold in the FDT set compared to their overall detection rate, whereas other categories significantly diminished in frequency. The FDT set was most commonly identified by IsoQuant, Mandalorion, Bambu, TALON-LAPA, and FLAMES over other tools, even after adjusting for the number of analysis pipelines each laboratory contributed (Supplementary **Fig. 64**).

We observe discrepancies among performance results depending on the benchmark. For example, LyRic performed poorly on SIRVS and GENCODE manual annotation but well on simulated data. In general, sensitivity for novel transcripts when using simulated data resulted in better performance than when using the GENCODE manual annotation, indicating challenges in benchmarking approaches. It could be argued that the GENCODE manual annotation is based on real data that presents a more realistic annotation challenge; however, the manual review resulted in a bias in locus selection that had relatively fewer reads to review with fewer replicates in different libraries.

Our analysis of manually annotated transcripts revealed that many new isoforms were identified exclusively within a single dataset. Consequently, we refined our performance metrics to include only those transcripts observed in at least two experimental samples (totaling 114 transcripts) or those supported by more than two reads (94 transcripts). Implementing these criteria did not significantly impact the sensitivity of the methods but did reduce the precision of novel transcript detection (Supplementary **Figs. 65-66)**. Upon reassessing

performance metrics using the library preparation method and sequencing platform, we found no definitive superior experimental approach. Nonetheless, there was a marginally higher precision in detecting novel transcripts and greater sensitivity for identifying known transcripts when using tools tailored for PacBio datasets, as opposed to those designed for Nanopore datasets (Supplementary **Figs. 67-68**).

Experimental Validation of Transcript Isoform Predictions

The analysis of genomic features, such as transcript length, revealed no apparent correlation with validation rates (Supplementary **Fig. 69**). Although long-read transcript alignments frequently succeed in clarifying regions that pose challenges for the more precise short-read alignments, these findings underscore the existence of "blind spots" in long-read-based transcript annotation. Such blind spots include imperfectly aligned reads—for example, tandem repeats, inversions, and short exon overhangs—where the accuracy of isoform alignment can be significantly influenced by the assumptions made by the alignment software. While expert human intervention can address many of these challenging cases, some remain irresolvable.

Supplementary Discussion

The novel transcript class with the highest overall consensus and validation was the NIC, implying that novel combinations of splice junctions, TTS, and TSS in the transcriptome are to be expected, at least for well-characterized organisms like mouse and human. Surprisingly, ~50% of the tested NNCs were validated, indicating additional novelty and high false discovery in this category. These results point to the utility of having a deep reference annotation capturing the largest possible catalog of splice features. Interestingly, many of these novel transcripts were detected by just one or few reads, found in only one or few samples, yet still validated by PCR. This suggests that many rare RNA molecules are present in specific samples, raising questions about considering them when reporting transcriptome composition using long reads. Arguably, while the comprehensive profiling of the RNA molecular content of a particular sample may require the inclusion of any detected transcript when defining the transcriptional signature of cell types and cell states, including only consistently detected transcripts is advisable. The LRGASP results show that the analysis strategies implemented by the different tools are differently suited for these two scenarios.

Supplementary Methods

LRGASP Challenge Details

Submissions and Timeline

Participants submitted challenge predictions to Synapse (https://www.synapse.org/).

The following is an overview of the data used for each challenge and the result files that were submitted (Supplementary **Figs. 70-73**).

- Challenge 1: transcript isoform detection with a high-quality genome (iso_detect_ref)
    - Samples
        - WTC11 (human iPSC cell line)
        - H1-mix (human H1 ES cell line mixed with human Definitive Endoderm derived from H1)
        - ES (mouse ES cell line)
        - human simulation - simulated human reads (Illumina,cDNA-ONT, and cDNA-PacBio)
        - mouse simulation - simulated mouse reads (Illumina and cDNA-PacBio, dRNA-ONT)
    - Result files:
        - models.gtf.gz
        - read_model_map.tsv.gz
- Challenge 2: transcript isoform quantification (iso_quant)
    - Samples
        - WTC11 (human iPSC cell line)
        - H1-mix (human H1 ES cell line mixed with human Definitive Endoderm derived from H1)
        - Human simulation - simulated human reads (Illumina, cDNA-ONT, and cDNA-PacBio)
        - Mouse simulation - simulated mouse reads (Illumina and cDNA-PacBio, dRNA-ONT)
    - Result files:
        - expression.tsv.gz
        - models.gtf.gz
- Challenge 3: de novo transcript isoform detection (iso_detect_de_novo)
    - Samples
        - Manatee (manatee whole blood)

        - ES (mouse ES cell line)
    - Result files:
        - rna.fasta.gz
        - read_model_map.tsv.gz

Computational methods may have been developed and tuned to a specific sequencing platform, library prep approach (e.g., dRNA-ONT), or use of additional orthogonal data; therefore, entries were organized such that a comparison can be made across different tools using the same type of data. Additionally, it was important to evaluate how robust computational tools are to transcript analysis in different species or biological samples. Thus, for each entry to a challenge, a team selected a data category, library prep, and sequencing platform and submitted experiments for all samples that are available for the challenge + library prep + sequencing platform combination (Supplementary **Fig. 70)**. The samples that are available for a challenge + library prep + sequencing platform combination can be found in Supplementary Table **1**. Note that there are also simulated samples that were also available for Challenges 1 and 2.

Each entry must have met the following requirements:

Requirements for Challenge 1 and 2

At least one experiment must have been supplied for each sample available for a given challenge, library prep, and sequencing platform combination that is selected. Human and mouse samples have biological replicates that must have been used for the entry.
A major goal of LRGASP is to assess the capabilities of long-read sequencing for transcriptome analysis and how much improvement there is over short-read methods. Additionally, long-read computational pipelines vary in their use of only long-read data or if they incorporate additional data for transcript analysis. To facilitate comparisons between long-read and short-read methods and variation in tool parameters, we broke down submissions into different categories:

- long-only - Use only LGRASP-provided long-read RNA-seq data from a single sample, library preparation method, and sequencing platform.
- short-only - Use only LGRASP-provided short-read Illumina RNA-seq data from a single sample. This is to compare with long-read approaches.

- long and short - Use only LGRASP-provided long-read and short-read RNA-Seq data from a single long-read library preparation method and the Illumina platform. Additional accessioned data in public genomics data repositories can also be used.
- freestyle - Any combination of at least one LRGASP data set as well as any other accessioned data in public genomics data repositories. For example, multiple library methods can be combined (e.g., cDNA-PacBio + CapTrap-PacBio, cDNA-ONT + CapTrap-ONT + R2C2-ONT + dRNA-ONT, all data, etc.).

In all the above categories, the genome and transcriptome references specified by LRGASP were used. For the long, short, and freestyle categories, additional transcriptome references could be used.

All replicates must have been used in each experiment. Challenge 2 must have reported replicates separately in the expression matrix. Each team could submit multiple entries for each challenge; however, they can only submit one entry per challenge + data type + library prep + sequencing platform combination. This was to encourage tool development that is robust to different library preps and sequencing platforms but prevents multiple entries that are subtle parameter changes.

For Challenge 1, the submitted GTF file only contained transcripts that had been assigned a read. For Challenge 2, submitters had the option of quantifying against the reference transcriptome or a transcriptome derived from the data (i.e., results from Challenge 1). The GTF used for quantification was included as part of the Challenge 2 submission.

The type of platform and library preparation method used in a given experiment, except for freestyle experiments, was limited to data from a single library preparation method plus sequencing technology (long-only). LRGASP Illumina short-read data of the same sample could optionally be used in an experiment with the LRGASP long-read data (long and short):

- cDNA-Illumina - short-only
- cDNA-PacBio - long-only or long and short
- CapTrap-PacBio - long-only or long and short
- cDNA-ONT - long-only or long and short
- CapTrap-ONT - long-only or long and short
- R2C2-ONT - long-only or long and short
- dRNA-ONT - long-only or long and short

Requirements for Challenge 3

At least one experiment had to be supplied for each sample available for a given library prep and sequencing platform combination that was selected. Mouse samples had biological replicates that were used for the entry. Manatee samples only had cDNA library preparation type and sequencing data from Illumina, ONT, and PacBio.

For similar reasons as described above, the data used for a given experiment had to fit into one of the following categories:

- long-only - Use only LGRASP-provided long-read RNA-Seq data from a single sample, library preparation method, and sequencing platform. No genome reference can be used.
- short-only - Use only LGRASP-provided short-read Illumina RNA-Seq data from a single sample. This is to compare with long-read approaches. No genome reference can be used.
- long and short - Use only LGRASP-provided long-read and short-read RNA-Seq data from a single long-read library preparation method and the Illumina platform. No genome reference can be used.
- long and genome - Use only LGRASP-provided long-read RNA-Seq data from a single long-read library preparation method. A genome reference sequence can be used.
- freestyle - Any combination of at least one LRGASP data set as well as any other accessioned data in public genomics data repositories. For example, multiple library methods can be combined (e.g., cDNA-PacBio + CapTrap-PacBio, cDNA-ONT + CapTrap-ONT + R2C2-ONT + dRNA-ONT, all data, etc.).

In all the above categories, except for freestyle, a transcriptome reference could not be used. The submitted FASTA file only contained transcripts that had been assigned a read. Each team could submit multiple entries for each challenge; however, they could only submit one entry per challenge + data type + library prep + sequencing platform combination.

LRGASP biological data was available at the ENCODE DCC. The simulated data was made available via Synapse. The competition was launched on May 1, 2021, and challenge submissions were closed on October 8, 2021. Figures giving a summarized overview of the challenges, including specific samples used and expected entry files (Supplementary **Figs. 70,74)**, challenge evaluations (**Supplementary Figs. 75-77**), and experimental validation (Supplementary **Fig. 78)**, are provided in the **Supplementary Figures** section below.zz

Additional details of all protocols for library preparation and sequencing can be found at the ENCODE DCC and are linked to each dataset produced by LRGASP (**Supplementary Data 1**).

Capping SIRVs

Exogenous synthetic RNA references (spike-ins) are widely used to calibrate measurements in RNA assays, but they lack the 7-Methylguanosine (m^7G) cap structure that most natural eukaryotic RNA transcripts bear at their 5' end. This characteristic makes commercial spike-in mixes unsuitable for library preparation protocols involving 5' cap enrichment steps. Therefore, we enzymatically added the appropriate m^7G structure to the SIRV standards used in this challenge following the CapTrap protocol[5]. Specifically, the pp5'N structure present at the 5' end of the spike-in sequence was used as a template for the Vaccinia capping enzyme (catalog num M2080S, New England BioLabs) to add the m^7G structure to SIRV-Set 4 (Iso Mix E0 / ERCC / Long SIRVs, catalog num 141.03, Lexogen). A total of ten vials of SIRV-Set 4 (100 µl) were employed to perform the capping reaction (final total mass of 535 ng). The reaction was performed following the recommendations of the manufacturer's capping protocol with two minor changes: 3.5 µl of RNAse inhibitors (RNasin Plus RNase Inhibitor, catalog num N2611, Promega) were added to the capping reaction to avoid RNAse degradation, and the incubation time was extended from 30 minutes to two hours, following a recommendation from New England BioLabs technical support scientists. The final capping reaction was purified by using 1.8x AMPure RNA Clean XP beads (catalog num. A63987, Beckman Coulter) and resuspended in 100 µl of nuclease-free water.

Mouse and human RNA sample preparation

Prior to the distribution of the biosample total RNA aliquots to each of the participating labs, 110 µg of each biosample total RNA was spiked with Lexogen Long SIRV Set-4 quantification standards (catalog # 141.03) at approximately 3% of the estimated mRNA mass present (~1% of total RNA). The mass of capped SIRVs used was 29.5 ng, and the mass of uncapped SIRVS used was 28.9 ng. In the case of direct RNA sequencing of one replicate of WTC11 (ENCODE library accession ENCLB926JPE) and one replicate of mouse ES cells (ENCODE library accession ENCLB386NNT), only uncapped SIRV 4.0 were spiked in at approximately 3% of the estimated mass. Appropriate volumes of the spiked total RNA mixture to meet the input mass requirements for each library preparation method were then aliquoted separately, stored at -80 C, and shipped on dry ice to participating labs.

Manatee RNA sample preparation

Blood samples from Florida manatees were collected during health assessments by the U.S Geological Survey (USGS) Sirenia Project, the Florida Fish and Wildlife Conservation Commission (FWC), and the University of

Florida under U.S. Fish and Wildlife Service (USFWS) permit # MA791721-5 in Crystal River (Citrus County, Florida, USA) and in Satellite Beach (Brevard County, Florida, USA) in December and January of 2018 and 2019 respectively. Samples were processed under the University of Florida USFWS permit #MA067116-2 following a protocol approved by the ethics committee (IACUC # 201609674 & IACUC # 201909674). Whole blood from minimally restrained Florida manatees was collected from the medial interosseous space between the ulna and radio from the pectoral flippers. Samples were drawn using Sodium Heparin 10-mL BD vacutainers (BD BioScience, New Jersey, U.S.A). Blood samples were spun on-site, and the plasma was aliquoted, stored in liquid nitrogen or ice, and transferred to -80 ºC once in the lab. The buffy coat (white blood cells) was flash-frozen in liquid nitrogen on-site, and total RNA was subsequently extracted in the lab using STAT 60 (Tel-test Friendswood, TX) reagent. Approximately 350 µL of the frozen buffy coat was added to 1 ml of STAT 60 and vortexed for 30 seconds, 250 µL of chloroform was added, and the tube was centrifuged 20,800 x g for 15 minutes at 4 ºC to extract the RNA. This step was repeated, and then RNA was precipitated from the supernatants overnight at -20ºC by the addition of 700 µL isopropanol with 1.5 µL of GlycoBlueTM (15 mg/mL) (Ambion, Invitrogen, Austin, TX) as a coprecipitant. Following centrifugation at 20,800 x g for 45 minutes, the pellet was washed with ethanol 70%, air-dried, and resuspended in 20 mL of RNA secure (Ambion, Austin, TX). A DNAse treatment was performed using a Turbo DNA-freeTM kit (Ambion, Austin, TX). A total of nine good-quality RNA samples were selected to create an RNA pool. These samples included six females, one calf, one lactating female, and one male, and had RIN values from 8.0 to 8.8.

Manatee genome sample preparation

The genome of the Florida manatee Lorelei was sequenced using Nanopore and PacBio. Lorelei is the same individual manatee for which an Illumina-based genome assembly was released by the Broad Institute in 2012 [6]. An EDTA -80ºC whole blood sample aliquot was used. gDNA was extracted from 1400 µl of blood using the DNeasy kit (QIAGEN, MD, USA) following the companies' specifications for 100 µl aliquots of blood. Thawed blood was diluted 1:1 with RNA free Phosphate buffered saline 1x (Gibco, UK), 20 µl of proteinase K (QIAGEN, MD, USA), and 200 ul of AL lysis buffer (QIAGEN, MD, USA) and vortexed immediately. It was incubated at 56 °C for 10 minutes. Then, we added 200 µl of ethanol 96% and mixed it thoroughly. The mixture was added to the DNeasy mini spin-column and centrifuged at 6,000 x g for 1 minute. The column was washed with 500 µl of AW1 solution (QIAGEN, MD, USA) and centrifuged at 6,000 x g for 1 minute, followed by a wash with 500 µl AW2 (QIAGEN, MD, USA) and centrifuged at 20,000 x g for 3 minutes. gDNA was eluted twice with 100 µl of AE buffer added to the center of the column, incubated for 1 minute, and centrifuged 6,000 x g for 1 minute. The first and second elutions from the DNeasy mini spin-column were pooled and concentrated using a speed vacuum for 20 minutes, in which each preparation was reduced from 200 to 50 µl.

All gDNA tubes were pooled, and the DNA was cleaned with AM Pure magnetic beads (Beckman Coulter-Life Sciences, IN, USA) at a ratio of 0.5:1, beads volume to gDNA volume (50 µl of beads to 100 µl of gDNA). gDNA bound to the beads was washed twice with 1 ml of 70% ethanol. Ethanol traces were removed by quick spin to the bottom of the tube and removed with a pipette. Then, the beads were dried for 2 minutes, and gDNA was eluded in 55 µl of EB buffer (QIAGEN, MD, USA) at 37 °C with 10 minutes of incubation. This process was repeated twice. Quantification of gDNA was performed with a Qubit™ fluorometer (Thermo Fisher Scientific), and the quality of the gDNA was assessed using a Genomic Tape on the Agilent TapeStation (Santa Clara, CA, USA). The final DNA quantity was 28.8 µg of DNA at a concentration of 267 ng/µl. The DNA Integrity Number (DIN) was 8.8, and the peak size was 54.5 kb.

cDNA preparation for Illumina and PacBio sequencing of human and mouse

PacBio cDNA synthesis was performed using a modified version of the Picelli protocol[7] substituting the Maxima H- reverse transcriptase. Total RNA (400 ngs) spiked with SIRV standards was combined in a priming reaction with RNAse inhibitor, oligo dT, dNTPs, and water, incubated at 72ºC for 3 minutes, then ramped down to 50ºC for an additional 3 minutes. We then added a first-strand synthesis buffer (5x RT buffer, TSO oligo, Maxima H(-) RT, and water) that had previously been equilibrated to 50ºC to the priming reaction. First-strand synthesis was carried out as follows: (Extension at 50ºC for 90 min, 85ºC for 5 min, and held at 4ºC). To the first strand reaction, we then added 2x SeqAmp (Takara) reaction buffer, IS primers, water, and SeqAmp polymerase). First-strand cDNA was amplified for 11 cycles as follows: (95ºC 1 min, 98ºC 15 sec, 65ºC 30 sec, and 68ºC 13 min), and finished off by incubation at 72ºC for 10 min and holding at 4ºC. The amplified products were purified using SPRI beads, quantified on Qubit, and checked for length distribution on the Agilent Bioanalyzer. The short-read protocol is described in the *Nextera DNA Flex Library Prep Reference Guide*[8,] and the long-read protocol is in *Long read cDNA prep with Maxima H(-) (no exonuclease version)*[9]. 50 ng sub-aliquots of the full-length cDNA libraries were tagmented for Illumina short-read sequencing using the Illumina Nextera DNA Flex Library prep kit, according to the manufacturer's protocol.

PacBio library preparation of human and mouse libraries

To build PacBio libraries, we followed the SMRTbellTM Express Template Prep Kit 2.0 protocol. We started from 500 ng of poly(A) selected cDNA. The ends of the cDNA were repaired first in order for the cDNA molecule to be suitable for the ligation of SMRTbell adapters. We added a damage repair reaction (DNA prep buffer, NAD, and DNA damage repair) and then incubated at 37ºC for 30 min. Then End prep mix was added and incubated at 20ºC for 30 min and 65ºC for 20 min. Ligation of the adapter at the ends of the cDNA was done by adding a ligation mix (PacBio adapters, ligation mix, ligation enhancer, and ligation additive), followed

by incubation at 20ºC for 60 min. Final libraries were cleaned up using SPRI beads, and we recorded the size and concentration of samples. Once the ligation step was done and the libraries passed the QC, a sequencing primer was annealed to the adapters in the UCI GHTF sequencing facility to allow for the binding of the polymerase during sequencing.

CapTrap preparation for PacBio and ONT sequencing of human and mouse

CapTrap is a technique developed by the Guigó laboratory (CRG, Barcelona, Spain) in collaboration with Piero Carninci's group in RIKEN, Japan. The method enriches for full-length transcripts by selection of the 7-Methylguanosine (m7G) cap structure present at the 5' ends of RNA transcripts, followed by specific cap- and poly(A)- dependent linker ligations. The cDNA libraries generated using this method are compatible with long-read sequencing platforms (ONT or PacBio). The protocol starts with first-strand synthesis (PrimeScript II Reverse Transcriptase, catalog num. 2690A, Takara) where 5 µg of total RNA poly(A) + RNAs are fully reverse transcribed using a 16-mer anchored dT oligonucleotide. First-strand synthesis was performed at 42 ºC for 60 minutes. The resulting products were purified with 1.8x AMPure RNA Clean XP beads (catalog num. A63987, Beckman Coulter). After the first-strand generation, the m7G cap structure at the 5' end of the transcripts is selectively captured using the CAP-trapper technique [10,11], which leads to the removal of uncapped RNAs. The diol group on the m^7G cap is oxidized with 1M NaOAc (pH 4.5) and NaIO4 (250 mM). Tris HCl (1M, pH 8.5) was added to stop the reaction, and the whole reaction was purified with 1.8x AMPure RNA Clean XP beads. Aldehyde groups were biotinylated using a mixture containing NaOAc (1M, pH 6.0) and Biotin (Long Arm) Hydrazide (100 mM, catalog num. SP-1100, Vector Laboratories). The resulting mixture was then incubated for 30 minutes at 40ºC and purified with 1.8x AMPure RNA Clean XP beads. Single strand RNA was degraded by RNase ONE Ribonuclease (catalog num. M4261, Promega) for 30 minutes at 37ºC and purified with 1.8x AMPure RNA Clean XP beads. The m7G cap structure bound to biotin is then selected using M-270 streptavidin magnetic beads (catalog num. 65305, Thermo Fisher Scientific). M-270 streptavidin magnetic beads were equilibrated with CapTrap Lithium chloride/Tween 20-based binding buffer. The sample recovered after RNase ONE purification was bound to equilibrated M-270 streptavidin magnetic beads (incubated at 37ºC for 15 minutes), washed three times with CapTrap Tween20-based washing buffer, and released by heat shock for 5 minutes at 95ºC and quickly cooled on ice. A second release was performed, and the supernatant was also collected and mixed with the eluate from the previous release. The released sample was treated with RNase H (60 U/µl, Ribonuclease H <RNase H>, catalog num. 2150, Takara), RNase ONE (10 U/µl) and CapTrap release buffer (incubated at 37ºC for 30 minutes), purified with 1.8x AMPure XP beads (catalog num. A63881, Beckman Coulter) and concentrated by using a speed vac. After this cap-specific selection, two double-stranded linkers carrying a unique molecular identifier (UMI) are specifically ligated to the first strand cDNA [12]. Linker ligation (DNA Ligation Kit <Mighty Mix>,

catalog num. 6023, Takara) was performed in two separate steps. First, the 5' linker was ligated and purified twice to completely eliminate the non-incorporated linkers, with 1.8x AMPure XP beads, and concentrated by using a speed vac. Then, the 3' linker was ligated, purified once with 1.8x AMPure XP beads, and finally concentrated using a speed vac. The double-stranded linkers are converted into single-stranded by Shrimp Alkaline Phosphatase (1 U/µl SAP, catalog num. 78390, Affymetrix) and Uracil-Specific Excision Reagent (1 U/µl USER, catalog num. M5505L, NEB) treatment. This reaction was incubated for 30 minutes at 37ºC, 5 minutes at 95ºC, and finally placed on ice. The sample was then purified with 1.8x AMPure XP beads. After this treatment, the two linkers, which serve as priming sites for the polymerase (2x HiFi KAPA mix, catalog num. 7958927001-KK2601, Kapa), enable the synthesis of the full-length second strand. The mixture was incubated for 5 minutes at 95ºC, 5 minutes at 55ºC, 30 minutes at 72ºC and finally held at 4ºC until 1 µl Exonuclease I (20U/µl, catalog num. M0293S, NEB) was added to each sample. The sample was then incubated for 30 minutes at 37ºC and, afterward, purified twice with 1.8x and 1.4x (respectively) AMPure XP beads and finally concentrated in a speed vac. The resulting cDNA is amplified (TaKaRa LA Taq, catalog num. RR002M, Takara) via long and accurate PCR (LA PCR) protocol. In order to minimize PCR duplicates, each sample was split into two PCR-independent reactions and amplified 16 cycles with 15 seconds at 55ºC for annealing and 8 minutes at 65ºC for extension. The 2 PCR replicates were merged and purified with 1x AMPure XP beads. Samples were quantified with Qubit (Qubit 4 Fluorometer, Thermo Fisher Scientific) and quality checked with BioAnalyzer (Agilent 2100 Bioanalyzer, Agilent Technologies).

CapTrap MinION cDNA sequencing was performed with 500 ng of the cDNA sample coming from CapTrap cDNA protocol and strictly following the SQK-LSK109 adapter ligation protocol (ONT). The cDNA sequencing on the MinION platform was performed using ONT R9.4 flow cells and the standard MiniKNOW protocol.

PacBio Sequel II sequencing was performed using 500 ng of CapTrap samples following the SMRTbell™ Express Template Prep Kit 2.0 protocol.

R2C2 preparation for ONT sequencing of human and mouse

For each biological replicate, two libraries were created, a regular (non-size selected) and a size selected library of cDNA over 2 kb in length to achieve higher coverage of longer transcripts. For each RNA sample, 400 ng was used to generate full-length single-stranded cDNA using an indexed oligo(dT) primer and a template switching oligo (TSO). PCR was used to generate the second strand and amplify the library. The cDNA was then isolated by SPRI bead cleanup. For the size selected libraries, cDNA was run on a 1% low melt agarose gel. A smear in the range of 2–10 kb was excised from the gel and digested with beta-agarase, followed by SPRI bead cleanup. At this point, indexed cDNA from each biological replicate was pooled together equally. cDNA was circularized using a short DNA splint with a sequence complementary to the cDNA ends by Gibson

Assembly (NEBuilder, NEB) with a 1:1 cDNA splint ratio (100 ng each). After Gibson assembly, linear digestion (ExoI, ExoIII, and Lambda Exonuclease) was performed to eliminate non-circularized DNA. The circular Gibson assembly product was cleaned up using SPRI beads. The circularized library was used as a template for rolling circle amplification (RCA) using Phi29 polymerase and random hexamer primers. Following the RCA reaction, T7 endonuclease was used to debranch the DNA product. A DNA clean and concentrator column was used to purify the DNA. Purified RCA product was size-selected using a 1% low melt agarose gel. The main band just over the 10 kb marker was excised from the gel and digested with beta-agarase, followed by SPRI bead cleanup. The cleaned and size selected RCA product was sequenced using the ONT 1D Genomic DNA by Ligation sample prep kit (SQK-LSK109) and MinION flow cells (R9.4.1) following the manufacturer's protocol. Flow cells were nuclease flushed and reloaded with an additional library according to the ONT Nuclease Flush protocol.

cDNA preparation for ONT sequencing of human and mouse

Library preparation was done from total RNA (200ng) using SQK-PCS110 kit from ONT for PCR-cDNA sequencing. Briefly, cDNA RT adapters were annealed and ligated to full-length RNAs using NEBNext® Quick Ligation Reaction Buffer (NEB B6058) and T4 DNA Ligase (NEB M0202). Bead cleanup was done using Agencourt RNAClean XP beads. Purified RNA with CRTA top strand, RT primers, and dNTPs (NEB N0447) were incubated at RT for 15 mins to generate primer-annealed RNA. Reverse transcription and strand-switching were performed with Maxima H Minus RT enzyme in the presence of strand-switching primers at 42^0C for 90 mins, followed by heat inactivation at 85^0C for 5 mins. Reverse transcribed samples were PCR amplified using cDNA primers and LongAmp Hot Start Master Mix (NEB, M0533S). Samples were treated with NEB exonuclease I (NEB, M0293) for 15 mins at 37^0C to degrade linear single-stranded DNA, followed by enzyme inactivation at 80^0C for 15 mins. Samples were purified with Agencourt AMPure XP beads. Elution was done with 12 ul of elution buffer. 1ul of libraries was electrophoresed on TapeStation screentapes to assess size distribution, quantity, and quality of the library. FLO-MIN106D flow cells were primed with EXP-FLP002 kit reagents followed by loading of PCR-cDNA library mixed with rapid adapter F (along with sequencing buffer and loading beads). Sequencing of the library was performed without any size selection using MinION Mk1B devices and the MinKNOW software interface.

Direct RNA (dRNA) preparation for ONT sequencing of human and mouse

Direct RNA libraries were prepared from 75ug total RNA. RNA samples were poly-A selected using the NEXTFLEX poly-A kit. Purified mRNA was eluted in 12uL nuclease-free H_2O. Library preparation was performed on purified mRNA using the SQK-RNA002 kit. Direct RNA RT adapters were annealed and ligated

to full-length mRNA using T4 DNA Ligase, NEBNext Quick Ligation Reaction Buffer, and Nanopore's RNA CS. Adapter-ligated mRNA was incubated with dNTPs, 5x first-strand buffer, nuclease-free water, SuperScript IV, and 0.1M DTT to create a cDNA-RNA hybrid. This reverse-transcription (RT) step is recommended by Nanopore to reduce secondary structure formation of the mRNA as it is being sequenced. RTed RNA was purified using RNAClean XP beads. Nanopore adapters were ligated onto the RTed RNA using NEBNext Quick Ligation Reaction Buffer and T4 DNA Ligase. Following RNAClean XP bead cleanup, the libraries were eluted in 21uL of Nanopore's Elution Buffer. 1 uL of each library was quantified on the TapeStation to ensure nucleic acid concentration was at a minimum ~200ng. Libraries were loaded into MinION flow cells using the EXP-FLP002 Flow Cell Priming Kit. Libraries were sequenced for 72-hour runs.

Manatee ONT genome sequencing and assembly

Two µg of genomic DNA in a total volume of 100 µl was fragmented by the g-Tube fragmentation method (Covaris, Woburn, MA, USA) by centrifuging at 6,000x g for 1 min. The large DNA fragments were enriched by using 0.85x volume of Agencourt AMPure XP beads (Beckman Coulter, Brea, CA, USA) in the purification procedure. The enriched DNA fragments were subjected to library preparation with a Nanopore Genomic DNA Ligation Sequencing Kit (Oxford Nanopore Technologies, Oxford, UK) following the manufacturer's protocol. A total of 700 ng of the final library product was loaded on a flow cell and sequenced with a Nanopore GridION sequencer (Oxford Nanopore Technologies, Oxford, UK) for a 72-hour run. A total of 5 flow-cell runs were conducted for this project, resulting in total sequencing output of 96Gbp, represeting a 37x genome coverage. Genome assembly was performed using the OmicsBox (v1.4.11 http://www.biobam.com/omicsbox/), implementation of Flye[6], with automatic minimun overlap and further polished with Pilon using the publicy available Illumina data (BioSample SAMN00632092).

Manatee cDNA PacBio library preparation and sequencing

Approximately 280 ng of total pooled RNA were processed according to a modified IsoSeq protocol. The sample was spiked-in with the uncapped E2 RNA variant control mix (SIRVs, Lexogen, Cat # 025.03) at a 2.83% mass proportion relative to the total RNA. The resulting mixture was subjected to a globin removal step using the QIAseq FastSelect^{TM}- HRM Globin removal reagent (cat # 334376). This kit was designed for globin removal from human, mouse, and rat tissues and was found to perform with various degrees of efficiency on blood from a wide variety of samples of mammalian origin. Globin removal was performed as recommended in the QIAseq FastSelect^{TM}- -rRNA HRM -Globin Handbook (Oct 2019) in the NEBNext Ultra II section, except that the high-temperature fragmentation step was omitted. The globin removal reaction (9 µl) contained 280 ng sample (RNA plus 2.83% SIRVs), QIAseq FastSelect globin removal reagent, 2 µl NEBNext Single Cell RT

Primer Mix (NEB #6421), and 2.25 µl of NEBNext Single Cell RT buffer (4x). This mixture was prepared in a 0.2 ml PCR tube and subjected to a stepwise series of 2 min incubations each of 75°C, 70°C, 65°C, 60°C, 55°C, 37°C, and 25°C. At this point, the sample was snap-cooled by transferring it to a pre-chilled freezer block until ready for the RT and amplification steps. From this point on, cDNA synthesis was done as described in the "Protocol for Low Input RNA: cDNA Synthesis and Amplification" (NEB #E6421) starting in section 2.3. More specifically, the template "RT and Template Switching" reaction consisted of 9 µl of globin-removed RNA, 2.75 µl NEBNext Single Cell RT Buffer (4x), 1 µl of NEBNext Template Switching Oligo, 2 µl of NEBNext Single Cell RT Enzyme Mix and enough water to bring the total to 20 µl. The reaction was incubated in a thermocycler for 90 min at 42 °C and 10 min at 72 °C. The cDNA products were split into four aliquots for PCR amplification (100 µl) reactions containing 2 µl NEBNext Single Cell cDNA PCR Primer, 0.5 µl 10X NEBNext Cell Lysis Buffer, 50 µl NEBNext Single Cell cDNA PCR Master Mix, 5 µl RT and Template Switching reaction and water. Amplified cDNA was purified by AMPure, one round at 0.8 to 1.0 beads to sample ratio and one round at 0.65:1.0 ratio. The yield of amplified cDNA by this modified protocol (300-400 ng) was about 10-fold lower than the standard protocol (i.e., without globin-removal). The average cDNA size was ~1400 bp. When increased amounts of cDNA were desired, the cDNA was amplified by 5 additional PCR cycles.

Two preps obtained with the above-described protocol were pooled together, and 500 ng were loaded on an electrophoretic lateral fractionation system (ELF, SageScience). Fragments above 2.5 kb were collected, re-amplified (10 cycles), and re-pooled equimolarly with non-size-selected cDNA fragments. This re-pooled cDNA prep is referred to as "enriched cDNA_>2.5kb". Both non_enriched cDNA and enriched cDNA_>2.5kb cDNA were used for SMRT bell library construction starting with 1 µg of cDNA as described in the PacBio IsoSeq protocol 101-070-200 Version 06, September 2018. Briefly, SMRTbell adaptors (Iso-SeqTM) were added using reagents from the PacBio SMRTbell Template Prep Kit 1.0-SPv3 starting with either 200 ng (for enriched cDNA >2.5kb) or 700 ng (for non-enriched cDNA). The main steps included DNA Damage Repair, End Repair, Blunt-end ligation of SMRT bell adaptors, and ExoIII/ExoVII treatment. This procedure resulted in ~25-30% yield. Finally, libraries were eluted in 15 ul of 10 nM Tris HCl, pH 8.0. Library fragment size was estimated by the Agilent TapeStation (genomic DNA tapes), and this data was used to calculate molar concentrations.

The enriched cDNA >2.5 kb library was diffusion-loaded on a single SEQUEL SMRT cell (University of Florida, Interdisciplinary Center for Biotechnology Research (ICBR)-NGS core lab) using a loading concentration of 10 pM, 4-hr pre-extension, 20 hr movies and v3 chemistry reagents (for binding and sequencing). All other steps for sequencing were done according to the recommended protocol by the PacBio SMRT Link Sample Setup and Run Design modules (SMRT Link 6.0).

The non-enriched cDNA library was loaded on three Sequel II SMRT cells at the University of California, Irvine.

Manatee cDNA Nanopore library preparation and sequencing

One hundred and fifty nanograms of total pooled RNA were processed according to a modified ONT cDNA-PCR Sequencing protocol (cDNA-PCR-PCS109, version PCS_9085 v109 revJ Aug 14, 2019). Spike-in and globin depletion treatment was conducted as described for PacBio library preparation. In this case, the globin removal reaction (11 ul) contained: sample (RNA plus SIRVs), globin removal reagent, 1 mM dNTP, 0.2 µM VPN primer from the Nanopore cDNA synthesis protocol (i.e., in place of random primers), and 1X RT buffer (ThermoFisher). This mixture was prepared in a 0.2 ml PCR tube and submitted to a stepwise series of 2 min incubation for each of 75 °C, 70 °C, 65 °C, 60 °C, 55 °C, 37 °C, and 25 °C. At this point, the sample was snap-cooled by transferring to a pre-chilled freezer block until ready for the RT and amplification steps. From this point on, cDNA synthesis was done as described in the cDNA-PCR Sequencing (SQK-PCS109) Oxford Nanopore manual starting on page 9 (Version: PCS_90985_v109_revJ_14Aug2019). A single globin removal and cDNA synthesis reaction was split into four PCR reactions for amplification. This process resulted in approximately 2 micrograms of "full-length" cDNA with an average size of ~1800 bp. One size-selected library was constructed by loading 1500 ng of this cDNA on an electrophoretic lateral fractionation system (ELF, SageScience), collecting >2.5 kb fragments, re-amplifying (6 cycles), and re-pooling with non-size-selected cDNA fragments. Adaptor ligation and sequencing were performed according to the cDNA-PCR Sequencing (SQK-PCS109) Nanopore manual. Between 120-140 fmol of cDNA was loaded on a FLO-MIN106D (R9.4 SpotON) flow cell for sequencing on the minION device. Two runs were done on non-size-selected manatee cDNA, while only one run was done on the cDNA that had been enriched with >2.5 kb fragments. Sequencing runs were allowed to proceed for 48 hours.

Long-read data processing

Base-calling of ONT data from human, mouse, and manatee was performed with Guppy 4.2.2 and the 9.4.1 config file, with default parameters, except: --qscore_filtering --min_qscore 7 (these non-default parameters were used in all cDNA-ONT runs except for R2C2 datasets). Direct RNA base-calling was also performed with Guppy 4.4.2 with the following configurations: --qscore_filtering yes --min_qscore 7 --reverse_sequence yes --u_substitution yes

PacBio full-length non-chimeric (FLNC) reads were generated with CCS 4.2.0 (parameters: *--noPolish --minLength=10 --minPasses=3 --min-rq=0.9 --min-snr=2.5*), Lima 1.11.0 (parameters: FASTA with the

appropriate adapters *--isoseq --min-score 0 --min-end-score 0 --min-signal-increase 10 --min-score-lead 0*), and Refine 3.3.0 (parameters: *--min-polya-length 20 --require-polya*).

Consensus R2C2 reads were generated with C3POa v1.0.0 (https://github.com/rvolden/C3POa/tree/gonk) with default options.
Sequence data were provided in FASTQ format. For PacBio data, subreads are provided in unaligned BAM format, and for R2C2 data, subreads are provided in FASTQ format (**Supplementary Tables 2,4-6**).

Reference genome and annotations

For submissions of transcript models and quantification, transcript annotations and genome models corresponding to GENCODE human v38 and mouse M27 were used. Submissions of challenge predictions were expected to end in Fall 2021, prior to the release of GENCODE human v39 and mouse M28. The newly released GENCODE annotations would, therefore, be used for the evaluations. GRCh38 was the reference genome sequence for human, and GRCm39 was used for mouse. GENCODE annotations were based on these genomes. Please note that GENCODE M25 and earlier annotation releases were based on GRCm38.

Simulated data

Simulating RNA reads simply from the reference transcriptome would only allow the assessment reconstruction of known transcript models. Thus, we extended both human and mouse annotations with artificial novel transcripts. To obtain those, we mapped reference transcripts of an undisclosed mammalian organism (mouse) to the human and mouse genomes and converted the alignments into transcript models using SQANTI[13]. We then arbitrarily selected isoforms of known genes that have only canonical splice sites (GT-AG, GC-AG, and AT-AC) and merged them into human and mouse GENCODE Basic annotations. Due to a software error, and discovered after data release, 3.7% of the human and 3.0% of the mouse artificial novel transcripts were duplicates of other artificial transcripts, differing only in their transcript identifiers.

To generate realistic isoform expression profiles, we selected undisclosed human and mouse long-read datasets and quantified them simply by mapping them to the reference transcripts with minimap2 v2.17 [34]. Artificial novel isoforms were assigned arbitrary expression values. The generated expression profile was then used for simulating short and long reads. Finally, poly(A) tails were attached to the 3' end of reference transcript sequences prior to running the simulation.

To simulate reads produced by different sequencing platforms, we used existing simulation methods. Illumina 2x150bp read pairs were generated with the RSEM simulator[14] using an error model obtained from real RNA-Seq data[15] (accession number ERR1474891).

ONT reads were simulated with NanoSim[16] using pre-trained cDNA and dRNA models available in the package with an average error rate of 15.9% (4.8% substitutions, 6.0% deletions, 5.1% insertions) and 11.2% (2.8% substitutions, 5.9% deletions, 2.5% insertions) respectively. NanoSim exploits models trained on real data to produce realistic sequencing error patterns, read length distribution, and unaligned sequences at read ends typical for ONT sequencing. The complete list of Nanopore data characteristics is described in the Trans-NanoSim manuscript[16]. Manual inspection revealed that as the transcript truncation is done randomly in Trans-NanoSim, no 3'/5' bias is introduced. Thus, simulated ONT data may have slightly different coverage profiles compared to the real cDNA-ONT and dRNA-ONT data.

PacBio CCS reads were obtained with IsoSeqSim (https://github.com/yunhaowang/IsoSeqSim), which truncates input reference transcript sequences and uniformly inserts errors according to the given probabilities. Uniform error distribution appears to be a reasonable choice according to the previously developed tool for simulating genomic PacBio reads[17]. The error rate was estimated using real cDNA-PacBio CCS reads obtained in this work as 1.6% (0.4% substitutions, 0.6% deletions, 0.6% insertions). To create a realistic coverage profile for read truncation in IsoSeqSim, we used pre-computed Sequel II truncation probabilities provided along with the package.

We simulated two datasets containing reads from all three platforms listed above but with slightly different properties. Human datasets were simulated with 100 million Illumina read pairs, 30 million cDNA-ONT, and 10 million PacBio reads. Mouse datasets also contained 100 million Illumina read pairs, but equal amounts of PacBio CCS and dRNA-ONT reads were generated (20 million sequences each).

To allow users to simulate their own data, the methods described above are implemented as simple command-line scripts, which are available at https://github.com/LRGASP/lrgasp-simulation/.

CAGE data of WTC11 samples for validation of transcript 5' ends

To validate novel 5' ends, we used recently generated deep coverage CAGE data on the WTC11 line.

The 15 µg of WTC11 RNAs from each biological replicate, ENCODE BioSample Accession ENCBS944CBA and ENCBS474NOC, were used for the single strand (ss)CAGE library preparation followed in the Low Quantity

Single Strand CAGE protocol18. Briefly, the 15 µg RNAs were aliquoted to 5 µg in three tubes and reverse transcribed to cDNAs with random primers, and the capped RNA-cDNA hybrids were trapped by streptavidin beads. The single-strand cDNAs were released from the beads and ligated to the Illumina adaptors with Index, and 1080 amols of the cap-trapped single-strand cDNAs from each biological replicate were sequenced by Illumina HiSeq Rapid SBS Kits v2 (SR, 150 cycles, one lane for each).

CAGE data from WTC11 samples was produced for the validation of transcript 5' ends and was not released until after the close of the challenge submissions. CAGE data was obtained from two RNA biological replicates of WTC11, using the exact same RNA that was used for long-read sequencing.

The 15 µg of WTC11 RNAs from each biological replicate, ENCODE BioSample Accession ENCBS944CBA and ENCBS474NOC, were used for the single strand (ss)CAGE library preparation described in the published protocol[18]. Briefly, the 15 µg RNAs were aliquoted to 5 µg in three tubes and reverse transcribed to cDNAs with random primers, and the RNA-cDNA hybrids were cap-trapped by the streptavidin beads. The single-strand cDNAs were released from the beads and ligated to the Illumina adaptors with an index. 1,080 amols of the cap-trapped single-strand cDNAs from each biological replicate were sequenced by Illumina HiSeq Rapid SBS Kits v2 (SR, 150 cycles, one lane for each), producing approximately 40 million reads per sample.

QuantSeq data (3' end sequencing) from challenge 1 and 2 samples were produced for validation of 3' ends and were not released until the close of the challenge submissions. Data was obtained from two RNA biological replicates of WTC11 from the same exact RNA used for long-read sequencing.
To validate novel polyadenylation sites, we collected poly(A)-seq data using the Quant-Seq method from Lexogen, which can map poly(A) sites *de novo*.

LRGASP Data QC
Initial quality control (QC) metrics were determined for the LRGASP data (Supplementary **Fig. 44)**. Reads (ONT cDNA, dRNA, CapTrap) or consensus reads (PacBio cDNA and CapTrap and ONT R2C2) were aligned to the human or mouse genome as appropriate using minimap2 with the following parameters: -ax splice --secondary=no -G 400k. For each data type, the reads and their resulting alignments in sam format were parsed for the following parameters:

1) Number of aligned reads.
2) Number of aligned reads with adapters on both ends. For ONT dRNA this is not applicable as this workflow does not attach an adapter to the 5' end of molecules. For ONT cDNA and CapTrap, this

percentage was determined by pyChopper. For all other data types, all provided reads are assumed to have adapters on both ends as the pre-processing pipelines (lima and C3POa) discard reads otherwise.

3) Median read length, measured by the number of aligned bases (matches or mismatches).
4) Median accuracy, measured by matches / (matches+mismatches+indels).
5) Percent of aligned reads where the orientation of the reads as determined by 5' and 3' adapter sequences agree with the direction of the read alignment, determined by minimap2 through splice site context, calculated only for the subset of reads with splice alignments with the ts:A: flag in their SAM entry.
6) Percent of reads originating from spike-in molecules, determined by alignment to the SIRVomeERCC FASTA entry in the genome sequence files.
7) Pearson correlation between replicates is determined by quantifying gene expression for each replicate and calculating the Pearson r value based on those expression values.

GENCODE Benchmarks and Computational Evaluation

Full manual annotation was undertaken on 50 selected loci on both the human and mouse reference genomes. Transcript models were only annotated during this exercise based on their support from long transcriptomic datasets generated by the consortium specifically for LRGASP. No transcript annotation was based on transcriptomic data from externally produced datasets, although annotators used any publicly available orthogonal data to aid in the interpretation of aligned consortium data. For example, Fantom 5 CAGE datasets were used to help identify transcription start sites and transcript 5' ends, and RNA-seq-supported introns derived from high-throughput reanalysis pipelines such as Recount were used to support putative introns identified in the alignments of long transcriptomic data.

Manual annotation was performed according to the guidelines of the HAVANA (Human And Vertebrate Analysis aNd Annotation) group[19,20]. Transcriptomic data was aligned to the human and mouse reference genome using appropriate methods. The benefits of aligning the transcriptomic data using multiple methods were tested to reduce the impact of alignment errors and artifacts.

Annotators also used local alignment tools integrated into annotation software to give further alternative views of alignments and improve annotation accuracy. Transcript models were manually extrapolated from the alignments by annotators using the Otter annotation interface [21]. Alignments were navigated using the Blixem alignment viewer [22,23,] and, where required, visual inspection of the dot-plot output from the Dotter tool[24] was used to resolve any alignment with the genomic sequence that was unclear or absent from Blixem. Short alignments (<15 bases) that cannot be visualized using Dotter were detected using Zmap DNA Search[24] (essentially a pattern-matching tool). The construction of exon-intron boundaries required the presence of canonical splice sites (defined as GT-AG, GC-AG, and AT-AC), and any deviations from this rule were given clear explanatory tags (for example, non-canonical splice sites supported by evolutionary conservation). All

non-redundant splicing transcripts at an individual locus were used to build transcript models, and all alternatively spliced transcripts were assigned an individual biotype based on their putative functional potential. Once the correct transcript structure was ascertained, the protein-coding potential of the transcript was determined based on its context within the locus, similarity to known protein sequences, the sequences of orthologous and paralogous proteins, candidate coding regions (CCRs) identified by PhyloCSF, evidence of translation from mass spectrometry and Ribo-seq data, the presence of Pfam functional domains, the presence of possible alternative ORFs, the presence of retained intronic sequence, and the likely susceptibility of the transcript to nonsense-mediated mRNA decay (NMD).

Although the annotation of transcript functional biotype and CDS is not required of submitters, they were added to transcripts as a matter of routine manual annotation and may be used to investigate the detection or non-detection of groups of transcripts by submitters. When necessary, annotations were checked by a second annotator to ensure the completeness and consistency of annotation between the genes annotated for LRGASP and the remainder of the Ensembl/GENCODE gene set.

Procedures for Experimental Validation

Primers-JuJu bulk RT_PCR primer design tool

To facilitate the design of a large number of RT-PCR primers for validating a subset of the predicted isoforms, we developed a tool called Primers-Juju. It provides a semi-automated interface between the visualization of the transcript models in the UCSC browser and the Primer3 primer design package.

The design process starts with a UCSC track hub containing the consolidated transcript models from all pipelines. Unique features for transcripts to validate are identified by visualization. A pair of genomic regions that could contain a primer pair that would amplify the targeted transcript is manually defined. The region may be within an exon or two exons spanning a splice junction. These regions are marked using the UCSC Browser region highlight facility. Supplementary **Fig. 79a** shows an example of specifying the design region primers for a unique intron.

The genomic coordinates of the pair of regions are recorded in a spreadsheet, along with the transcript identifier. Additional transcripts that would also be amplified by the primers may be included for validation. Primers-Juju provides a command line tool that takes the specification spreadsheet with multiple targets and transcript annotations and does primer design and validation. The input specifications are validated against the targeted transcripts, with minor adjustments for inexact bounds. The sequence for the transcripts is obtained from the genomic sequence of the exons, and the regions are converted to transcript coordinates.

The Primer3 programmatic library is given each transcript sequence and region pair and will attempt to design a stable primer pair to amplify this transcript, returning up to five possible primer pairs per region. The in-silico PCR command line tool is used to check for potential off-target primer pairs. Queries are done against the genome sequences and transcriptome sequences, which consist of the known annotations as well as the LRGASP consolidated transcripts.

Primers-Juju generates additional tracks for the hub with primer pairs and amplicon sequences (Supplementary **Fig. 79b**). It also produces reports and recommends the most stable primer with no off-target hits to order.

Semi-automated primer selection process with Primers-Juju

The aggregate of all transcript models from all pipelines underwent visualization in a UCSC Browser track hub [7] to design primers that target specific transcript features. The process identified uniquely mapping sub-segments of isoforms and selected flanking 5' end and 3' end regions for primer design via the "Highlight" function within the UCSC Browser [8]. The system then recorded the genomic coordinates of the regions and transcript identifiers.

Primers-JuJu (**Supplementary Methods**) processed the primer region specifications, obtained the DNA sequence for the predicted RNA, and employed Primer3 [9] for primer design. The primer pairs are evaluated for off-target genome and transcriptome hits using In-Silico PCR [10]. The resulting primer pairs are then added to the track hub for visualization (see **Supplementary Data 19 f**or the list of primers).

cDNA synthesis

Replicates 2 and 3 of the same WTC11 total RNA aliquots that were used as input for the sequencing runs were used for cDNA. Approximately 1.3 ug of total RNA from each replicate was converted to cDNA using the NEBNext Single Cell/Low Input cDNA Synthesis & Amplification Module kit, with 14 cycles of PCR being performed.

PCR of targets, QC, and amplicon pooling

Aliquots of 2uL cDNA (~0.45 ug) were used as a template for PCR reactions in which isoform-specific or isoform-partially-specific primers were used for amplification. One round of PCRs was done using the Replicate 2 cDNA, and a second round was done using the Replicate 3 cDNA. We used KAPA HiFi HotStart Ready Mix polymerase due to its high fidelity (Roche, Cape Town, South Africa). A touch-down PCR method was employed that involved a 95°C denaturing step for 3 minutes, followed by another denaturing at 98°C for 20 seconds, an annealing step starting at 70°C for 15 seconds and followed by an extension step at 72°C for 2 minutes. The denaturing step was at 98°C, and the following steps were repeated for 12 cycles; at each cycle, the annealing temperature went down 1°C degree. Then, we performed a PCR with a single denaturing

temperature cycle at 98°C, annealing at 57°C, and extension at 72°C, maintaining the same duration as described above and repeating these steps for 21 cycles. We performed a final extension at 72°C for 5 minutes.

5 uL of each PCR reaction and 10 uL of water were analyzed on a 1% agarose e-gel with SYBR Safe. Bands were imaged and manually annotated. We found excellent agreement between the predicted and experimentally measured product length, with more than 60% of the bands matching within XX bp. 3 uL of each PCR product was combined to create a pool of all amplicons derived from PCR of both Replicate 2 and 3, in batches (**Supplementary Table 12, Supplementary Data 20**). After quantification of the amplicon pool via Qubit, they were subjected to ONT and PacBio sequencing.

Libraries for ONT sequencing were prepared using an SQK-LSK114 kit and 300ng of pooled cDNA library was loaded on an R10.4.1 flow cell and sequenced at 260bps/sec. The run was stopped after 21h with ~5.6e6 reads with an N50 of 1.2 kbp. Nanopore data was basecalled with guppy version 6.2.11 with the high-accuracy configuration (dna_r10.4.1_e8.2_260bps_hac.cfg). The reads, aligned to the human genome assembly, were deposited in the Sequence Read Archive (SRA) under the accession number SRR23881262.

For PacBio sequencing, 492 ng of the cDNA was combined with 123 ng of the manatee cDNA (pooled in a 1:5 ratio manatee: WTC-11 sample; see description in the section below) and subsequently converted into an SMRTBell library. The library was sequenced on a PacBio Sequel II, generating HiFi reads. The reads, which were aligned to the human and manatee genome assemblies, have been deposited in the SRA under the accession numbers SRR24680098 and SRR24680099, respectively.

Computational Pipeline Description from Submitters

Challenge 1

Name: Bambu
Description: Bambu trains a transcript discovery model on each sample using the known reference annotation to predict if novel aligned reads are likely to represent full-length transcripts. This optimizes several parameters relevant to transcript discovery and reduces this down to a single tunable parameter, which is customized to the specific sample transcriptome, the novel discovery rate (NDR). By ranking novel transcripts with the NDR, Bambu can extend the annotations across a large range of sensitivity and precision.
Version: The development version of Bambu 0.9.1 was used during LRGASP.
Team: Göke, Genome Institute of Singapore
URL: https://github.com/GoekeLab/bambu and https://bioconductor.org/packages/bambu/
Citations: Chen, Y., Sim, A., Wan, Y.K. *et al.* Context-aware transcript quantification from long-read RNA-seq data with Bambu. *Nat Methods* (2023). https://doi.org/10.1038/s41592-023-01908-w

Config: Bambu was run with the following parameters (NOTE: many of these parameters are deprecated in the updated version of Bambu) min.txScore.multiEx
on = 0, min.txScore.singleExon = 1, max.txNDR = 0.2, min.geneScore = 0, min.samp
leNumber = 1, remove.subsetTx = FALSE, min.readFractionByGene = 0). Please see the documentation for best practices when using Bambu's latest version.
Notes: Bambu uses an NDR threshold, which allows the user to influence the sensitivity and precision of novel transcripts. By default, Bambu is calibrated to report a precision selection of novel transcripts, with a threshold of 0.2 being used in the LRGASP challenge. However a higher (and less stringent) NDR value can be used to greatly increase the number of transcripts reported by Bambu.
Funding: A.S, Y.C, J.J.X.L, Y.K.W, and J.G are supported by funding from the Agency for Science, Technology and Research (A*STAR)and the National Medical Research Council (NMRC).

Name: FLAIR
Description: FLAIR is a tool for RNA isoform exploration with long reads with the optional pairing of short reads. FLAIR contains modules for correcting noisy reads, isoform definition, isoform quantification, and analysis of alternative splicing in long read data.
Version: 2
Team: Brooks Lab, University of California, Santa Cruz
URL: https://github.com/BrooksLabUCSC/flair/
Citations: Detecting haplotype-specific transcript variation in long reads with FLAIR2
Alison D Tang, Eva Hrabeta-Robinson, Roger Volden, Christopher Vollmers, Angela N Brooks
bioRxiv 2023.06.09.544396; https://doi.org/10.1101/2023.06.09.544396
Config: We ran the FLAIR2 isoform discovery pipeline using the flair-collapse module with the --annotation_reliant, --check_splice, and --stringent parameters. The short-and-long submissions used short-read data to identify confident splice junctions.
Notes: FLAIR2 run with the --annotation_reliant argument invokes an alignment of the reads to an annotated transcriptome first, followed by novel isoform detection. When including --check_splice, this enforces higher quality matching specifically around each splice site for read-to-isoform assignment steps.
Funding: A.D.T. is supported by NIH NHGRI F31 HG010999. This work was also supported by NIH NIGMS R35GM138122 (A.N.B.).

Name: FLAMES
Description: A tool developed for full-length transcript quantification, mutation and splicing analysis of long-read RNA-seq data.
Version: 0.1.0
Team: Ritchie Lab, Walter and Eliza Hall Institute of Medical Research
URL: https://github.com/LuyiTian/FLAMES
Citations: Tian, L., Jabbari, J.S., Thijssen, R. *et al.* Comprehensive characterization of single-cell full-length isoforms in human and mouse with long-read sequencing. *Genome Biol* **22**, 310 (2021). https://doi.org/10.1186/s13059-021-02525-6
Config: The following parameters were adjusted in the configuration file for LRGASP: has_UMI:false, Min_sup_cnt:10, Min_cnt_pct:0.01, strand_specific:1, remove_incomp_reads:5, no_flank:true, min_tr_coverage:0.75, and min_read_coverage:0.75.

Notes: FLAMES provides a default set of parameters, which can be changed in the configuration JSON file. The 'pipeline_parameters' section specifies the steps to be executed in the pipeline (all by default). The 'isoform_parameters' section determines the results of isoform detection.
Funding: FLAMES development was supported by funding from the Chan Zuckerberg Initiative DAF, an advised fund of the Silicon Valley Community Foundation (Grant No. 2019-002443 to M.E.R.) and Australian National Health and Medical Research Council (NHMRC) Investigator Grant (2017257 to M.E.R.).

Name: Iso_IB
Description: An IsoSeq evidence-based approach to predict gene models, alternative splices, and isoforms using a custom path from the cDNA-cupcake workflow
Version: CD-HIT: 4.8.1; minimap2: 2.17-r974; collapse_isoforms_by_sam.py: 22.0.0; gffread: v0.12.7;
Team: Integrative Bioinformatics, National Institute of Environmental Health Sciences
URL: https://github.com/weizhongli/cdhit
Config: The following parameters were used for each step of the workflow CD-HIT: cd-hit-est -M 0 -c 0.99 -G 0 -aL 0.90 -AL 100 -aS 0.99 -AS 30; minimap2: -ax splice -t 40 -uf --secondary=no -C5; collapse_isoforms_by_sam.py --fq -s; gffread -E -T -o-;
Notes: The reference workflow and repo for cDNA_cupcake can be found at https://github.com/Magdoll/cDNA_Cupcake. The conda instance can also be found at https://github.com/PacificBiosciences/IsoSeq. In this workflow, no reference gene model/isoforms/alternative splicing prediction/annotation was used to enhance or validate the models. The resulting set was run in de novo mode, solely on the basis of evidence sequences obtained from the IsoSeq sequencing data provided by the consortium.
Funding: This work was supported by the Intramural Research Program of the National Institute of Environmental Health Sciences ZIC ES103371

Name: IsoQuant
Description: IsoQuant is a reference-based approach for transcript discovery and quantification using long RNA reads. Since version 3.0 it also supports annotation-free transcript discovery.
Version: 2.0.0
Team: Center for Algorithmic Biotechnology, Saint Petersburg State University
URL: https://github.com/ablab/IsoQuant
Citations: Prjibelski, A.D., Mikheenko, A., Joglekar, A. *et al.* Accurate isoform discovery with IsoQuant using long reads. *Nat Biotechnol* (2023). https://doi.org/10.1038/s41587-022-01565-y
Config: --data_type nanopore for ONT data, --data_type pacbio_ccs for PacBio data
Notes: The tool can be installed via conda. Reads can be provided in BAM or in FASTQ format. In the latter case they will be automatically mapped using minimap2.
Funding: St. Petersburg State University (grant ID: 94030965), European Research Council (ERC) under the European Union's Horizon 2020 research and innovation programme (grant agreement No. 851093, SAFEBIO)

Name: IsoTools
Description: IsoTools is a Python module for Long Read Transcriptome Sequencing (LRTS) analysis, providing transcriptome reconstruction, filtering, and quantification, along with explorative analysis and alternative splicing detection both on isoform as well as splice-site level and differential analysis. IsoTools

version > 0.3.2 also includes functional annotation and interpretation including ORF prediction, NMD prediction, and domain annotation.
Version: 0.2.5
Team: Herwig Lab, Max Planck Institute for Molecular Genetics
URL: https://isotools.readthedocs.io
Citations: Matthias Lienhard and others, IsoTools: a flexible workflow for long-read transcriptome sequencing analysis, *Bioinformatics*, 2023; btad364, https://doi.org/10.1093/bioinformatics/btad364
Config: We filtered transcripts based on splice-site matches and read-count thresholds. Transcripts with novel splice sites must be supported by at least five reads and at least 5% of the total gene read support and are discarded if they contain more than 50% A content downstream or a direct repeat of length 6 or longer at a novel splice site. Transcripts with all splice-sites matching reference splice-sites need support from at least 2 reads and at least 2% of the total gene read support. For more configuration details, see https://github.molgen.mpg.de/lienhard/LRGASP_IsoTools.
Notes: The filtering strategy used in the IsoTools pipeline significantly impacts the number and nature of identified transcripts. While the strategy described above was developed specifically for the challenge, it may not be optimal for all datasets and research questions. Please consult the documentation for detailed instructions on using IsoTools for your specific dataset and research question. Note that the filtering syntax has been improved since pre-release version 0.2.5 was used for the submission.
Funding: This work was supported by the German Research Foundation (DFG) with the grant HE4607/7-1 and the Federal Ministry of Education and Research with the grant SafetyNet (161L0242A).

Name: LyRic
Description: An end-to-end workflow for long-read based transcriptome annotation, visualization, and analysis
Version: v1.0.4
Team: Guigó Lab, Centre de Regulació Genòmica
URL: https://github.com/guigolab/LyRic
Citations: CapTrap-Seq: A platform-agnostic and quantitative approach for high-fidelity full-length RNA transcript sequencing
Silvia Carbonell-Sala, Julien Lagarde, Hiromi Nishiyori, Emilio Palumbo, Carme Arnan, Hazuki Takahashi, Piero Carninci, Barbara Uszczynska-Ratajczak, Roderic Guigo
bioRxiv 2023.06.16.543444; https://doi.org/10.1101/2023.06.16.543444
Config: LyRic was run with default parameters except as specified in its LRGASP configuration file, available at https://github.com/guigolab/LyRic/blob/master/config_LRGASP.json. The LyRic working directory was staged for LRGASP data processing using the *setup_LRGASP.sh* script (https://github.com/guigolab/LyRic/blob/master/setup_LRGASP.sh) All PacBio sequencing data were re-processed from BAM files using the *pb_gen* pipeline (https://github.com/guigolab/pb_gen) with default parameters, except for adapter sequences ('*PB_ADAPT*' parameter), which were changed according to LRGASP organizers' adapter sequence specifications. PacBio and ONT FASTQ files were processed by LyRic using a *filter_SJ_Qscore* of 30 and 10, respectively. Briefly, *filter_SJ_Qscore* represents the minimum average Phred sequencing quality of read sequences +/- 3 nts around all their splice junctions for a spliced read to be considered for transcript model building. See LyRic documentation

(https://guigolab.github.io/LyRic/documentation.html), and input LRGASP sample annotation file (https://github.com/guigolab/LyRic/blob/master/sample_annotations_LRGASP.tsv) for more details.
Reads were merged into transcript models with the *tmerge* utility (https://github.com/guigolab/tmerge) using the following two-step nested approach. First, reads were merged separately within replicates, requiring a minimum of two reads supporting each transcript model. The resulting transcript models were then merged again, this time across all three replicates of each LRGASP sample, requiring replicate-specific transcript models to be detected at least once in every replicate.
Notes: LyRic's output transcript models are completely agnostic to any pre-existing reference annotation. In other words, LyRic does not adjust the coordinates of the transcript models it produces based on a reference annotation.
Funding: National Human Genome Research Institute of the US National Institutes of Health (grant 2U24HG007234-09). We acknowledge the support of the Spanish Ministry of Science and Innovation to the EMBL partnership, Centro de Excelencia Severo Ochoa, and CERCA Programme / Generalitat de Catalunya.

Name: Mandalorion
Description: Mandalorion uses PacBio or ONT-based R2C2 consensus reads. It aligns those reads using minimap2, then parses those alignments to generate models of isoforms. Mandalorion then generates read-based consensus sequences for each isoform using pyabpoa and racon tools. Mandalorion then aligns these isoform consensus sequences and filters the isoforms based on these alignments and their abundance. The final isoforms are reported as both FASTA and PSL/GTF files.
Version: v3.6
Team: Vollmers Lab, University of California, Santa Cruz
URL: https://github.com/christopher-vollmers/Mandalorion
Citations: Identifying and quantifying isoforms from accurate full-length transcriptome sequencing reads with Mandalorion, Roger Volden, Kayla Schimke, Ashley Byrne, Danilo Dubocanin, Matthew Adams, Christopher Vollmers bioRxiv 2022.06.29.498139 https://doi.org/10.1101/2022.06.29.498139
Config: all runs were performed using the "-R 3" and "-I 150" flags, setting the minimum read number and minimum length of isoforms, respectively.
Notes: Mandalorion only uses individual splice sites in any provided GTF annotation file. It discards information on how splice sites are connected into splice junctions. It also ignores annotated transcription start sites and poly(A) sites when constructing isoforms. Because Mandalorion doesn't heavily rely on information in the annotation file, performance is very similar for novel or annotated isoforms, and the ends of identified isoforms will agree with read alignments rather than annotated TSS and poly(A) sites. Another consequence of not relying heavily on an annotation file is that Mandalorion will not "assemble" isoforms that are longer than the provided reads, which is obvious for some of the "long SIRV" data.
Funding: NIH/NIGMS R35GM133569 to Christopher Vollmers

Name: Spectra
Description: Spectra is a tool to build gene models based on full-length cDNA reads, not fragmented or incomplete ones, through a guide of genome alignments. The resulting gene models are entirely (end-to-end) supported with one or more observations of reads.
Version: v0.1a
Team: Hideya Kawaji, Tokyo Metropolitan Institute of Medical Science

URL: https://github.com/hkawaji/spectra
Config: PacBio's consensus sequence is computed with a set of helper scripts bundled in the repository.
Notes: The development of this tool was motivated by the notion that the read counts of long RNA molecules are depleted in the contributed data sets, even with the best protocol. Discoveries supported by experimental evidence were maximized by selecting high-quality data and setting a minimum read count threshold.
Funding: AMED (Grant Number 21kk0305013h0002).

Name: StringTie2
Description: StringTie2 is a guided transcriptome assembler, able to assemble either short or long RNA-seq read data, even in the absence of a reference annotation. Since version 2.2.0 it also capable of handling mixed transcriptomic data that includes both short and long RNA-seq reads sequenced from the same sample.
Version: 2.2.1
Team: Pertea Lab, Johns Hopkins University
URL: https://github.com/gpertea/stringtie
Citation: Kovaka, S., Zimin, A.V., Pertea, G.M. *et al.* Transcriptome assembly from long-read RNA-seq alignments with StringTie2. *Genome Biol* **20**, 278 (2019). https://doi.org/10.1186/s13059-019-1910-1
Config: -L option for long read alignments, or --mix for both short and long read alignments
Notes: An up-to-date documentation and usage manual can be consulted at https://ccb.jhu.edu/software/stringtie/index.shtml**.**
Funding: NSF grant DBI-1759518

Name: TALON_LAPA
Description: Minimap2, STAR, TranscriptClean, TALON, LAPA

- TranscriptClean: TranscriptClean corrects common long-read sequencing artifacts such as microindels and mismatches.
  - Noncanonical splice junctions, if not provided in the input set of splice junctions, will be corrected to the nearest canonical splice sites in places where possible; otherwise, they are discarded.

- TALON: TALON annotates long reads to their transcripts of origin, quantifies the expression of annotated transcripts, and filters novel transcript models based on reproducibility and evidence of internal priming
  - Any read meeting coverage and identity filters with new splice sites will constitute a novel model.
  - Any read with an intron chain that matches that of a reference model or a novel model that's already been cataloged in the database that also 5'/3' ends within a certain distance of the cataloged model will be assigned to that model. Otherwise, it will be used to create a new model with new 5'/3' ends.
  - Transcripts are quantified simply by counting the number of reads that belong to each cataloged transcript model.
  - Unannotated (novel) transcripts are filtered for reproducibility and for those that display evidence of internal priming (see settings used in the config section)

- LAPA: LAPA is used to refine the 3' end calls made by TALON.
    - In the analysis, we called poly(A)-sites with LAPA and updated the 3' ends of the transcripts based on those poly(A)-sites with the most likely poly(A)-site assigned to each intron chain (transcript) based on the number of reads ending in the poly(A)-cluster.
    - Transcripts with low expression may not map to any poly(A)-cluster. In this case, we chose the longest end as a 3' end of the transcript.

Version: LAPA 0.0.1
Team: Mortazavi Lab, University of California, Irvine
URL: https://github.com/lh3/minimap2, https://github.com/mortazavilab/TranscriptClean/, https://github.com/mortazavilab/TALON/, https://github.com/mortazavilab/lapa/, https://github.com/mortazavilab/lrgasp-talon/
Citations: Analysis of alternative polyadenylation from long-read or short-read RNA-seq with LAPA
Muhammed Hasan Çelik, Ali Mortazavi
bioRxiv 2022.11.08.515683; https://doi.org/10.1101/2022.11.08.515683
A technology-agnostic long-read analysis pipeline for transcriptome discovery and quantification
Dana Wyman, Gabriela Balderrama-Gutierrez, Fairlie Reese, Shan Jiang, Sorena Rahmanian, Stefania Forner, Dina Matheos, Weihua Zeng, Brian Williams, Diane Trout, Whitney England, Shu-Hui Chu, Robert C. Spitale, Andrea J. Tenner, Barbara J. Wold, Ali MortazavibioRxiv 672931; https://doi.org/10.1101/672931
Config: All arguments are default unless otherwise specified
TranscriptClean: --canonOnly --spliceJns {short read + GENCODE splice junctions for the short+long, otherwise just GENCODE splice junctions)
talon_label_reads: --ar 20
talon_initialize_database: --5p 500 --3p 300
talon: --cov 0.9 --identity 0.8
talon_filter_transcripts: --maxFracA 0.5 --minCount 2 minDatasets 2
Notes:

- Known problems with submission:
    - For the ONT reads (dRNA, cDNA, CapTrap), the adapters were not removed in contrast to the PacBio reads. This affected our coverage and identity filters, which require certain mapping rates to include each read. Thus, many ONT reads with the adapters still on had large unalignable regions and were discarded, leading to erroneous results.
    - For the ONT cDNA and cDNA CapTrap, the reads are unstranded, while our tools expect only 5'-3' oriented alignments as input. This gave us a lot of antisense transcripts that should have been real transcripts and were discarded by the filter that takes novelty into account.
    - Spike-in models were not included in our reference transcriptome annotation. Therefore, we treated them like novel transcripts and were subject to the reproducibility filter, leading to many of them being erroneously discarded. Our performance on the spike-ins represents our efforts to do reference-free annotation and should be interpreted as such. This likely explains the discrepancies between our performance on the spike-in and the simulated data.
    - TALON labels transcript models using the SQANTI novelty categories. Users have the option of filtering out all ISMs, even those that pass the reproducibility and internal priming filters. Internally, we have found that our precision on spike-ins is much better when we do so (data

available on request). However, for this submission, we did not, leading to the expected high levels of ISMs reported in our dataset.
  - In general, we ran all our tools with default parameters regardless of the protocol, which is similar to what the average user can do.

Funding: NHGRI UM1HG009443

Challenge 2

Name: Bambu
Description: Bambu performs quantification after performing transcript discovery.
Version: The development version of Bambu 0.9.1 was used during LRGASP.
Team: Göke, Genome Institute of Singapore
URL: https://github.com/GoekeLab/bambu and https://bioconductor.org/packages/bambu/
Citations: Chen, Y., Sim, A., Wan, Y.K. *et al.* Context-aware transcript quantification from long-read RNA-seq data with Bambu. *Nat Methods* (2023). https://doi.org/10.1038/s41592-023-01908-w
Config: Bambu was run with the following parameters (NOTE: many of these parameters are deprecated in the updated version of Bambu): NDR = 0.2, and opt.em=list(degradationBias = TRUE). Please see the documentation for best practices when using Bambu's latest version.
Notes: We use a development version of Bambu for quantification in LRGASP challenge. Now Bambu uses an improved quantification model. We recommend using the latest version of Bambu.
Funding: A.S, Y.C, J.J.X.L, Y.K.W, and J.G are supported by funding from the Agency for Science, Technology, and Research (A*STAR) and the National Medical Research Council (NMRC).

Name: FLAIR
Description: FLAIR is a tool for RNA isoform exploration with long reads with the optional pairing of short reads. FLAIR contains modules for correcting noisy reads, isoform definition, isoform quantification, and analysis of alternative splicing in long read data.
Version: 2
Team: Brooks Lab, University of California, Santa Cruz
Citations: Detecting haplotype-specific transcript variation in long reads with FLAIR2
Alison D Tang, Eva Hrabeta-Robinson, Roger Volden, Christopher Vollmers, Angela N Brooks
bioRxiv 2023.06.09.544396; https://doi.org/10.1101/2023.06.09.544396
URL: https://github.com/BrooksLabUCSC/flair
Config: We ran the flair-quantify module with the --stringent and --tpm parameters.
Notes: None
Funding: A.D.T. is supported by NIH NHGRI F31 HG010999. This work was also supported by NIH NIGMS R35GM138122 (A.N.B.).

Name: FLAMES
Description: A tool developed for full-length transcript quantification, mutation, and splicing analysis of long-read RNA-seq data.

Version: 0.1.0
Team: Ritchie Lab, Walter and Eliza Hall Institute of Medical Research
URL: https://github.com/LuyiTian/FLAMES
Citations: Tian, L., Jabbari, J.S., Thijssen, R. *et al.* Comprehensive characterization of single-cell full-length isoforms in human and mouse with long-read sequencing. *Genome Biol* **22**, 310 (2021). https://doi.org/10.1186/s13059-021-02525-6
Config: The following parameters were adjusted in the configuration file for LRGASP: has_UMI:false, Min_sup_cnt:10, Min_cnt_pct:0.01, strand_specific:1, remove_incomp_reads:5, no_flank:true, min_tr_coverage:0.75, and min_read_coverage:0.75.
Notes: FLAMES provides a default set of parameters, which can be changed in the configuration JSON file. The 'pipeline_parameters' section specifies the steps to be executed in the pipeline. The 'isoform_parameters' section determines the results of isoform detection.
Funding: FLAMES development was supported by funding from the Chan Zuckerberg Initiative DAF, an advised fund of Silicon Valley Community Foundation (Grant No. 2019-002443 to M.E.R.) and Australian National Health and Medical Research Council (NHMRC) Investigator Grant (2017257 to M.E.R.).

Name: IsoQuant
Description: IsoQuant is a reference-based approach for transcript discovery and quantification using long RNA reads. Since version 3.0 it supports annotation-free transcript discovery.
Version: 2.0.0
Team: Center for Algorithmic Biotechnology, Saint Petersburg State University
URL: https://github.com/ablab/IsoQuant
Citations: Prjibelski, A.D., Mikheenko, A., Joglekar, A. *et al.* Accurate isoform discovery with IsoQuant using long reads. *Nat Biotechnol* (2023). https://doi.org/10.1038/s41587-022-01565-y
Config: --data_type nanopore for ONT data, --data_type pacbio_ccs for PacBio data
Notes: The tool can be installed via conda. Reads can be provided in BAM or in FASTQ format. In the latter case they will be automatically mapped using minimap2.
Funding: St. Petersburg State University (grant ID: 94030965), European Research Council (ERC) under the European Union's Horizon 2020 research and innovation programme (grant agreement No. 851093, SAFEBIO)

Name: IsoTools
Description: IsoTools is a Python module for Long Read Transcriptome Sequencing (LRTS) analysis, providing transcriptome reconstruction, filtering, and quantification, along with explorative analysis alternative splicing detection both on isoform as well as splice-site level and differential analysis. IsoTools version > 0.3.2 also includes functional annotation and interpretation, including ORF prediction, NMD prediction, and domain annotation.
Version: 0.2.5
Team: Herwig Lab, Max Planck Institute for Molecular Genetics
URL: https://isotools.readthedocs.io
Citations: Matthias Lienhard and others, IsoTools: a flexible workflow for long-read transcriptome sequencing analysis, *Bioinformatics*, 2023; btad364, https://doi.org/10.1093/bioinformatics/btad364
Config: To account for transcript length distribution differences between PacBio Isoseq data and reference annotation, TPM values were normalized using a lognormal model fit to the reference and observed transcript

length distributions.The quotient of the two distributions served as the normalization factor. Due to poor fit in the left tail, the factor was set constant for the first 1% percentile of the observed transcript length model.
For more configuration details, see https://github.molgen.mpg.de/lienhard/LRGASP_IsoTools.
Funding: This work was supported by the German Research Foundation (DFG) with the grant HE4607/7-1 and the Federal Ministry of Education and Research with the grant SafetyNet (161L0242A).

Name: NanoSim
Description: NanoSim is a fast and scalable read simulator that captures the technology-specific features of ONT data and allows for adjustments upon improvement of nanopore sequencing technology.
Version: 3.0.0 (See "Notes" below)
Team: Birol Lab, University of British Columbia, Vancouver
URL: https://github.com/bcgsc/lrgasp_nanosim
Citations: Chen Yang and others, NanoSim: nanopore sequence read simulator based on statistical characterization, *GigaScience*, Volume 6, Issue 4, April 2017, gix010, https://doi.org/10.1093/gigascience/gix010
Config: python read_analysis.py quantify -t THREADS -e trans -rt REFERENCE_TRANSCRIPTS.fasta -i READS.fastq -o nanosim
Notes: Please note that this is a completely separate repository that was branched from the primary repository at https://github.com/bcgsc/NanoSim.
Funding: This work was supported by Genome Canada and Genome BC (281ANV) and by the National Human Genome Research Institute of the National Institutes of Health (R01HG007182). Scholarship funding was provided by the University of British Columbia and the Natural Sciences and Engineering Research Council of Canada.

Name: TALON_LAPA
Description: Minimap2, STAR, TranscriptClean, TALON, LAPA

- TranscriptClean: TranscriptClean corrects common long-read sequencing artifacts such as microindels and mismatches.
    - Noncanonical splice junctions, if not provided in the input set of splice junctions, will be corrected to the nearest canonical splice sites in places where possible. Otherwise they are discarded.

- TALON: TALON annotates long reads to their transcripts of origin, quantifies the expression of annotated transcripts, and filters novel transcript models based on reproducibility and evidence of internal priming
    - Any read meeting coverage and identity filters with new splice sites will constitute a novel model
    - Any read with an intron chain that matches that of a reference model or a novel model that's already been cataloged in the database that also 5'/3' ends within a certain distance of the cataloged model will be assigned to that model. Otherwise, it will be used to create a new model with new 5'/3' ends.
    - Transcripts are quantified simply by counting the number of reads that belong to each cataloged transcript model.

    - Unannotated (novel) transcripts are filtered for reproducibility and for those that display evidence of internal priming (see settings used in the config section)

- LAPA: LAPA is used to refine the 3' end calls made by TALON.
    - In the analysis, we called poly(A)-sites with LAPA and updated the 3' ends of the transcripts based on those poly(A)-sites with the most likely poly(A)-site assigned to each intron chain (transcript) based on the number of reads ending in the poly(A)-cluster.
    - Transcripts with low expression may not map to any poly(A)-cluster. In this case, we chose the longest end as a 3' end of the transcript.

Version: LAPA 0.0.1
Team: Mortazavi Lab, University of California, Irvine
URL: https://github.com/lh3/minimap2, https://github.com/mortazavilab/TranscriptClean/, https://github.com/mortazavilab/TALON/, https://github.com/mortazavilab/lapa/, https://github.com/mortazavilab/lrgasp-talon/
Citations: Analysis of alternative polyadenylation from long-read or short-read RNA-seq with LAPA
Muhammed Hasan Çelik, Ali Mortazavi
bioRxiv 2022.11.08.515683; https://doi.org/10.1101/2022.11.08.515683
A technology-agnostic long-read analysis pipeline for transcriptome discovery and quantification
Dana Wyman, Gabriela Balderrama-Gutierrez, Fairlie Reese, Shan Jiang, Sorena Rahmanian, Stefania Forner, Dina Matheos, Weihua Zeng, Brian Williams, Diane Trout, Whitney England, Shu-Hui Chu, Robert C. Spitale, Andrea J. Tenner, Barbara J. Wold, Ali Mortazavibio Rxiv 672931; https://doi.org/10.1101/672931
Config:
All arguments are default unless otherwise specified.
TranscriptClean: --canonOnly --spliceJns {short read + GENCODE splice junctions for the short+long, otherwise just GENCODE splice junctions)
talon_label_reads: --ar 20
talon_initialize_database: --5p 500 --3p 300
talon: --cov 0.9 --identity 0.8
talon_filter_transcripts: --maxFracA 0.5 --minCount 2 minDatasets 2
Notes:

- Known problems with submission:
    - For the ONT reads (dRNA, cDNA, CapTrap), the adapters were not removed in contrast to the PacBio reads. This affected our coverage and identity filters, which require certain mapping rates to include each read. Thus, many ONT reads with the adapters still on had large unalignable regions and were discarded, leading to erroneous results.
    - For the ONT cDNA and cDNA CapTrap, the reads are unstranded, while our tools expect only 5'-3' oriented alignments as input. This gave us a lot of antisense transcripts that should have been real transcripts and were discarded by the filter that takes novelty into account.
    - Spike-in models were not included in our reference transcriptome annotation. Therefore, we treated them like novel transcripts and were subject to the reproducibility filter, leading to many of them being erroneously discarded. Our performance on the spike-ins represents our efforts to

do reference-free annotation and should be interpreted as such. This likely explains the discrepancies between our performance on the spike-in and the simulated data.

  - TALON labels transcript models using the SQANTI novelty categories. Users have the option of filtering out all ISMs, even those that pass the reproducibility and internal priming filters. Internally, we have found that our precision on spike-ins is much better when we do so (data available on request). However, for this submission, we did not, leading to the expected high levels of ISMs reported in our dataset.
  - In general, we ran all our tools with default parameters regardless of the protocol, which is similar to what the average user can do.

Funding: NHGRI UM1HG009443

Name: RSEM
Description: For comparison against long-read quantification, the LRGASP organizers ran RSEM against the GENCODE reference transcripts.
Version: RSEM v1.3.3
Team: Dewey Lab
URL: https://deweylab.github.io/RSEM/
Citations: Li, B., Dewey, C.N. RSEM: accurate transcript quantification from RNA-Seq data with or without a reference genome. *BMC Bioinformatics* **12**, 323 (2011). https://doi.org/10.1186/1471-2105-12-323
Config:

- Preparing Reference Sequences
  RSEM-1.3.3/rsem-prepare-reference
  -gtf reference annotation(GTF file) \
  --bowtie2 \
  reference genome(fasta file) \
  rsem_index(index path)

- Calculating Expression Values
  RSEM-1.3.3/rsem-calculate-expression \
  -p 20 \
  --bowtie2 \
  --sort-bam-by-coordinate \
  --sort-bam-memory-per-thread 10G \
  --paired-end paired_end_1.fq paired_end_2.fq \
  rsem_index(index_path) \
  quantification_results(output_quantification_result_path)

Notes:

RSEM quantification consisted of two main steps:
(1) Preparing Reference Sequences;
(2) Calculating Expression Values.
The specific parameter design is described above. The versions of RSEM and bowtie2 are:
Bowtie2: version 2.4.1

RSEM: v1.3.3

Challenge 3

Name: Bambu

Description: Bambu uses a reference annotation trained model to predict if novel aligned reads are likely to represent full-length transcripts. As Challenge 3 involved not using reference annotations, Bambu instead uses its internal pre-trained model.

Version: The development version of Bambu 0.9.1 was used during LRGASP. Bambu's current version is 3.0.8.

Team: Göke, Genome Institute of Singapore

URL: https://github.com/GoekeLab/bambu and https://bioconductor.org/packages/bambu/

Citations: Chen, Y., Sim, A., Wan, Y.K. *et al.* Context-aware transcript quantification from long-read RNA-seq data with Bambu. *Nat Methods* (2023). https://doi.org/10.1038/s41592-023-01908-w

Config: Bambu was run with the following parameters (NOTE: many of these parameters are deprecated in the updated version of Bambu) min.txScore.multiExon = 0, min.txScore.singleExon = 1, max.txNDR = 0.7, min.geneScore = 0, min.sampleNumber = 1, remove.subsetTx = FALSE, min.readFractionByGene = 0. Please see the documentation for best practices when using Bambu's latest version.

Notes: To achieve more sensitive results, Bambu can be run with a less stringent NDR threshold. Without a reference annotation, Bambu cannot perform NDR calibration. Therefore, NDR threshold selection instead uses the undying prediction score and should be made much more sensitive than when reference annotations are provided to Bambu. When performing *de novo* transcript discovery, if there is a similar but well-annotated dataset for a related organism, it is possible to retrain the model used so that it is more applicable. **Funding:** A.S, Y.C, J.J.X.L, Y.K.W, and J.G are supported by funding from the Agency for Science, Technology, and Research (A*STAR) and the National Medical Research Council (NMRC).

Name: IsoQuant

Description: IsoQuant is a reference-based approach for transcript discovery and quantification using long RNA reads. Since version 3.0 it supports annotation-free transcript discovery. As version 2.0 did not support annotation-free transcript discovery, IsoQuant was launched using GTF obtained with StringTie2 (2.15).

Version: 2.0.0

Team: Center for Algorithmic Biotechnology, Saint Petersburg State University

URL: https://github.com/ablab/IsoQuant

Citations: Prjibelski, A.D., Mikheenko, A., Joglekar, A. *et al.* Accurate isoform discovery with IsoQuant using long reads. *Nat Biotechnol* (2023). https://doi.org/10.1038/s41587-022-01565-y

Config: --data_type nanopore for ONT data, --data_type pacbio_ccs for PacBio data. StringTie2 was launched using -L option.

Notes: IsoQuant can be installed via conda. Reads can be provided in BAM or in FASTQ format. In the latter case they will be automatically mapped using minimap2.

Funding: St. Petersburg State University (grant ID: 94030965), European Research Council (ERC) under the European Union's Horizon 2020 research and innovation programme (grant agreement No. 851093, SAFEBIO)

Name: RNA-Bloom

Description: RNA-Bloom uses a reference-free approach to assemble long transcriptomics reads.
Version: 1.4.3
Team: Birol Lab, University of British Columbia, Vancouver
URL: https://github.com/bcgsc/RNA-Bloom
Citations: Nip, K.M., Hafezqorani, S., Gagalova, K.K. *et al.* Reference-free assembly of long-read transcriptome sequencing data with RNA-Bloom2. *Nat Commun* **14**, 2940 (2023). https://doi.org/10.1038/s41467-023-38553-y
Config: For long + short reads (ONT + Illumina): `-t 48 -ntcard -artifact -long fulllength.fastq rescued.fastq unclassified.porechop.fastq -sef paired_1.fastq paired_2.fastq unpaired_1.fastq unpaired_2.fastq -fpr 0.005 -indel 20 -p 0.75 -Q 15 -overlap 100 -length 150`. For long reads only (ONT): `-t 48 -ntcard -artifact -long fulllength.fastq rescued.fastq unclassified.porechop.fastq -fpr 0.005 -indel 20 -p 0.75 -Q 15 -overlap 100 -length 150`. For short reads only (Illumina), `-t 48 -ntcard -stranded -left paired_1.fastq -right paired_2.fastq -rcr -sef unpaired_1.fastq -ser unpaired_2.fastq -fpr 0.005 -k 25 -indel 2 -q 15 -Q 15 -length 150`.
Notes: All tools and resources used to generate the assemblies are documented at https://github.com/bcgsc/lrgasp_birol
Funding: The development of RNA-Bloom was supported by Genome Canada and Genome British Columbia (243FOR); the National Institutes of Health (2R01HG007182-04A1); the Natural Sciences and Engineering Research Council of Canada (NSERC); and the Canadian Institutes of Health Research (CIHR).

Name: rnaSPAdes
Description: RnaSPAdes is a part of SPAdes package — a toolkit for various sequence assembly pipelines. RnaSPAdes is a transcriptome assembler preliminary designed for Illumina data but can handle long-read data as supplementary information.
Version: 3.15.3
Team: Pevzner Lab, St. Petersburg Academic University
URL: https://github.com/ablab/spades
Citations: Prjibelski, A., Antipov, D., Meleshko, D., Lapidus, A., & Korobeynikov, A. (2020). Using SPAdes de novo assembler. Current Protocols in Bioinformatics, 70, e102. https://doi.org/10.1002/cpbi.102
Config: (indicate if any special commands for specific library types)
Notes: Sequencing data was provided using the appropriate options from the user manual. Strand specificity was indicated using --ss rf flag.
Funding: St. Petersburg State University (grant ID: 94030965).

Method changes from registered report phase 1

While the great majority of the analyses indicated in the Registered Report are present in the final version of the manuscripts, some modifications were introduced. These are listed in the **Supplementary Table 13**.

Supplementary Tables

Supplementary Table 1: Overview of LRGASP sequencing data

Sample	# of Reps	cDNA-PacBio	cDNA-ONT	dRNA-ONT	R2C2-ONT	CapTrap-PacBio	CapTrap-ONT	cDNA-Illumina
Mouse ES	3	Yes	Yes	Yes	Yes	Yes	Yes	Yes
Human WTC11	3	Yes	Yes	Yes	Yes	Yes	Yes	Yes
Human H1-mix	3	Yes	Yes	Yes	Yes	Yes	Yes	Yes
Human H1-hESC	*3*	Yes	Yes	Yes	Yes	Yes	Yes	Yes
Human H1-DE	*3*	Yes	Yes	Yes	Yes	Yes	Yes	Yes
Manatee leukocytes	1	Yes	Yes	No	No	No	No	Yes

Supplementary Table 2: Summary statistics for ES sequencing data

Sample	ES					
Method	dRNA	cDNA	R2C2	CapTrap	CapTrap	cDNA
Tech	ONT	ONT	ONT	ONT	PacBio	PacBio
Platform	MinION	MinION	MinION	MinION	SequelII	SequelII
# of Flowcells/SMRT cells	3	3	6	3	3	9
# of raw reads	4,325,200	59,746,818	7,862,883[1]	56,684,765	9,689,619	23,487,808
# of supplied reads	**3,975,725**	**57,055,583**	**5,930,487**	**50,697,997**	**5,090,848**	**8,733,814**
# of aligned reads	3,836,020	44,873,564	5,914,779	49,741,194	5,028,403	8,199,908
# of aligned reads with adapters	N/A	40,190,805	5,914,779	32,206,495	5,028,403	8,199,908
Median Read length	830	519	1,755	591	903	2,090
Median Identity (Q score)	9.8	12.7	18.6	12.3	21.3	20.9
% Directionality	99.54	98.59	99.74	94.66	99.88	99.55
% of spike-in reads	0.71	1.02	2.03	2.41	1.77	1.85
Pearson r2 (gene level)	0.99	0.99	0.98	0.99	0.98	0.97

For each sample, replicates were combined when reporting statistics.
[1]R2C2 libraries for ES and WTC11 libraries were multiplexed, and raw reads cannot be demultiplexed directly. Raw read numbers for these libraries are therefore calculated based on the ES/WTC11 ratio of demultiplexed supplied consensus reads and the total number of subreads.

Supplementary Table 3: Error rates in percentage for real and simulated data of different types obtained via read alignment

Data type	Error type	Real data	Simulated
cDNA-PacBio	Mismatches	0.25	0.46
	Insertions	0.57	0.57
	Deletions	0.45	0.64
	Total	1.27	1.67
cDNA-ONT	Mismatches	2.5	4.2
	Insertions	3.3	5.1
	Deletions	1.6	4.1
	Total	7.4	13.4
dRNA-ONT	Mismatches	7.0	6.0
	Insertions	5.2	5.4
	Deletions	2.9	2.1
	Total	15.1	13.5

Supplementary Table 4: Summary statistics for WTC11 sequencing data

Sample	WTC11					
Method	dRNA	cDNA	R2C2	CapTrap	CapTrap	cDNA
Tech	ONT	ONT	ONT	ONT	PacBio	PacBio
Platform	MinION	MinION	MinION	MinION	SequelII	SequelII
# of Flowcells/SMRT cells	3	3	6	3	3	9
# of raw reads	3,229,571	53,463,774	6,994,789[1]	56,730,485	13,463,712	28,567,150
# of supplied reads	**2,988,430**	**51,194,535**	**5,275,737**	**50,902,303**	**6,399,632**	**7,424,923**
# of aligned reads	2,931,482	43,085,527	5,271,334	49,930,350	6,304,610	7,373,147
# of aligned reads with adapters	N/A	37,275,068	5,271,334	31,348,191	6,304,610	7,373,147
Median Read length	854	610	1,802	564	864	2,209
Median Identity (Q score)	9.8	12.9	19.3	12.9	22.5	23.8
% Directionality	99.76	99.11	99.92	96.28	99.92	99.67
% of spike-in reads	0.6	1.45	2.27	2.79	2.26	2.25
Pearson r2 (gene level)	0.92	0.96	0.94	0.99	0.96	0.90

For each sample, replicates were combined when reporting statistics.
[1]R2C2 libraries for ES and WTC11 libraries were multiplexed, and raw reads cannot be demultiplexed directly. Raw read numbers for these libraries are therefore calculated based on the ES/WTC11 ratio of demultiplexed supplied consensus reads and the total number of subreads.

Supplementary Table 5: Summary statistics for H1-mix sequencing data

Sample	H1-mix					
Method	dRNA	cDNA	R2C2	CapTrap	CapTrap	cDNA
Tech	ONT	ONT	ONT	ONT	PacBio	PacBio
Platform	MinION	MinION	MinION	MinION	SequelII	SequelII
# of Flowcells/SMRT cells	3	3	6	3	3	6
# raw reads	4,223,164	55,927,828	7,093,671	54,055,468	10,534,880	24,290,762
# of supplied reads	**3,969,603**	**52,927,595**	**5,231,255**	**49,883,469**	**5,511,853**	**5,511,357**
# of aligned reads	3,905,742	43,026,016	5,229,686	48,424,901	5,436,170	5,480,635
# of aligned reads with adapters	N/A	36,653,422	5,229,686	28,099,080	5,436,170	5,480,635
Median Read length	891	619	1,782	604	1,036	2,376
Median Identity (Q score)	10.0	12	18.7	12.4	24.3	23.7
% Directionality	99.8	99.19	99.74	**76.15**[1]	99.91	99.63
% of spike-in reads	0.77	1.5	1.69	1.59	1.33	1.97
Pearson r2 (gene-level)	0.99	0.997	0.98	0.96	0.98	0.98

[1]Replicate 3 of the H1_mix sample appears to be an outlier among the CapTrap ONT library type. Replicates 1 and 2 show % directionality that is ~95% similar to what is observed in the other samples for this library type.

Supplementary Table 6: Summary statistics for Manatee sequencing data

Sample	Manatee	Manatee
Method	cDNA	cDNA
Tech	ONT	PacBio
Platform	MinION	Sequel I + Sequel II
# of Flowcells/SMRT cells	3	1+3
# of supplied reads	**40,948,571**	**6,883,684**
# of aligned reads	32,833,840	6,877,181
# of aligned reads with adapters	27,381,394	6,877,181
Median Read length	540	894
Median Accuracy (Q score)	12.5	25.2
% Directionality	97.2	99.76
% of spike-in reads	**14.05***	**33.78***
*spike-in percentage is higher than expected		

Supplementary Table 7: LRGASP participation summary

Tool	Submitter	Challenge Participation			Library Preparation				Sequencing Platforms					
		1	2	3	Cap Trap	R2C 2	cDN A	dRN A	ONT	PB	ONT + short	PB + short	ONT + PB	cDNA Illumina
Bambu	Göke, Genome Institute of Singapore	✔	✔	✔	✔	✔	✔	✔	✔	✔				
FLAIR	Brooks Lab, University of California, Santa Cruz	✔	✔				✔	✔	✔	✔	✔	✔		
LyRiC	Guigó Lab, Centre de Regulació Genòmica	✔			✔	✔	✔	✔	✔	✔				
IsoTools	Herwig Lab, Max Planck Institute for Molecular Genetics	✔	✔				✔			✔				
StringTie2	Pertea Lab, Johns Hopkins University	✔						✔			✔			
Spectra	Hideya Kawaji, Tokyo Metropolitan Institute of Medical Science	✔					✔			✔				
TALON	Mortazavi Lab, University of California, Irvine	✔	✔		✔	✔	✔	✔	✔	✔	✔	✔		
ISO_IB	Integrative Bioinformatics, NIEHS	✔					✔			✔				
FLAMES	Ritchie Lab, The Walter and Eliza Hall Institute	✔	✔		✔		✔	✔	✔	✔				
IsoQuant	Center for Algorithmic Biotechnology, Saint Petersburg State University	✔	✔	✔	✔	✔	✔	✔	✔	✔				
Mandalorion	Vollmers Lab, University of California, Santa Cruz	✔				✔	✔		✔	✔			✔	
NanoSim	Birol Lab, University of British Columbia, Vancouver		✔				✔	✔	✔					
RNA-Bloom	Birol Lab, University of British Columbia, Vancouver			✔			✔		✔		✔			
rnaSPAdes	Pevzner Lab, St. Petersburg Academic University			✔			✔	✔			✔	✔		✔

Supplementary Table 8: Metrics for evaluation against GENCODE annotation

Metric	FSM	ISM	NIC	NNC	Others
Count	✓	✓	✓	✓	✓
Reference Match (RM)*	✓				
_3′ poly(A) supported	✓	✓	✓	✓	
_5′ CAGE supported	✓	✓	✓	✓	
_3′ reference supported	✓	✓	✓	✓	
_5′ reference supported	✓	✓	✓	✓	
Supported Reference Transcript Model (SRTM)	✓	✓			
Supported Novel Transcript Model (SNTM)			✓	✓	
Distance (nts) to TSS/TTS of matched transcript	✓	✓			
Redundancy	✓	✓			
% Long Read Coverage (%LRC)	✓				
Longest Junction Chain		✓	✓	✓	
Intron retention level		✓	✓		
Illumina Splice Junction Support	✓	✓	✓	✓	✓
Full Illumina Splice Junction Support	✓	✓	✓	✓	✓
% Novel Junctions			✓	✓	
% Non-canonical junctions	✓	✓	✓	✓	✓
% Transcripts with non-canonical junctions	✓	✓	✓	✓	✓
Intra-priming	✓	✓	✓	✓	✓
RT-switching	✓	✓	✓	✓	✓
Number of exons	✓	✓	✓	✓	✓

* See **Box 1** for the description of LRGASP metrics

✓ *indicates the LRGASP metric in the row is applied to the structural category in the column*

Supplementary Table 9: Metrics and definitions for evaluation against SIRVs

Metric	Description
Reference SIRV (rSIRV)	Ground truth SIRV model
SIRV_transcripts	Transcripts mapping to a SIRV chromosome
SIRV_RM	SIRV_transcripts matching a rSIRV as Reference Match
True Positive detections (TP)	rSIRVs identified as RM
Partial True Positive detections (PTP)	rSIRVs identified as ISM or FSM_non_RM
False Negative (FN)	rSIRVs without FSM or ISM
False Positive (FP)	NIC + NNC + antisense + fusion SIRV_transcripts
Sensitivity	TP/rSIRVs
Precision	RM/SIRV_transcripts
Non_redundant Precision	TP/SIRV_transcripts
Positive Detection Rate	unique(TP+PTP)/rSIRVs
False Discovery Rate	(SIRV_transcripts - SIRV_RM)/SIRV_transcripts
False Detection Rate	FP/SIRV_transcripts
Redundancy	(FSM + ISM)/unique(TP+PTP)

Supplementary Table 10: Metrics and definitions for evaluation against simulated data

Metric	Description
True Positive (TP)<br>TP_ref<br>TP_novel	RM<br>RM to GENCODE mdels<br>RM to simulated novel transcript models
Partial True Positive (PTP)<br>PTP_ref<br>PTP_novel	ISM or FSM_non_RM<br>ISM or FSM_non_RM of GENCODE models<br>ISM or FSM_non_RM of simulated novel models
False Negative (FN)<br>FN_ref<br>FN_novel	Simulated transcripts without RM or PTP calls<br>Simulated GENCODE models without RM or PTP calls<br>Simulated novel models without RM or PTP calls
False Positive (FP)	NIC + NNC + antisense + fusion
Sensitivity<br>Sens_ref<br>Sens_novel	 TP_ref/P(GENCODE)<br>TP_novel/P(Simulated novel)
Precision	TP/(TP+PTP+FP)
Positive Detection Rate	(TP+PTP)/P
False Discovery Rate	(FP+PTP)/(TP+PTP+FP)
False Detection Rate	FP/(TP+PTP+FP)
Redundancy	# FSM and ISM per simulated transcript model

Supplementary Table 11: Metrics for evaluation of manually annotated transcript models

Metric	Description
TP	RM
PTP	ISM or FSM_not_RM
FN	Curated GENCODE transcripts without FSM or ISM
Sensitivity	TP_ref/Curated GENCODE transcripts
Positive Detection Rate	(TP+PTP)/Curated GENCODE transcripts
Redundancy	(FSM + ISM)/unique(TP+PTP)

Supplementary Table 12: WTC-11 Validation Batches

Amplicon Pool	Pooled Amplicon Concentration (ng/uL)	Total Volume Extracted From Amplicons in Pool (uL)	Total Amount of DNA (ng) in Pools
Batch_1 WTC 'POOL'	71	51	3,621
Batch_2 WTC_Rep_2	79.4	144	11,433.60
Batch_2 WTC_Rep_3	70.2	144	10,108.80
Batch_3 WTC_Rep_2	47.2	132	6,230.40
Batch_3 WTC_Rep_3	73.8	132	9,741.60
Batch_4 WTC_Rep_2	20.8	243	5,054.40
Batch_4 WTC_Rep_3	64.4	243	15,649.20

Supplementary Table 13: Method Changes

Modification	Section	Description
Orthogonal data	Introduction	Orthogonal data for human was Illumina, CAGE, and Quant-seq. We did not generate or use ChIP-seq or ATAC-seq
Analysis of novel gene transcripts	Challenge 1	Exhaustive analysis of transcripts in novel genes and SQANTI categories other than FSM, ISM, NIC, NNC was not performed, although these numbers are included in these supplementary tables.
%LRC analysis	Challenge 1	Fraction of the transcript model sequence length mapped by one or more long reads (%LRC) analysis for GENCODE annotated transcripts was not performed as GENCODE transcripts were called de novo by annotators rather than validated from submissions to avoid biases.
Percentage of Expressed Transcripts (PET)	Challenge 2	A newly added metric is used to characterize the percentage of truly expressed transcripts in SIRV-set4 data.
Abundance Recovery Rate (ARR) skipped	Challenge 2	Considering the redundancy of multiple evaluation metrics, the ARR metric was skipped.
ROC-based metrics skipped	Challenge 2	Current real data cannot obtain the truly differentially expressed transcripts (i.e., the ground truth) due to a lack of qPCR validation. So, ROC-based metrics were skipped.
Assessment without a reference genome	Challenge 3	This assessment was not launched, and Challenge 3 was restricted to transcript identification without a reference annotation but with a reference genome. This turned out to be quite challenging already.
Number of transcripts/loci	Challenge 3	This information was not asked to submitters, as initially planned, but computed during the analysis
Blast2GO analysis skipped	Challenge 3	Functional annotation of transcript predictions was skipped due to long computing times. BUSCO analysis is a proxy for this analysis
Validation of TM with manatee 454 data skipped	Challenge 3	We found limitations when accessing the data.
Validation of	Validation	We could not find a tractable and cost-manageable

Modification	Section	Description
quantitative levels of isoforms for Challenge 2		technique for providing reliable isoform quantity estimates. Isoform-specific qPCR was cost-prohibitive and, for the targets of interest, not amenable to analysis.
Target selection for Challenge 1.	Validation	We added additional test categories including novel and suspect transcripts from the GENCODE manual annotation. We report data for the human WTC11 sample. No mouse targets were validated due to insufficient material and resources.

References

1. Hafezqorani, S. *et al.* Trans-NanoSim characterizes and simulates nanopore RNA-sequencing data. *Gigascience* **9**, (2020).
2. Li, W. *cdhit: Automatically exported from code.google.com/p/cdhit*. (Github).
3. Tian, L. *et al.* Comprehensive characterization of single-cell full-length isoforms in human and mouse with long-read sequencing. *Genome Biol.* **22**, 310 (2021).
4. Prjibelski, A. D. *et al.* Accurate isoform discovery with IsoQuant using long reads. *Nat. Biotechnol.* **41**, 915–918 (2023).
5. Carbonell-Sala, S. *et al.* CapTrap-Seq: A platform-agnostic and quantitative approach for high-fidelity full-length RNA transcript sequencing. *bioRxiv* (2023) doi:10.1101/2023.06.16.543444.
6. Kolmogorov, M., Yuan, J., Lin, Y. & Pevzner, P. A. Assembly of long, error-prone reads using repeat graphs. *Nat. Biotechnol.* **37**, 540–546 (2019).
7. Raney, B. J. *et al.* Track data hubs enable visualization of user-defined genome-wide annotations on the UCSC Genome Browser. *Bioinformatics* **30**, 1003–1005 (2014).
8. Nassar, L. R. *et al.* The UCSC Genome Browser database: 2023 update. *Nucleic Acids Res.* **51**, D1188–D1195 (2023).
9. Untergasser, A. *et al.* Primer3—new capabilities and interfaces. *Nucleic Acids Res.* **40**, e115–e115 (2012).
10. In-Silico PCR. *UCSC Genome Bioinformatics* http://genome.ucsc.edu/.

Supplementary Figures

The following pages contain **Supplementary Figs. 1-79**.

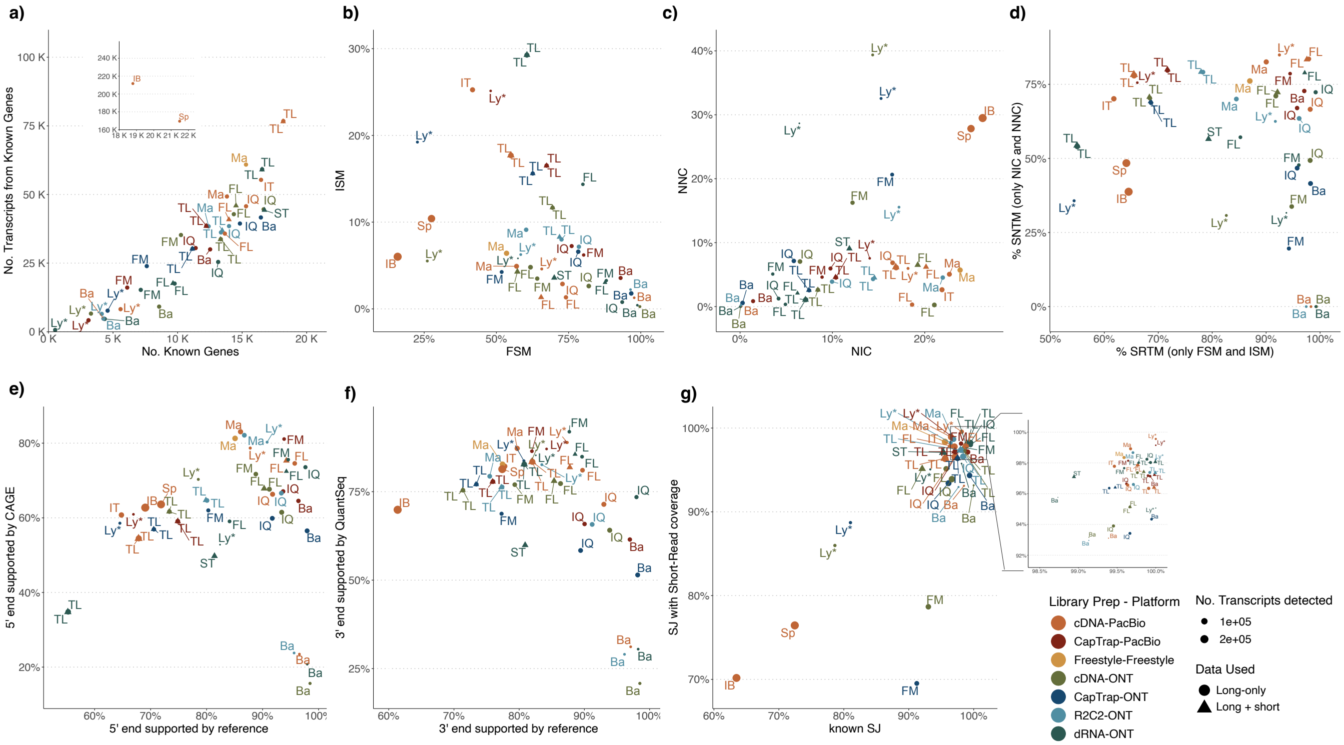

Supplementary Fig. 1. SQANTI3 evaluation of LRGASP submissions of the H1-mix dataset. Labels correspond to analysis tools and the color code indicates the combination of library preparation and sequencing platform. a) Number of gene and transcript detections. b) Number of Full Splice Match and Incomplete Splice Match transcripts. c) Number of Novel in Catalogue and Novel Not in Catalogue transcripts. d) Percentage of known and novel transcripts with full support at junctions and end positions. e) Percentage of transcripts with 5´end support. f) Percentage of transcripts with 3´end support. g) Percentage of canonical splice junctions (SJ) and short-reads support at SJ. Ba: Bambu, FM: Flames, FL: FLAIR, IQ: IsoQuant, IT: IsoTools, IB: Iso_IB, Ly: LyRic, Ma: Mandalorion, TL: TALON-LAPA, Sp: Spectra, ST: StringTie2.

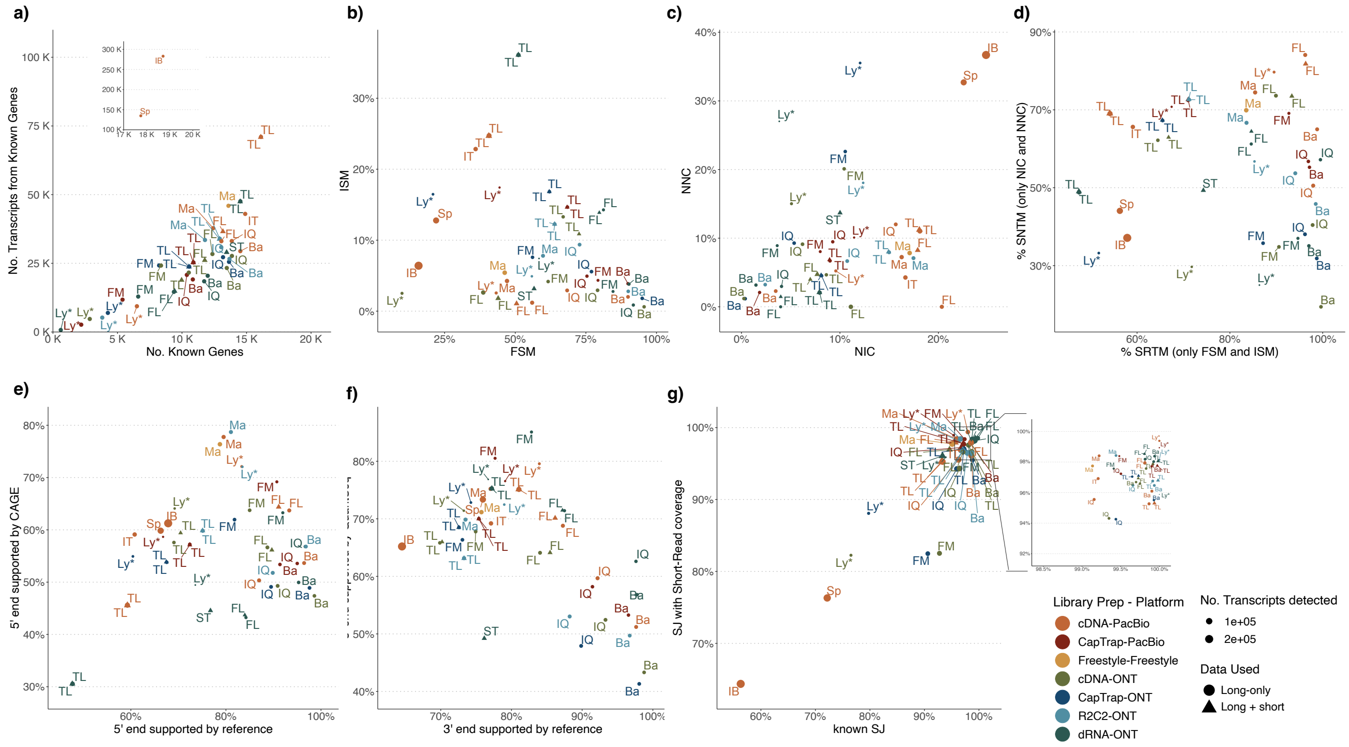

Supplementary Fig. 2. SQANTI3 evaluation of LRGASP submissions of the mouse ES dataset. Labels correspond to analysis tools and the color code indicates the combination of library preparation and sequencing platform. a) Number of gene and transcript detections. b) Number of Full Splice Match and Incomplete Splice Match transcripts. c) Number of Novel in Catalogue and Novel Not in Catalogue transcripts. d) Percentage of known and novel transcripts with full support at junctions and end positions. e) Percentage of transcripts with 5´end support. f) Percentage of transcripts with 3´end support. g) Percentage of canonical splice junctions (SJ) and short-reads support at SJ. Ba: Bambu, FM: Flames, FL: FLAIR, IQ: IsoQuant, IT: IsoTools, IB: Iso_IB, Ly: LyRic, Ma: Mandalorion, TL: TALON-LAPA, Sp: Spectra, ST: StringTie2.

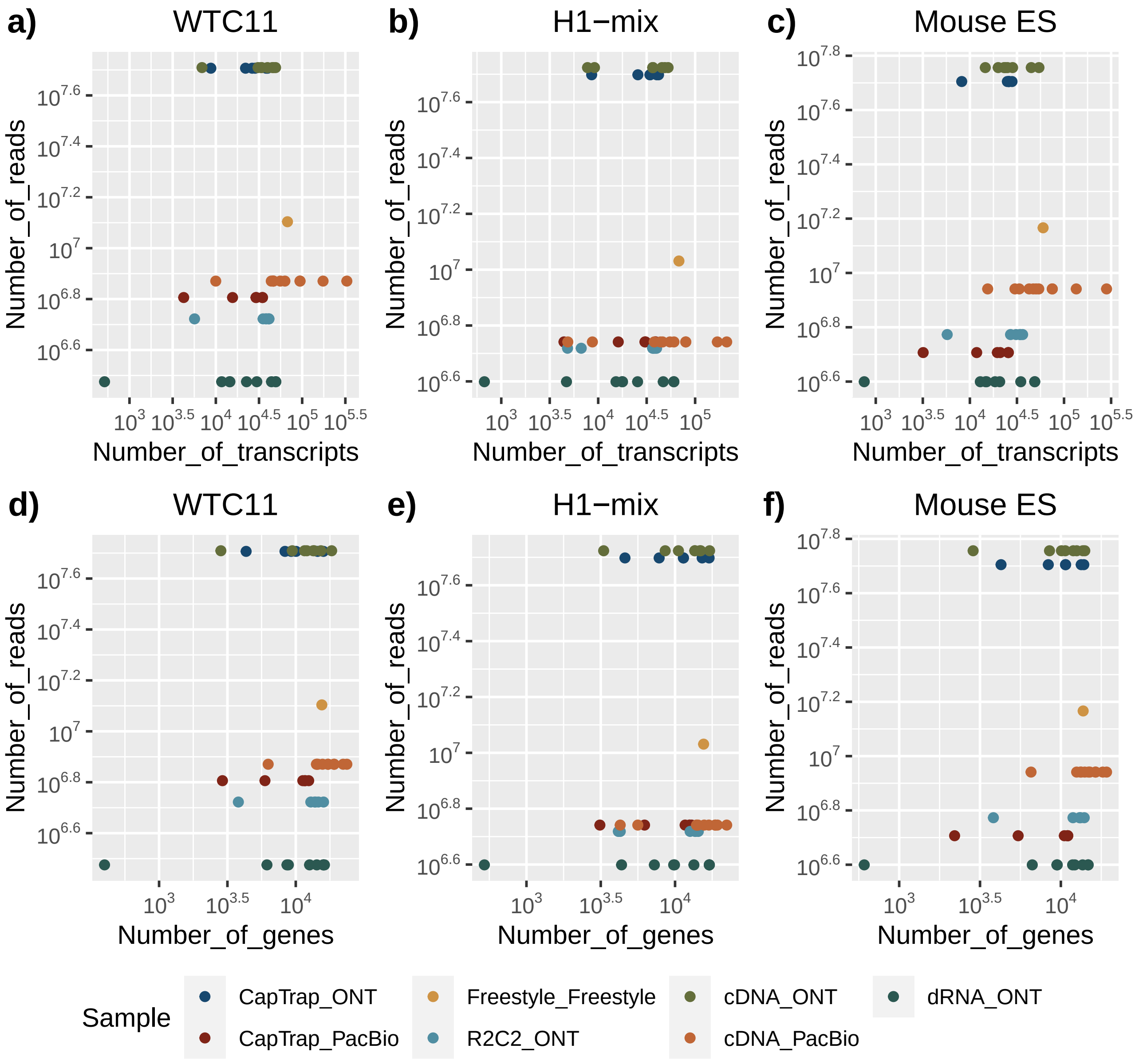

Supplementary Fig. 3. Relationship between sequencing depth and number of detected features. a-c) Transcripts, d-f) Genes.

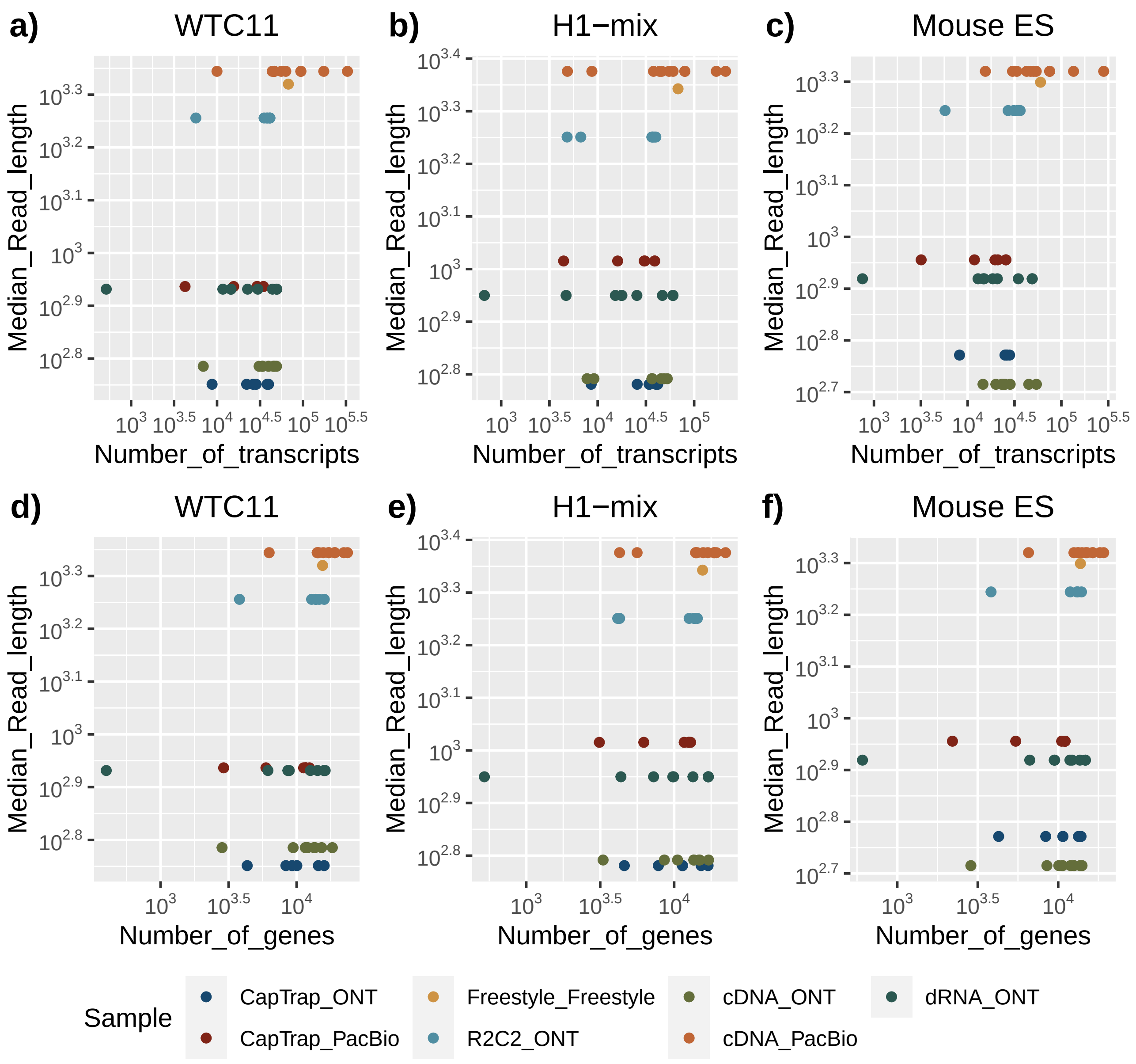

Supplementary Fig. 4. Relationship between read length and number of detected features. a-c) Transcripts, d-f) Genes.

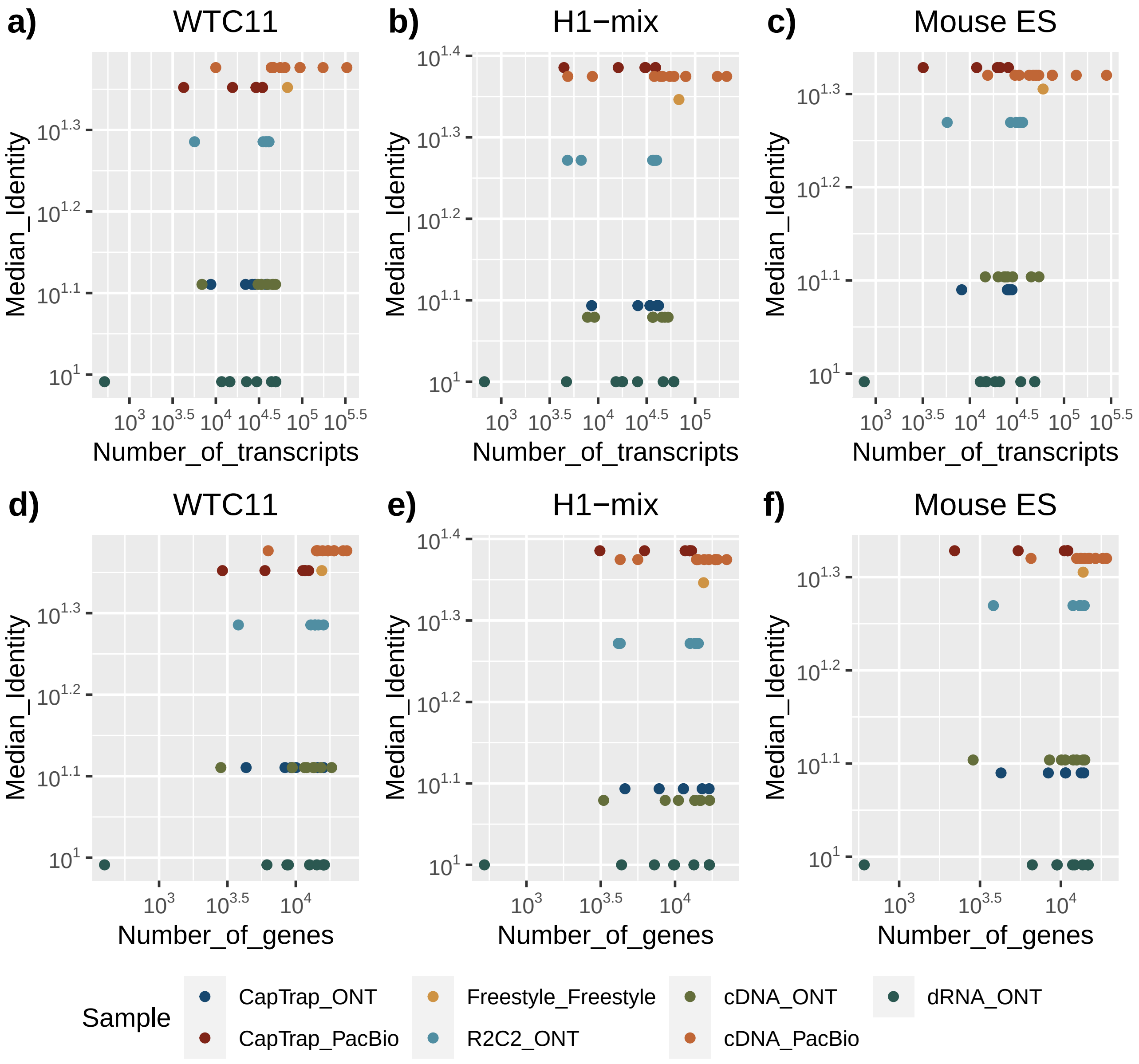

Supplementary Fig. 5. Relationship between read quality and number of detected features. a-c) Transcripts, d-f) Genes.

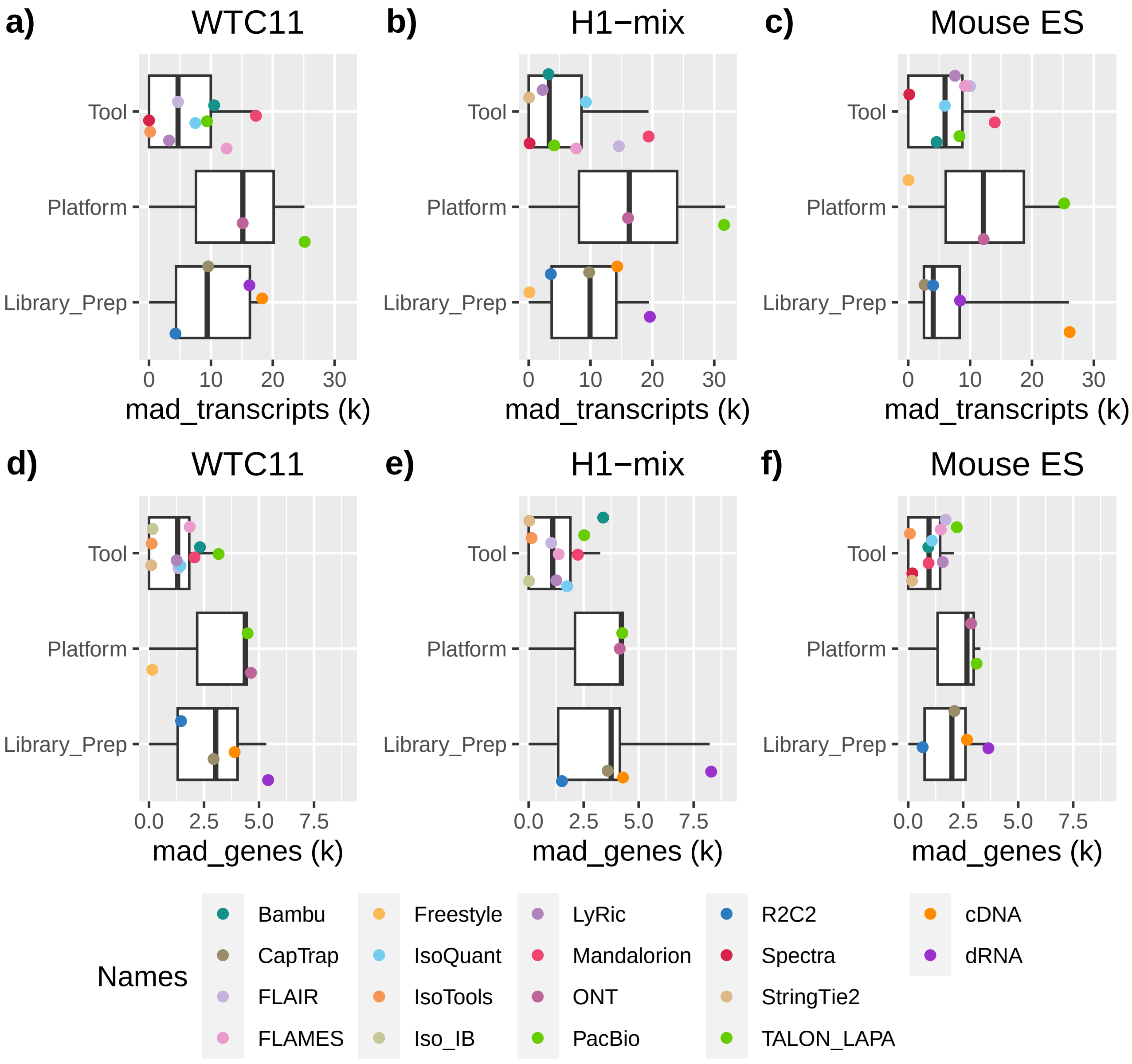

Supplementary Fig. 6. Median Absolute Deviance of detected features by experimental factor. a-c) Transcripts, d-f) Genes.

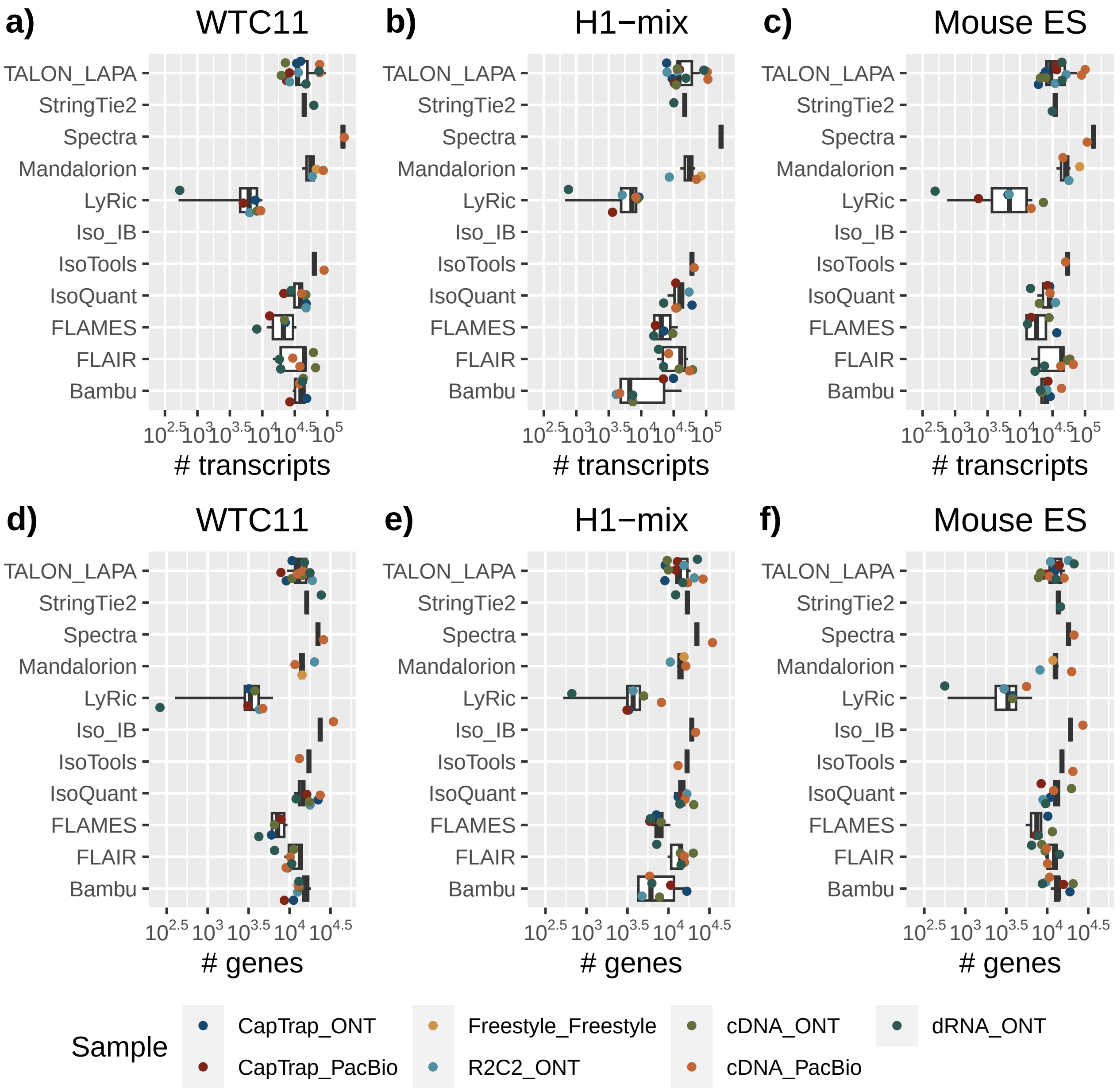

Supplementary Fig. 7. Number of detected transcripts and genes per analysis tool. a-c) Transcripts, d-f) Genes.

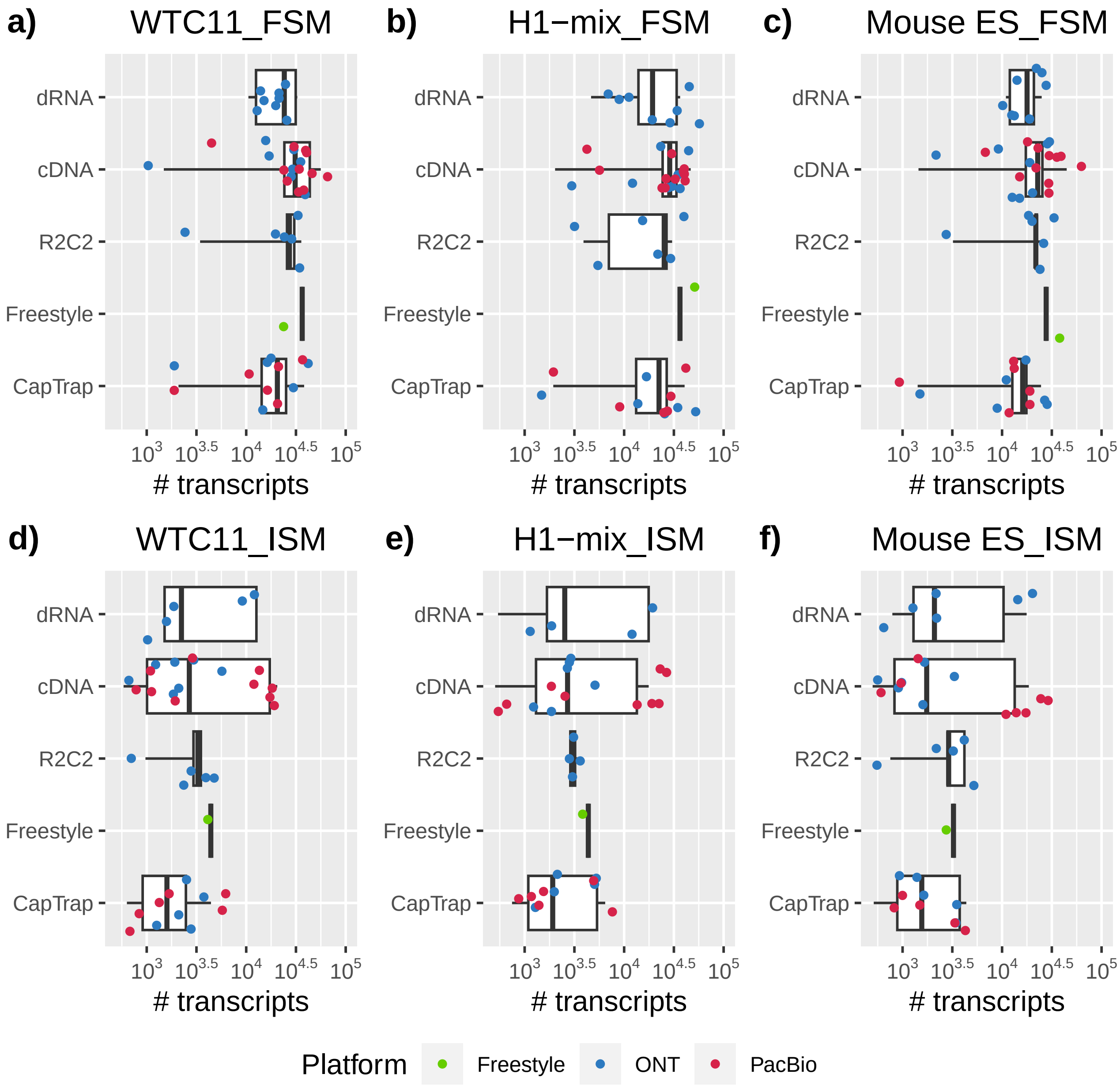

Supplementary Fig. 8. Number of FSM and ISM by sequencing platform and library preparation. a-c) FSM, d-f) ISM.

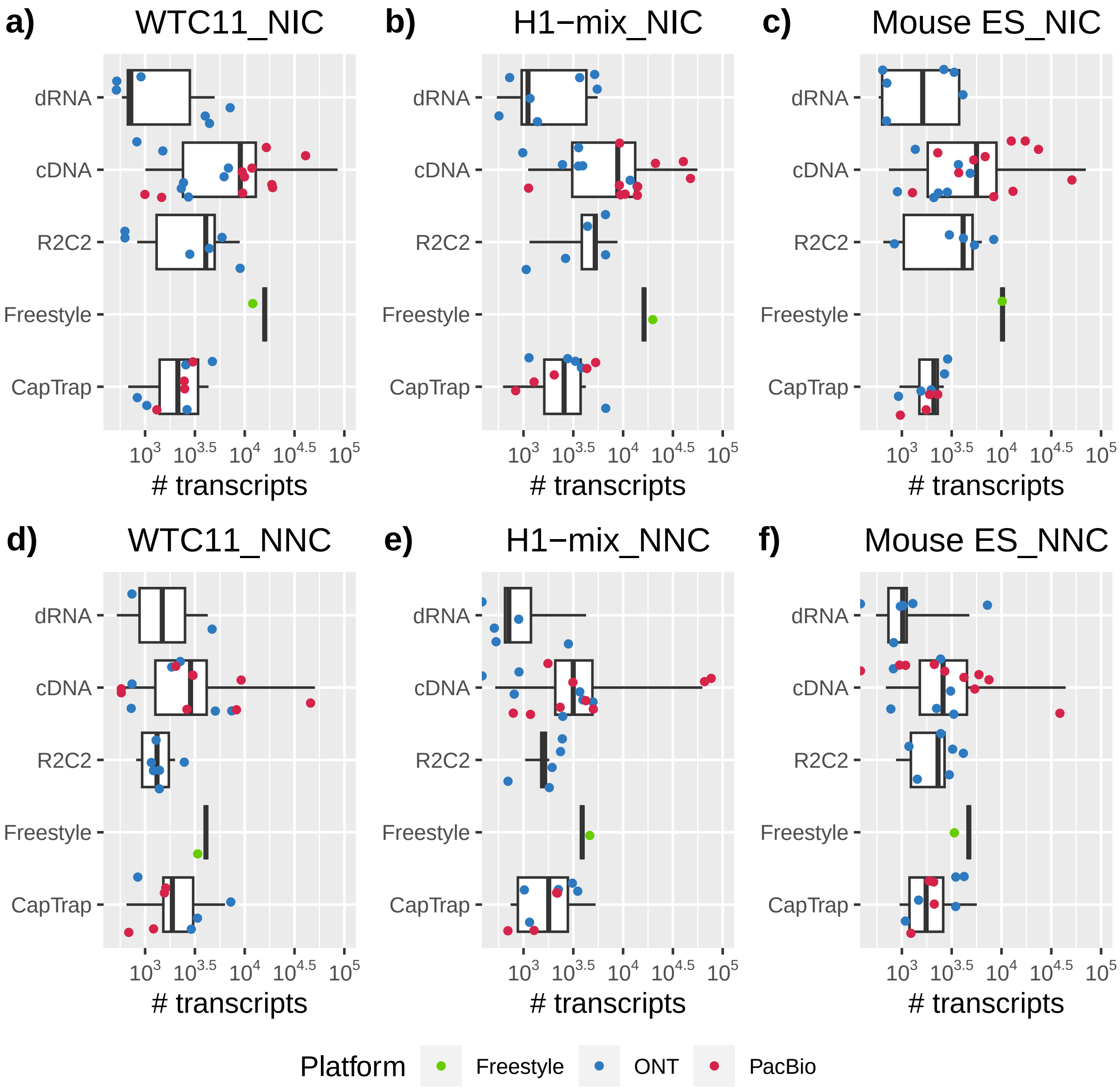

Supplementary Fig. 9. Number of NIC and NNC by sequencing platform and library preparation. a-c) NIC, d-f) NNC.

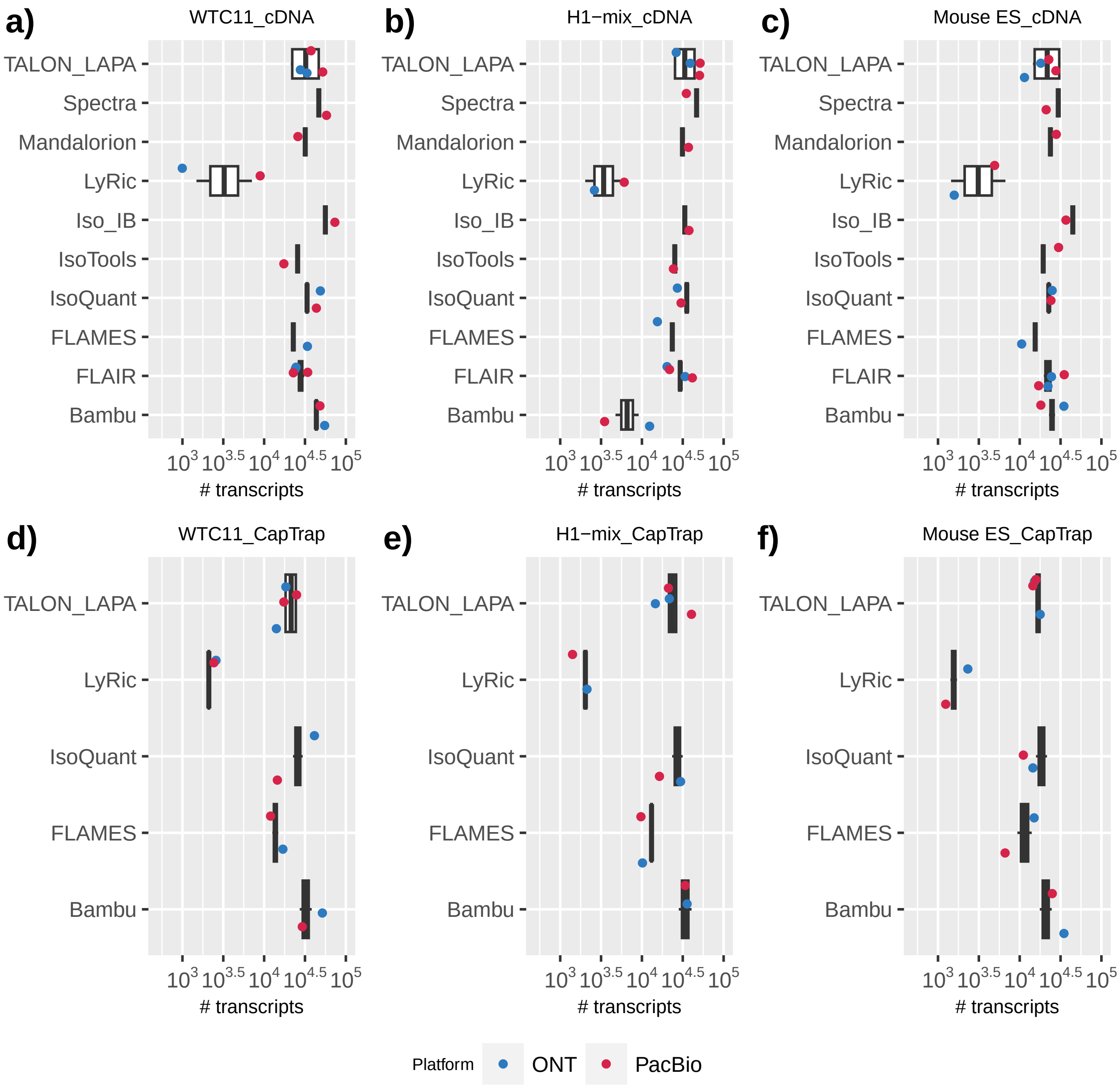

Supplementary Fig. 10. Number of FSM transcripts by library preparation and analysis tool. a-c) cDNA. d-f) CapTrap.

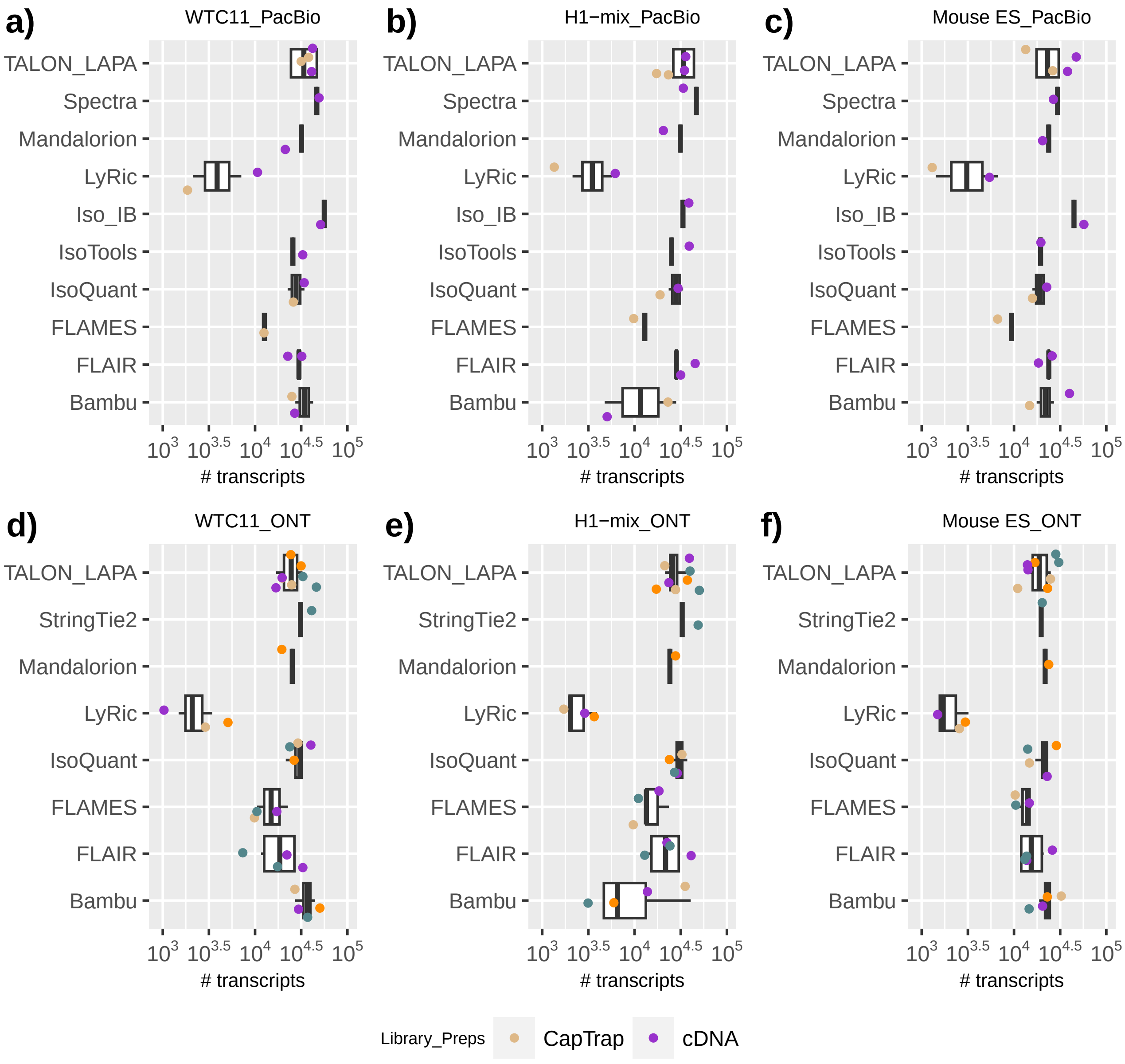

Supplementary Fig. 11. Number of FSM transcripts by sequencing platform and analysis tool. a-c) PacBio, d-f) Nanopore.

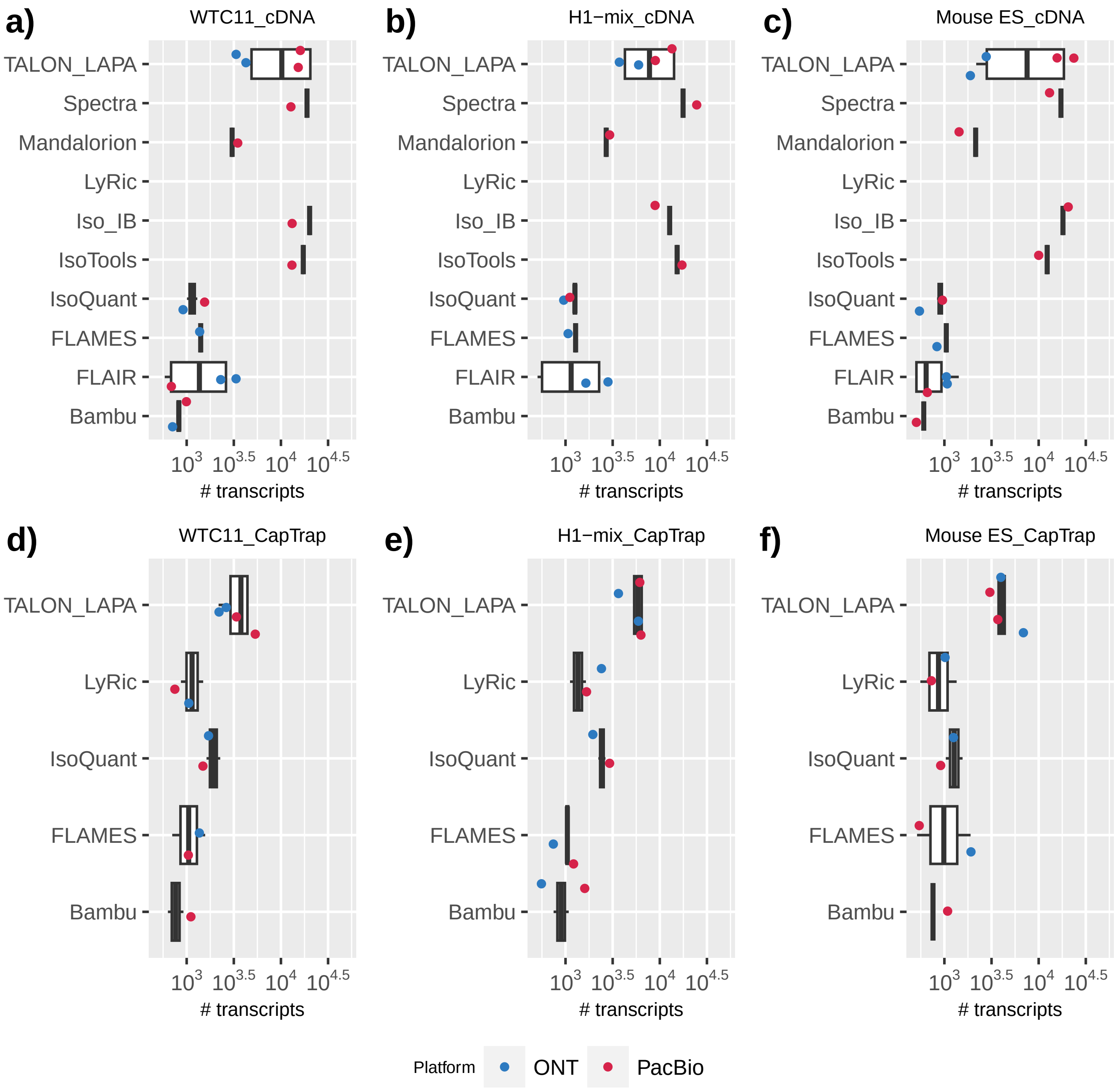

Supplementary Fig. 12. Number of ISM transcripts by library preparation and analysis tool. a-c) cDNA. d-f) CapTrap.

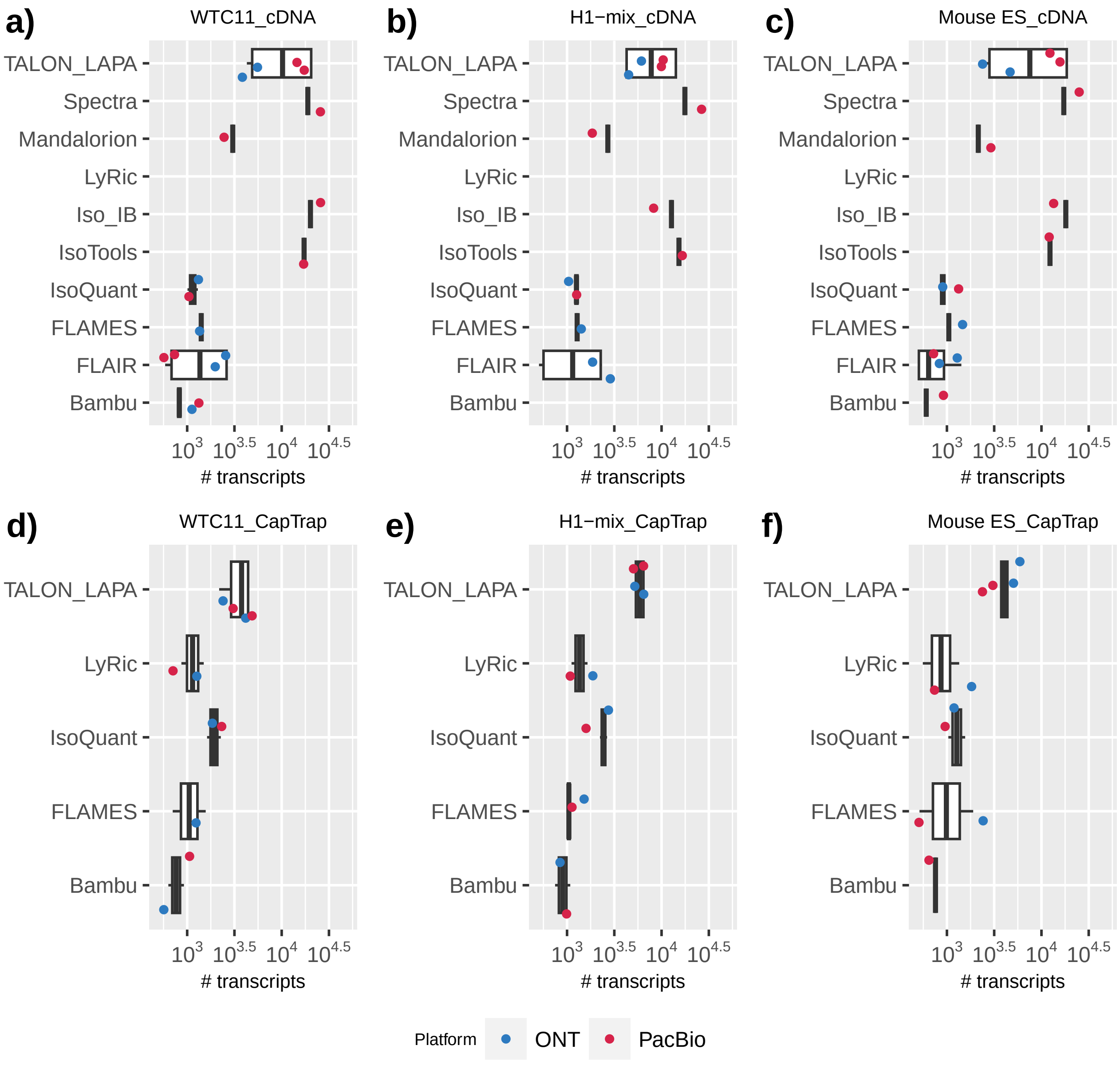

Supplementary Fig. 13. Number of ISM transcripts by sequencing platform and analysis tool. a-c) Intergenic. d-f) GenicGenomic.

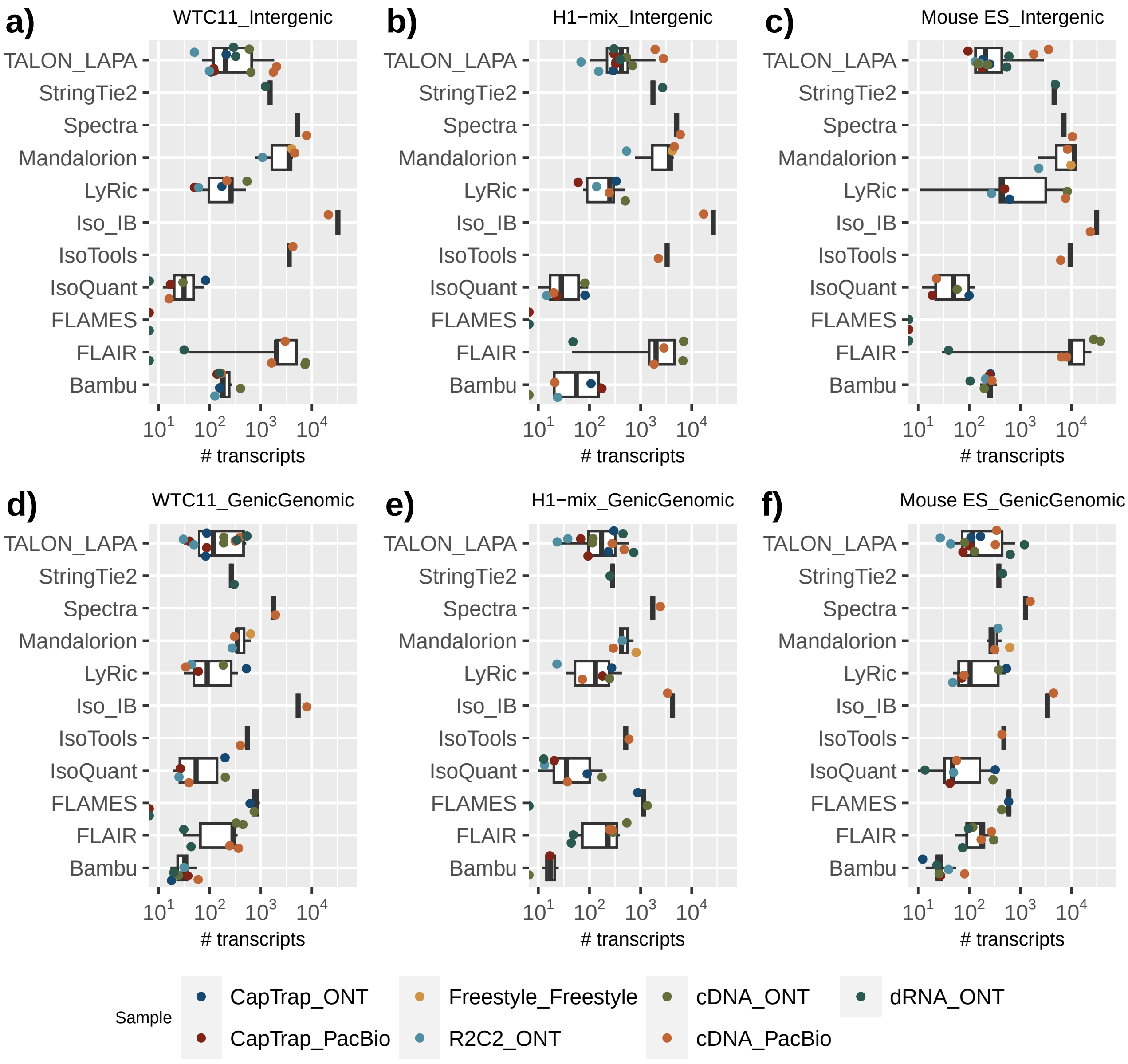

Supplementary Fig. 14. Number of Intergenic and GenicGenomic by sequencing platform and library preparation. a-c) Intergenic, d-f) GenicGenomic.

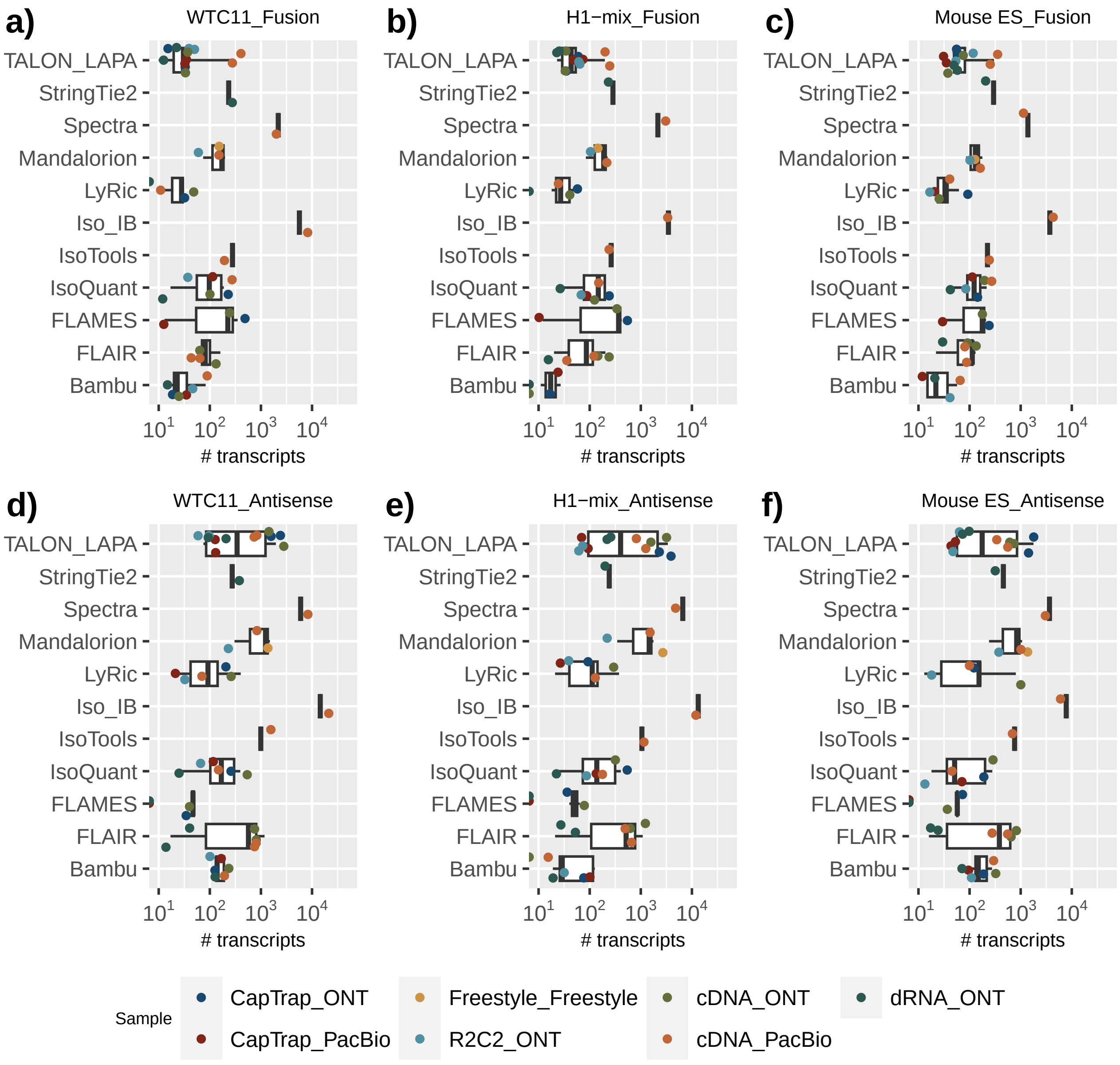

Supplementary Fig. 15. Number of Fusion and Antisense by sequencing platform and library preparation. a-c) Fusion. d-f) Antisense.

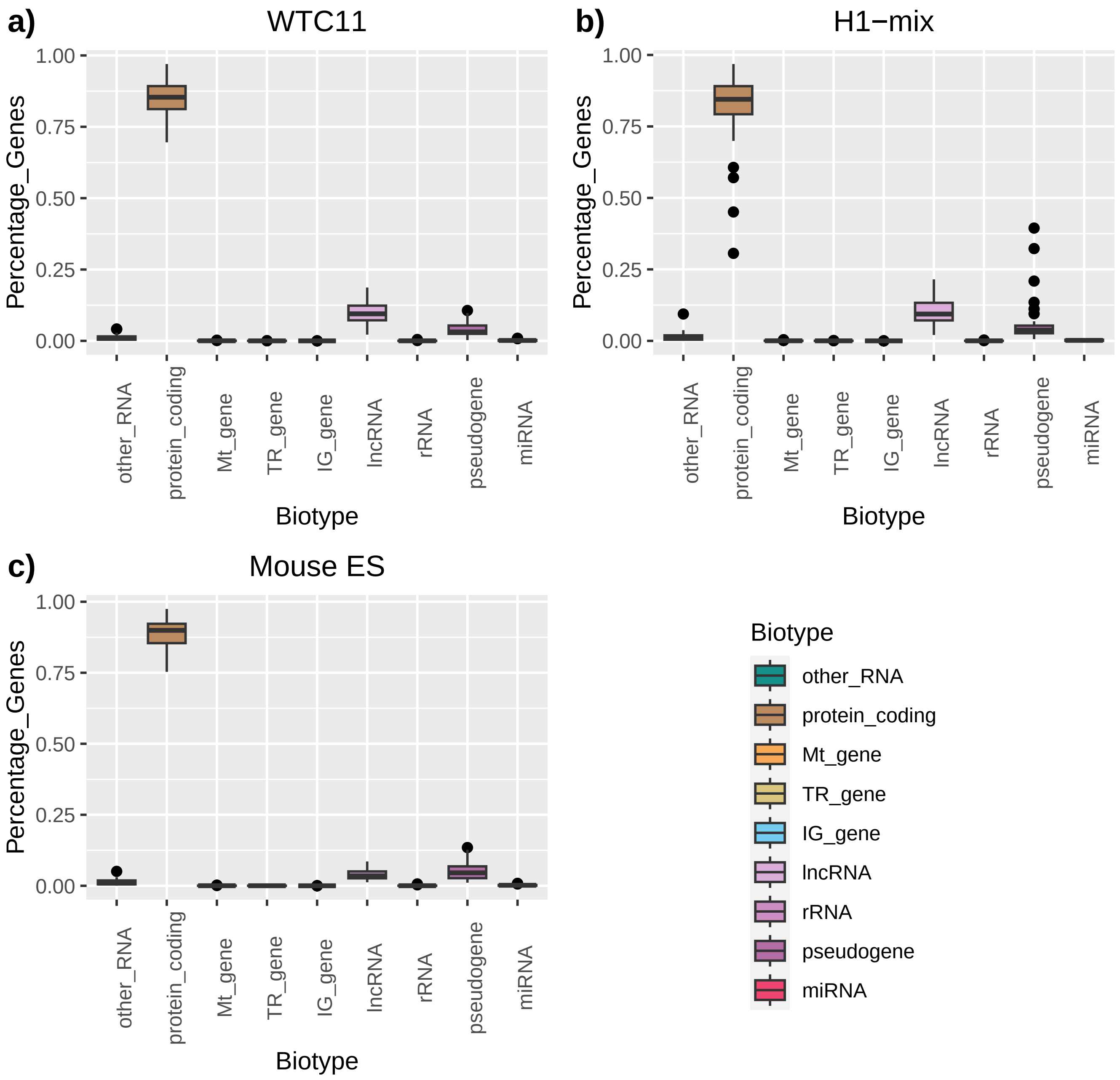

Supplementary Fig. 16. Distribution of Biotypes across samples. a) WTC11, c) H1-mix, c) Mouse ES.

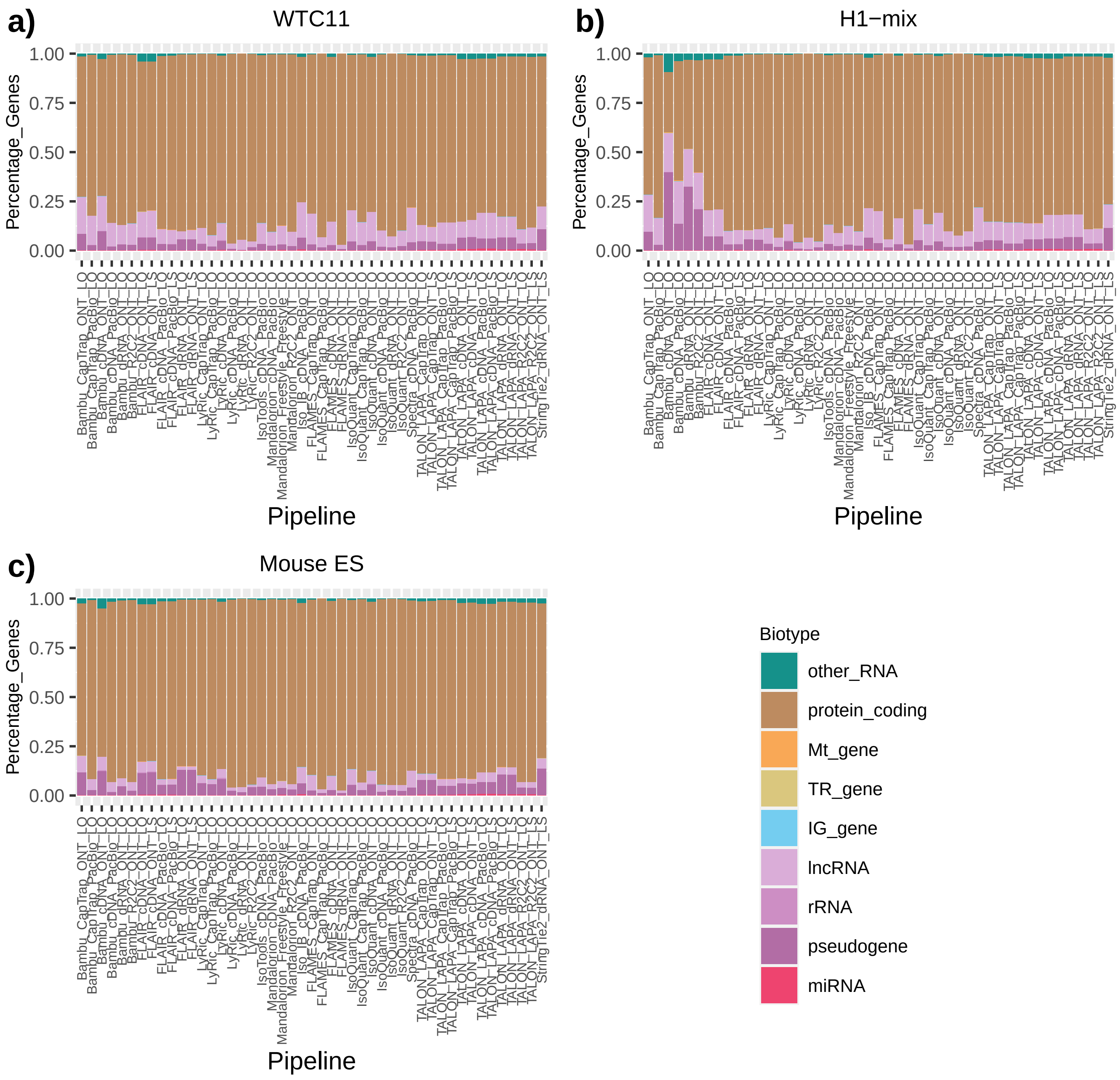

Supplementary Fig. 17. Distribution of Biotypes across pipelines. a) WTC11, c) H1-mix, c) Mouse ES.

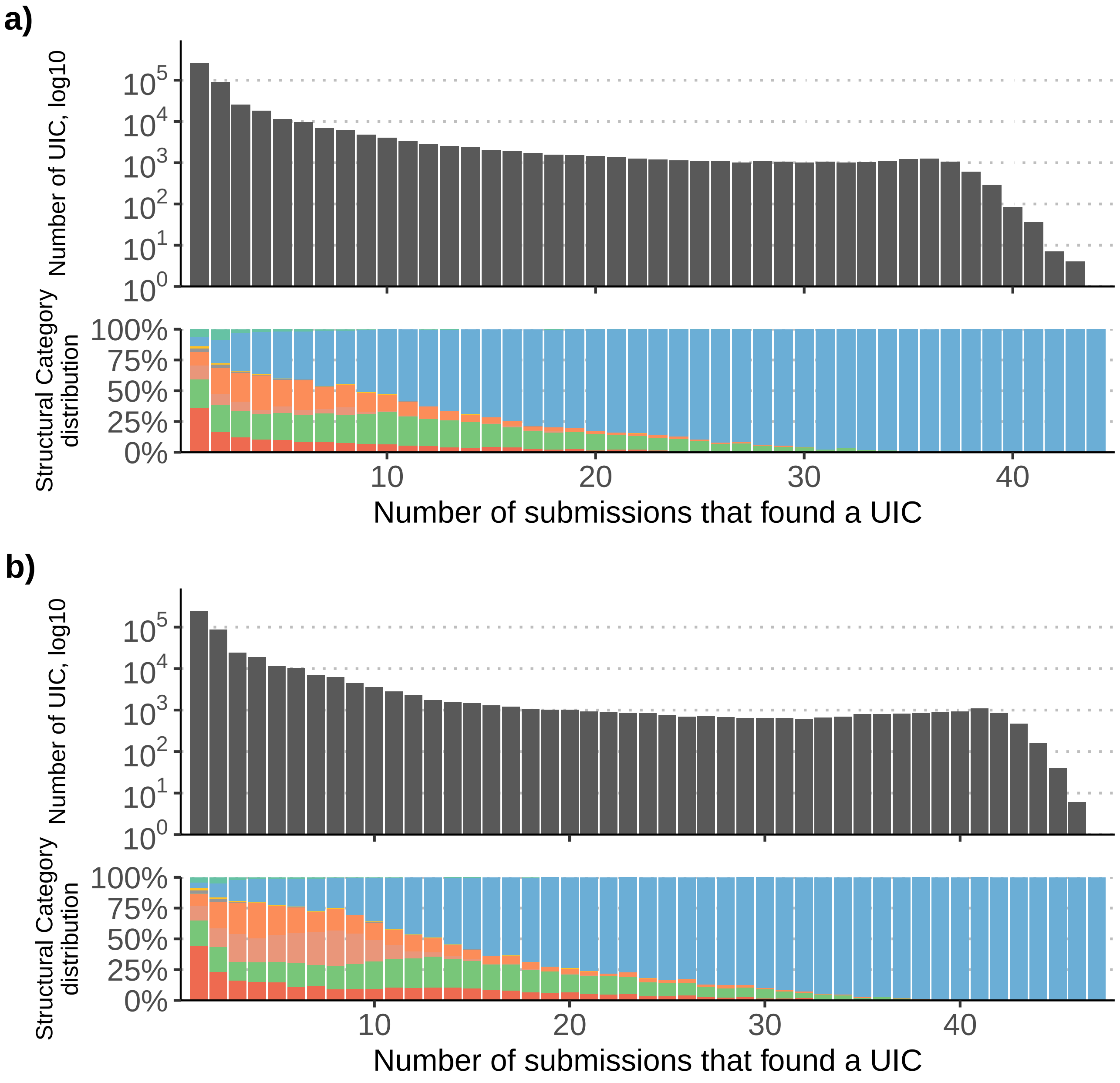

Supplementary Fig. 18. Number and SQANTI category distribution of Unique Intron Chain (UIC) consistently detected by an increasing number of submissions. a) H1-mix sample, b) Mouse ES sample.

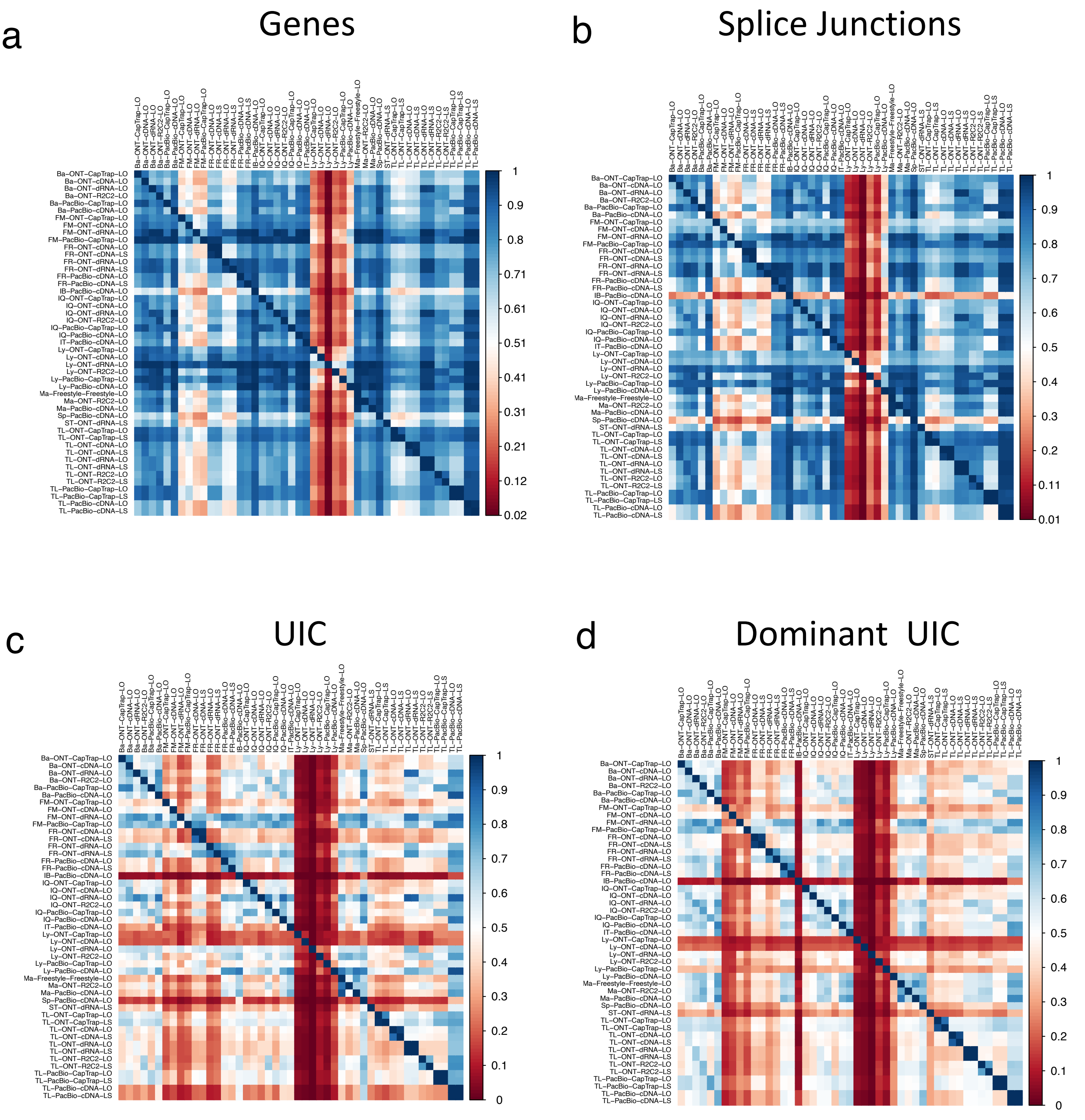

Supplementary Fig. 19. Pair-wise overlap in the detection of features between pipelines; WTC11 sample. Each value represents the feature intersection between column and row pipelines divided by the number of detections in the row pipeline. a) Genes, b) Splice junctions, c) Unique Intron Chains (UIC), c) Top UIC accounting for at least 50% of the gene expression.

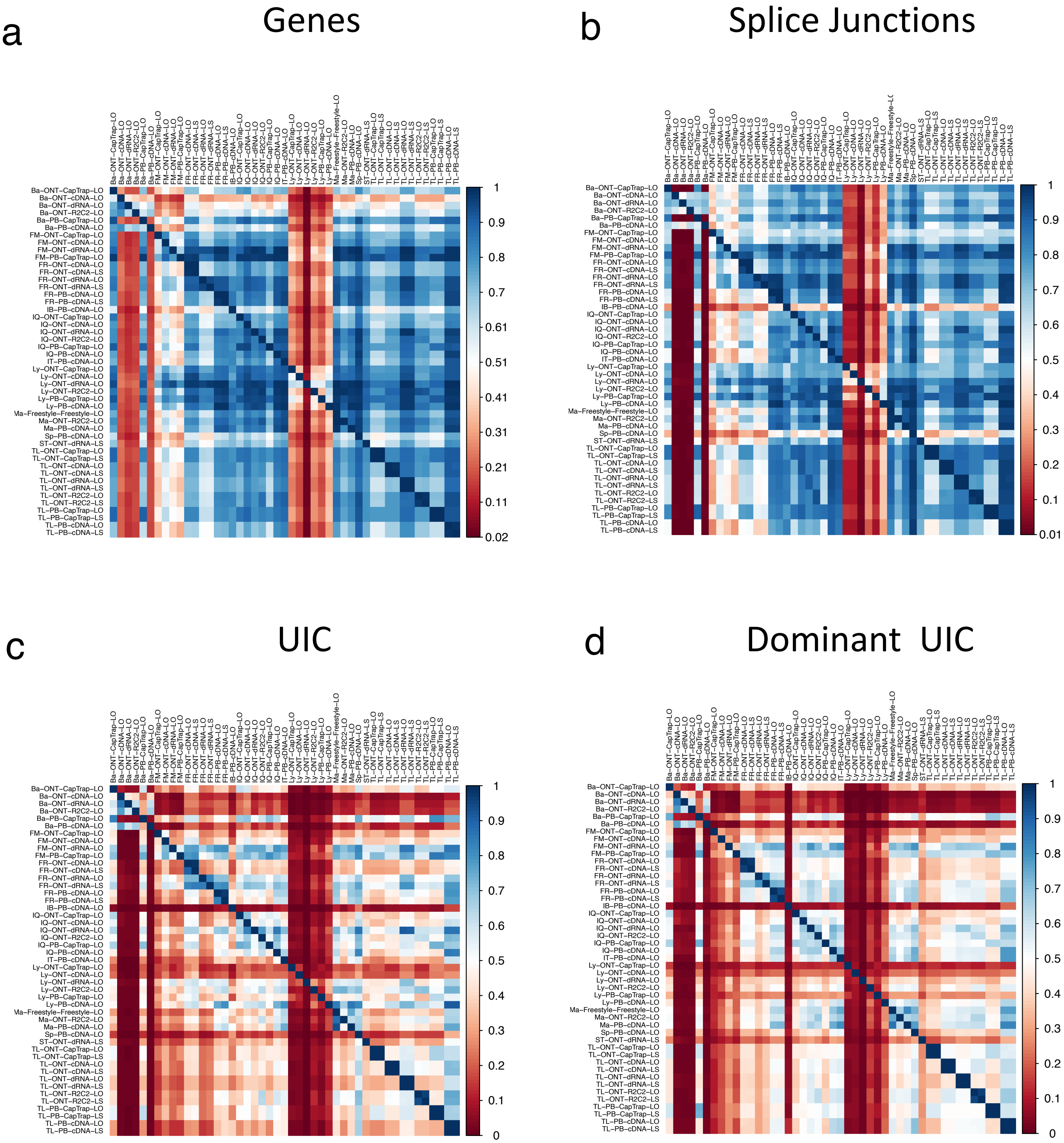

Supplementary Fig. 20. Pair-wise overlap in the detection of features between pipelines; H1-mix sample. Each value represents the feature intersection between column and row pipelines divided by the number of detections in the row pipeline. a) Genes, b) Splice junctions, c) Unique Intron Chains (UIC), c) Top UIC accounting for at least 50% of the gene expression.

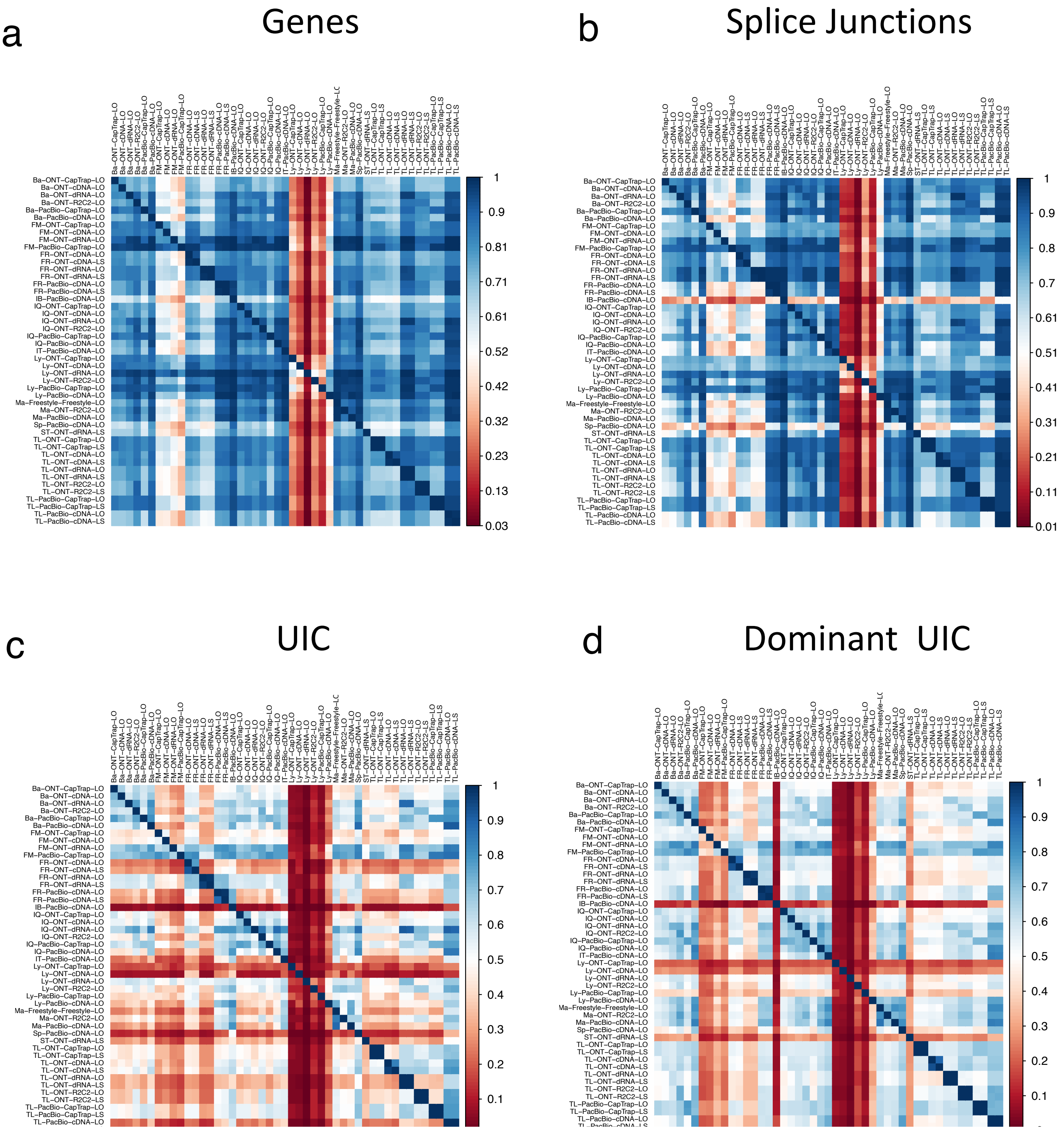

Supplementary Fig. 21. Pair-wise overlap in the detection of features between pipelines; ES mouse sample. Each value represents the feature intersection between column and row pipelines divided by the number of detections in the row pipeline. a) Genes, b) Splice junctions, c) Unique Intron Chains (UIC), c) Top UIC accounting for at least 50% of the gene expression.

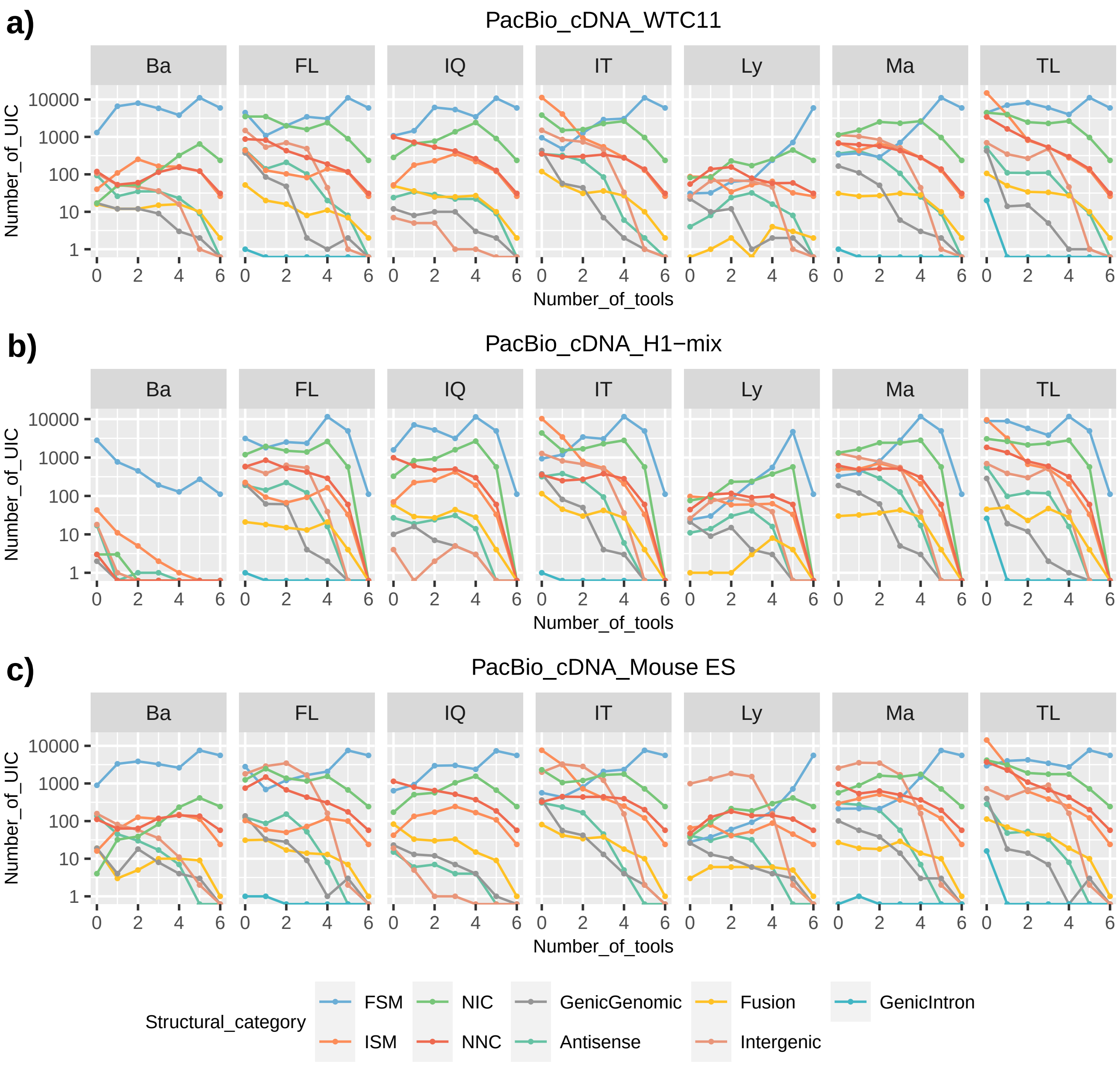

Supplementary Fig. 22. 2 Number of UIC detected by a tool and shared with an increasing number of other tools, processing PacBio_cDNA data. a) WTC11, c) H1-mix, c) Mouse ES.

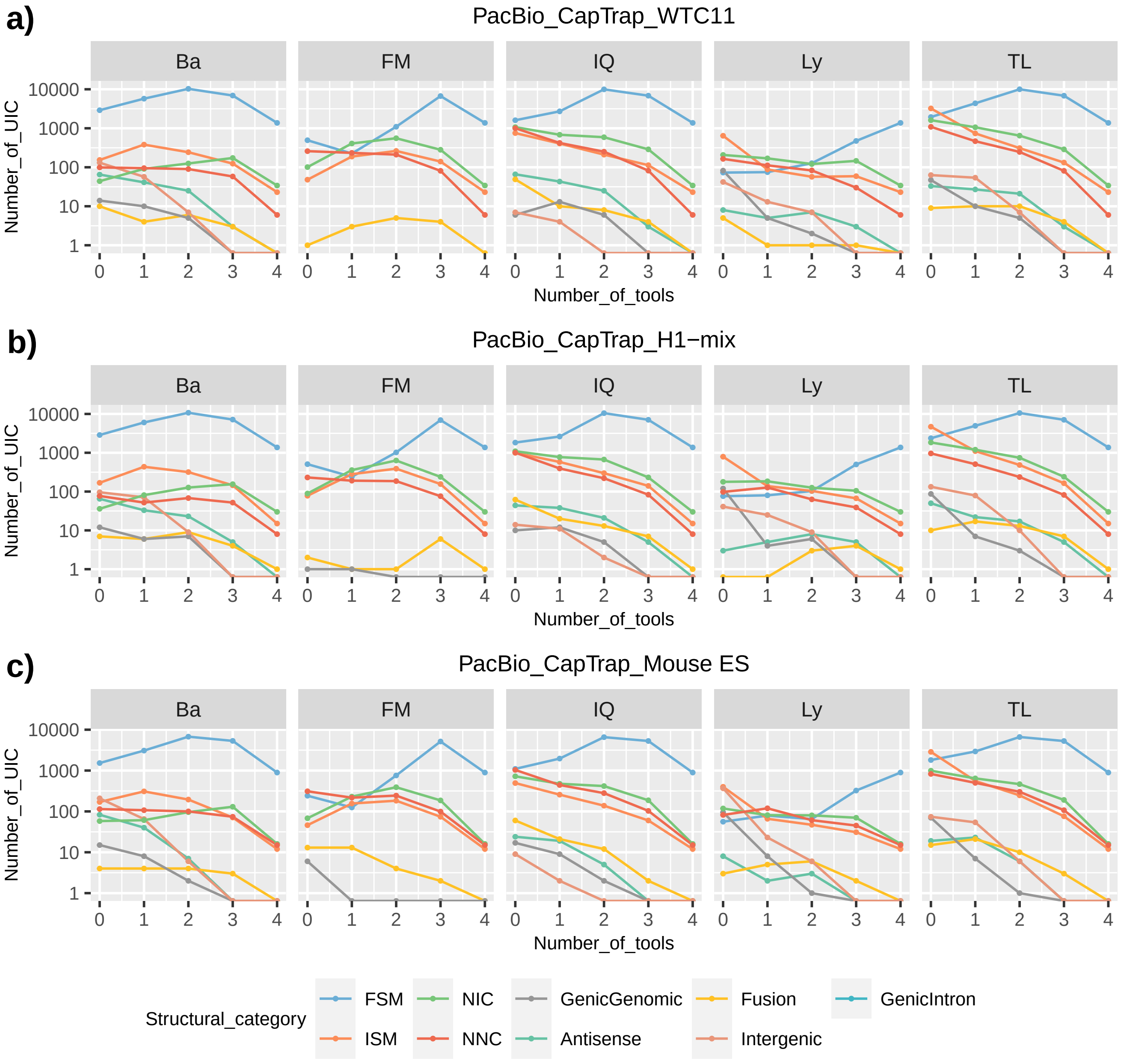

Supplementary Fig. 23. Number of UIC detected by a tool and shared with an increasing number of other tools, processing PacBio_CapTrap data. a) WTC11, c) H1-mix, c) Mouse ES.

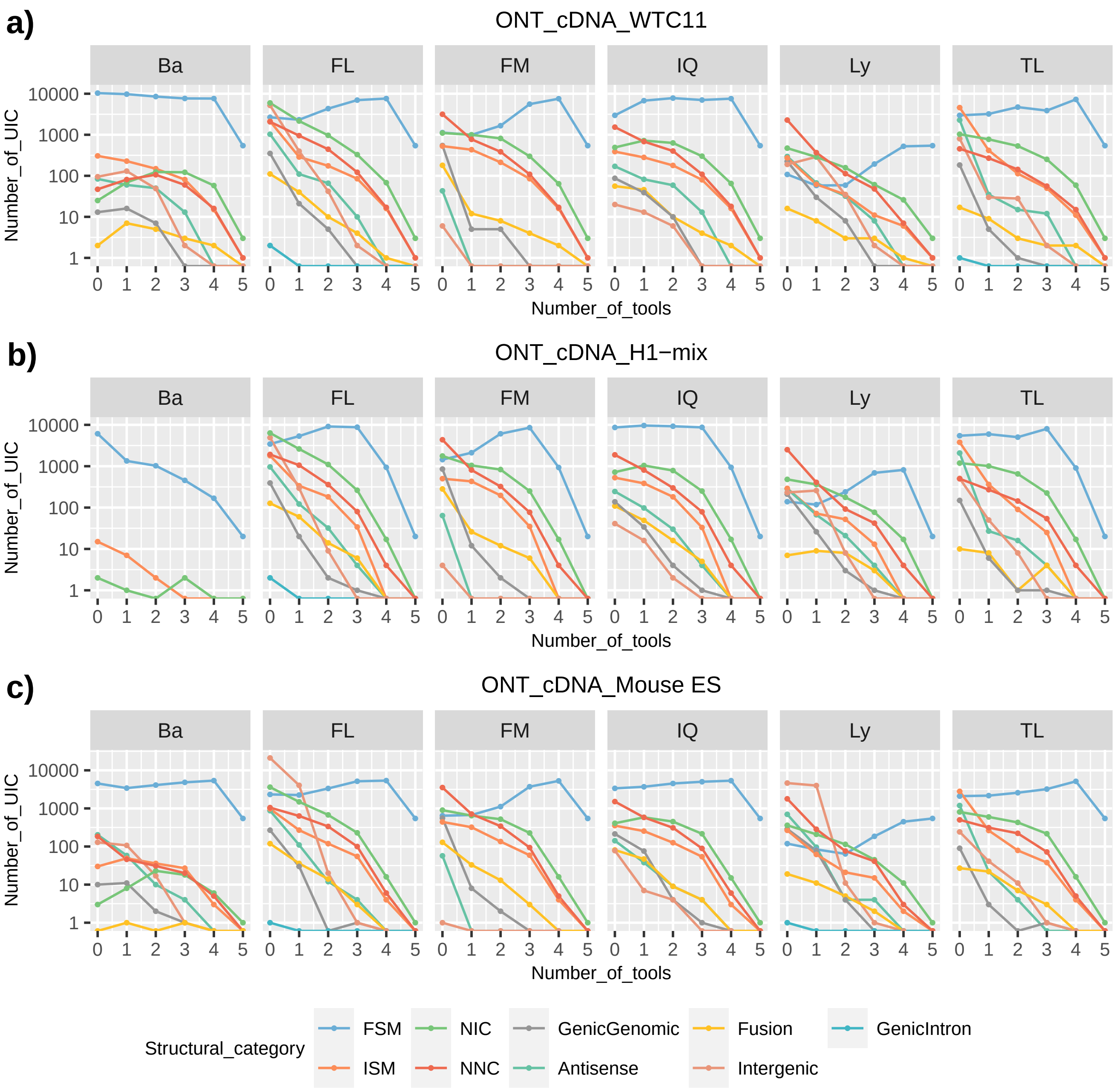

Supplementary Fig. 24. Number of UIC detected by a tool and shared with an increasing number of other tools, processing ONT_cDNA data. a) WTC11, c) H1-mix, c) Mouse ES.

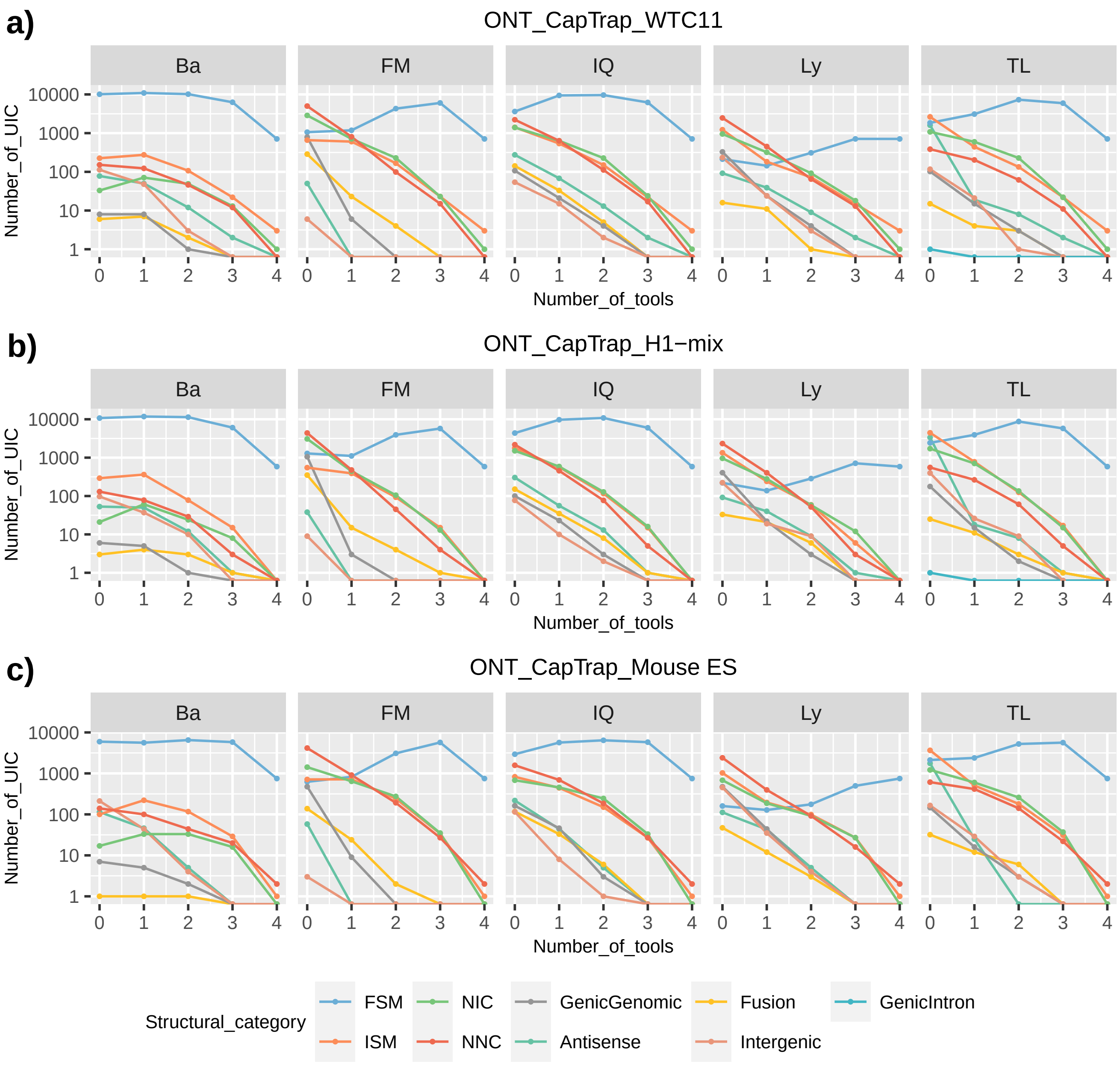

Supplementary Fig. 25. Number of UIC detected by a tool and shared with an increasing number of other tools, processing ONT_CapTrap data. a) WTC11, c) H1-mix, c) Mouse ES.

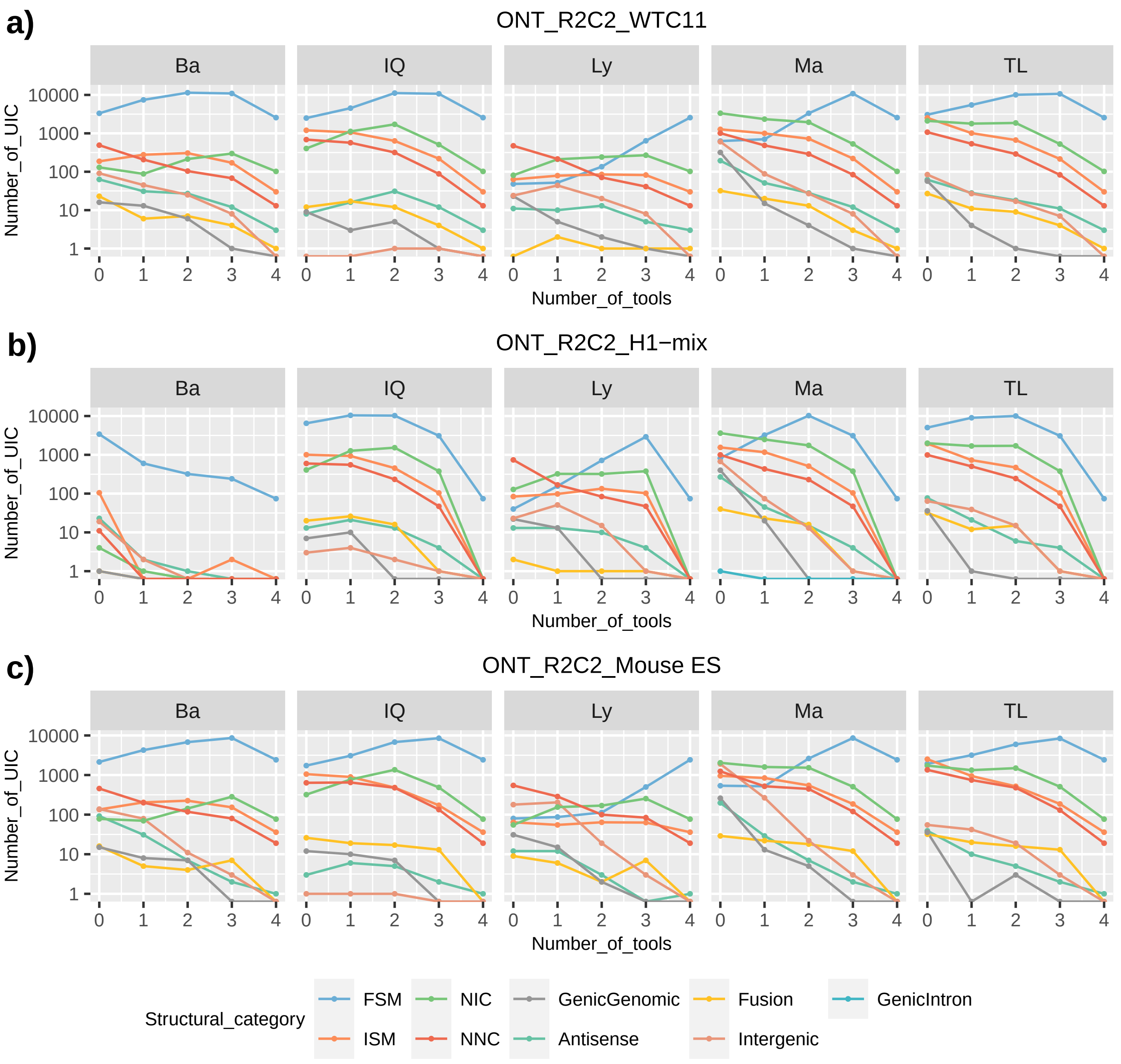

Supplementary Fig. 26. Number of UIC detected by a tool and shared with an increasing number of other tools, processing ONT_R2C2 data. a) WTC11, c) H1-mix, c) Mouse ES

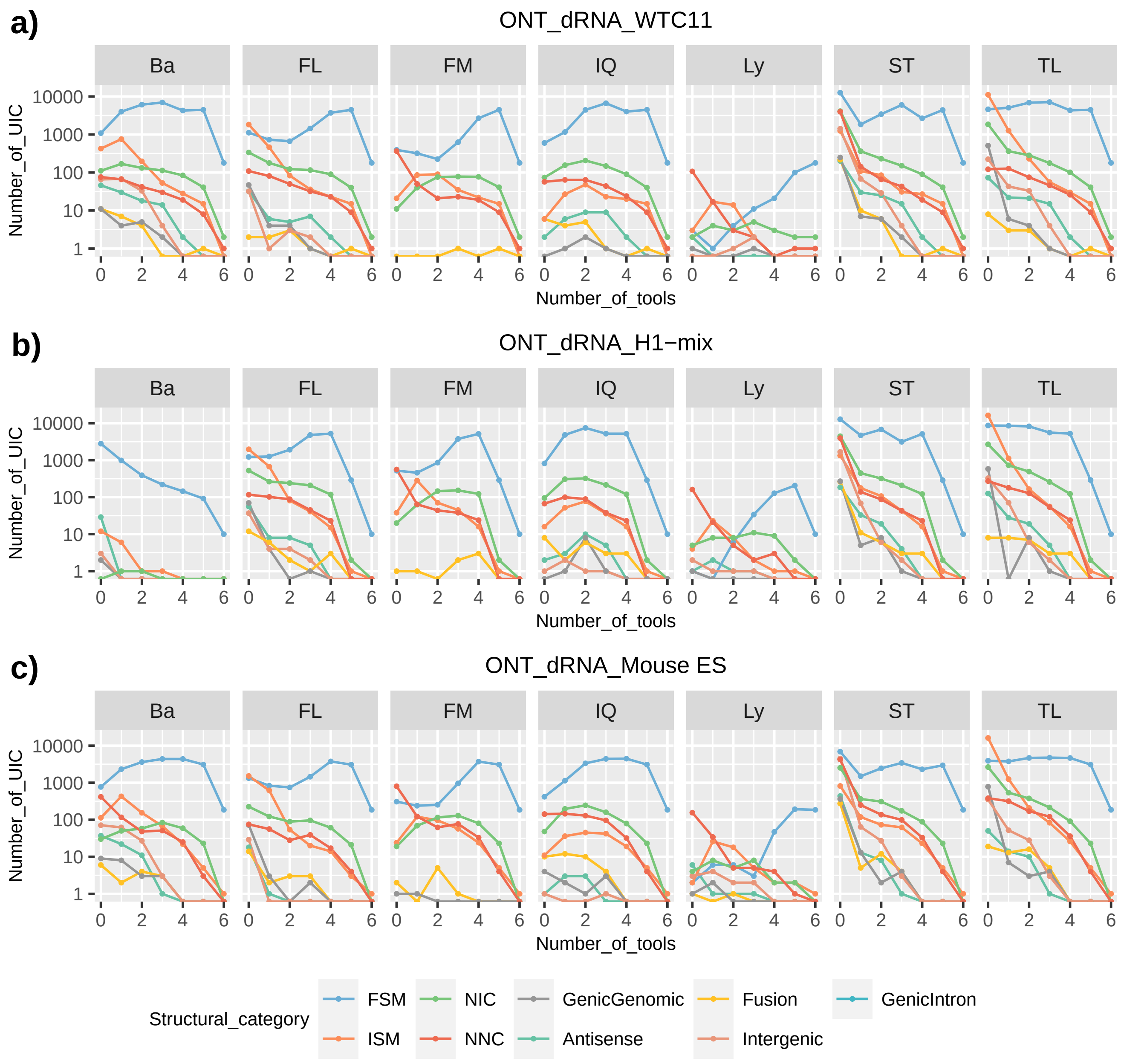

Supplementary Fig. 27. Number of UIC detected by a tool and shared with an increasing number of other tools, processing ONT_dRNA data. a) WTC11, c) H1-mix, c) Mouse ES

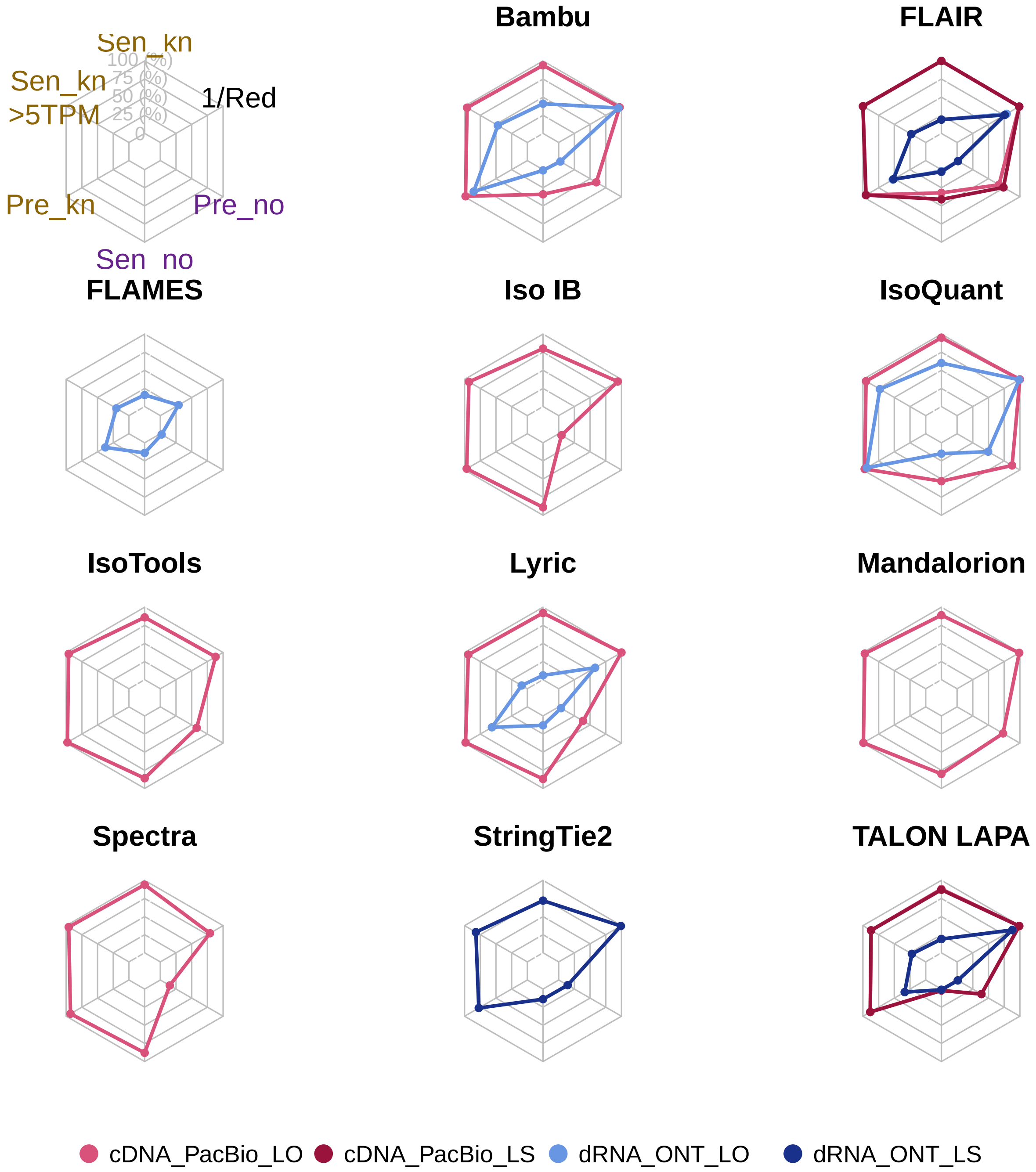

Supplementary Fig. 28. Performance metrics on mouse simulated data. Sen_kn: sensitivity known transcripts, Sen_kn ¿ 5 TPM: sensitivity known transcripts with expression ¿ 5 TPM, Pre_kn: precision known transcripts, Sen_no: sensitivity novel transcripts, Pre_no: precision novel transcripts, 1/Red: inverse of redundancy.

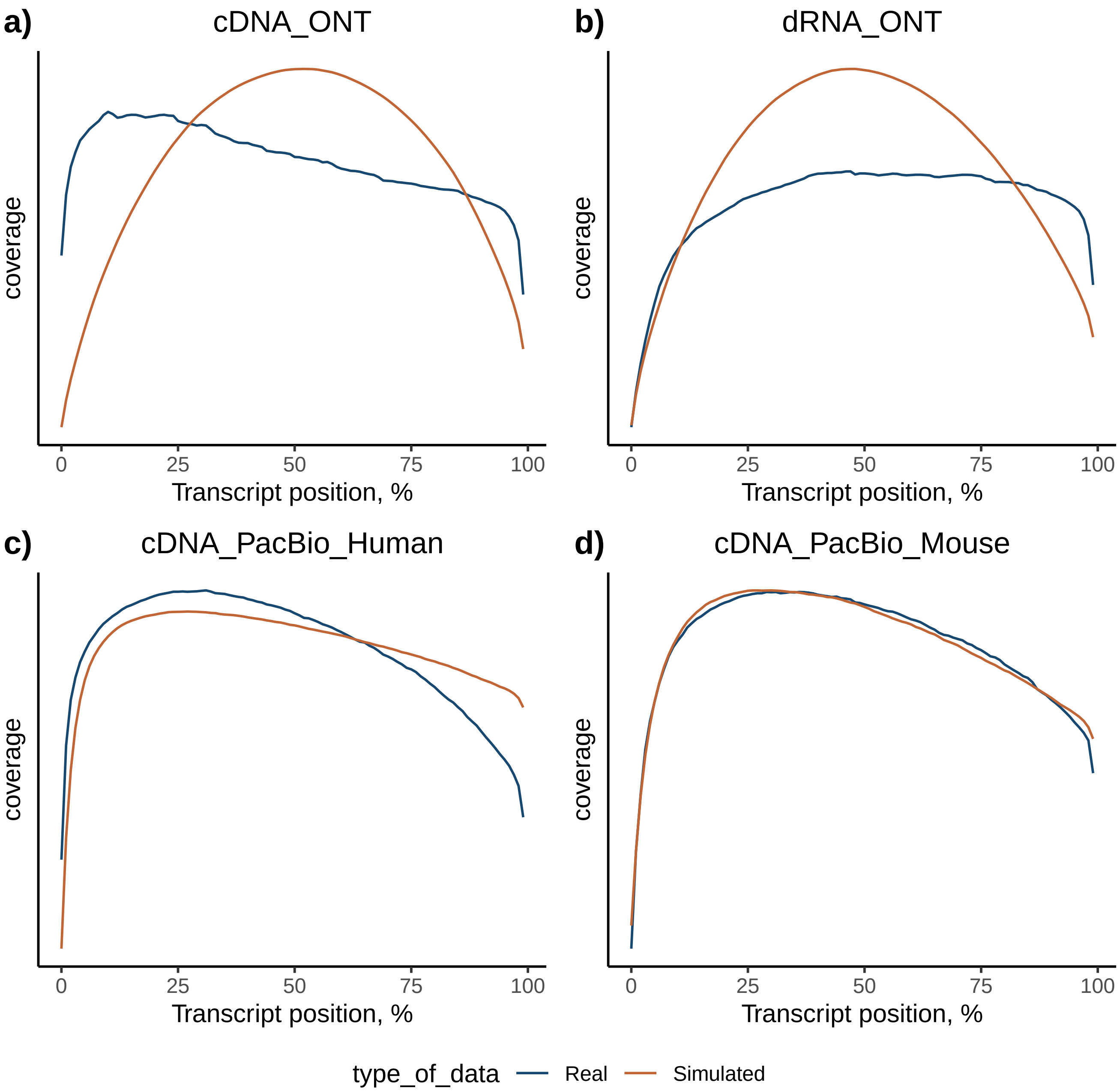

Supplementary Fig. 29. Comparison of long-read transcript coverage between real and simulated datasets.

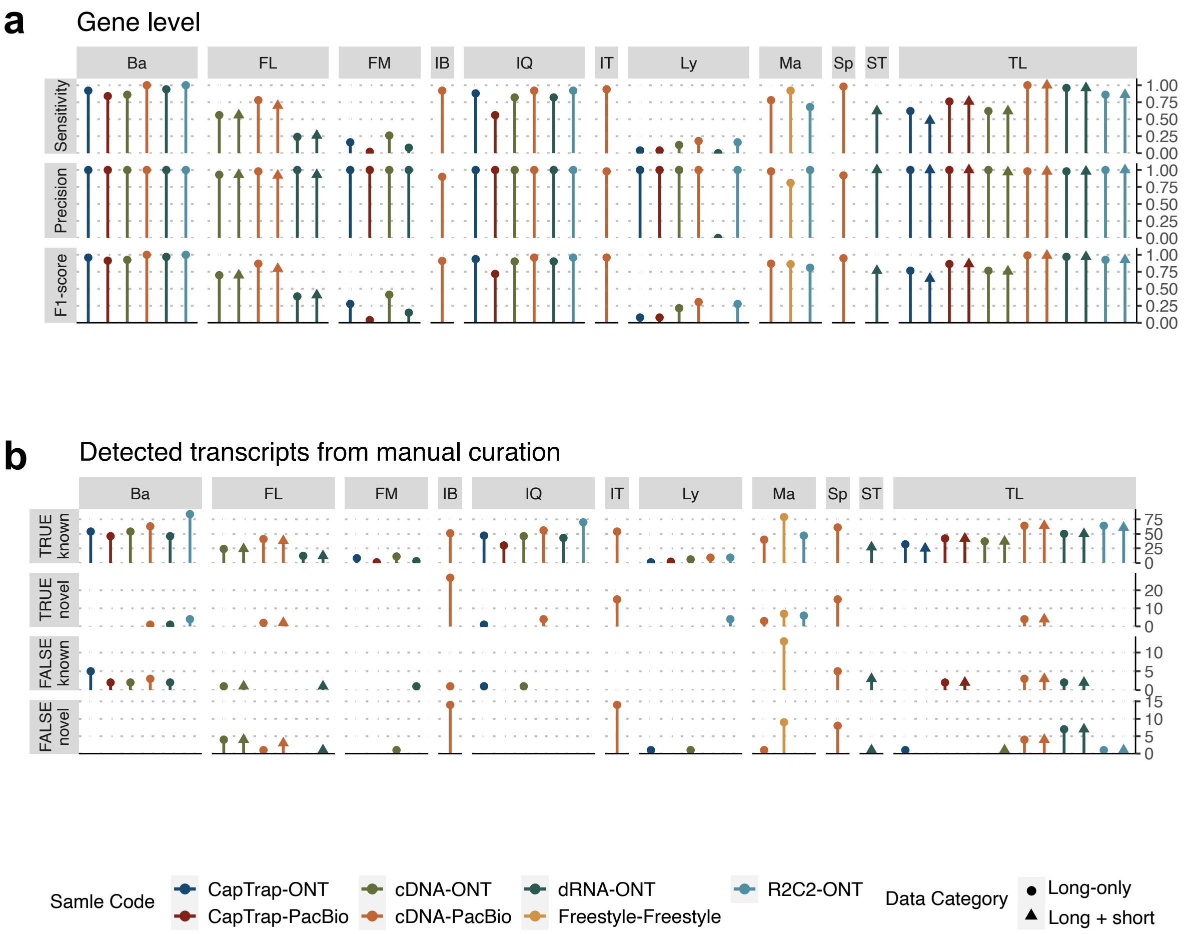

Supplementary Fig. 30. Performance of tools on a) genes and b) detection of curated transcript from manual annotation of 50 human genes manually-annotated by GENCODE. Tools are: Ba: Bambu, FM: Flames, FR: FLAIR, IQ: IsoQuant, IT: IsoTools, IB: Iso_IB, Ly: LyRic, Ma: Mandalorion, TL: TALON-LAPA, Sp: Spectra, ST: StringTie2.

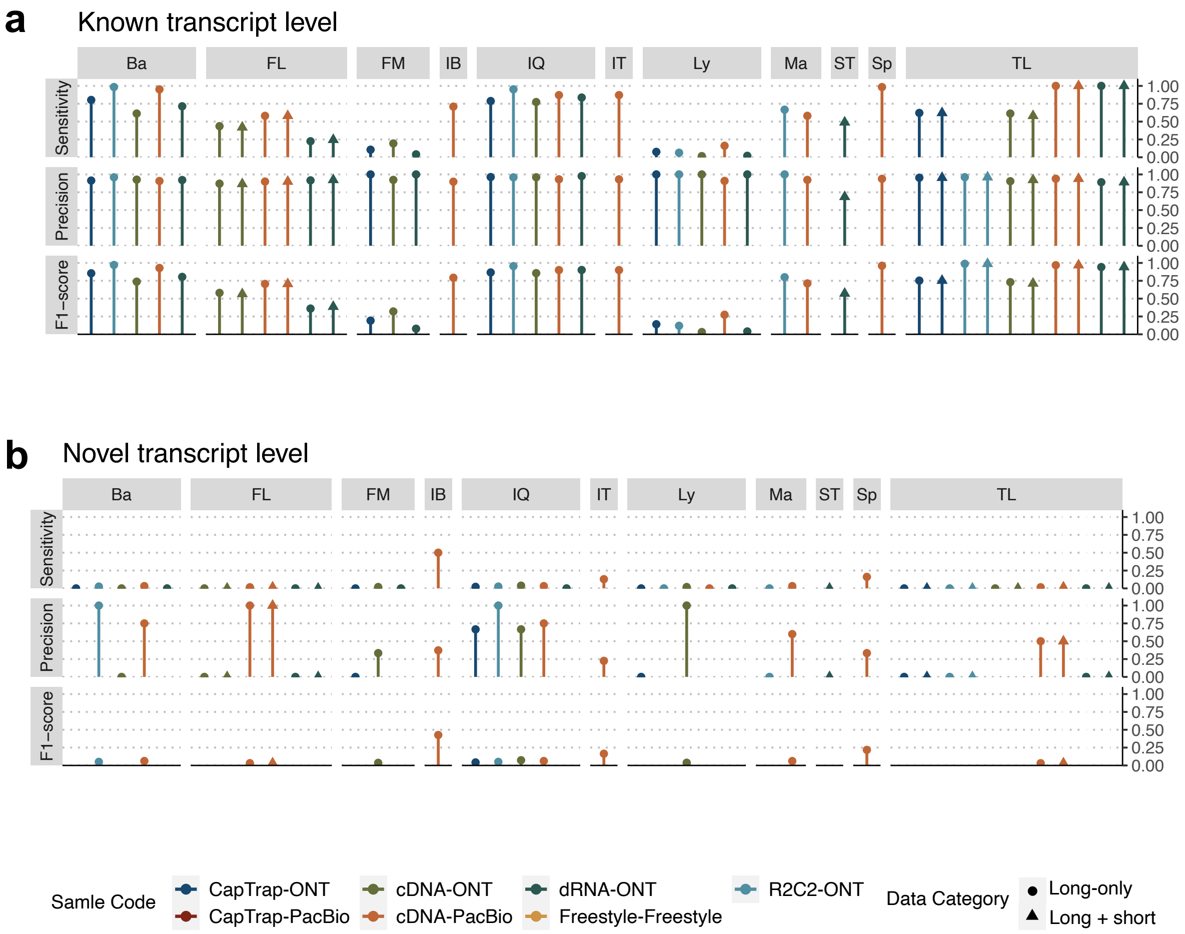

Supplementary Fig. 31. Performance of tools on a) known transcript, and b) novel transcripts from manual annotation of 50 mouse genes manually-annotated by GENCODE. Tools are: Ba: Bambu, FM: Flames, FR: FLAIR, IQ: IsoQuant, IT: IsoTools, IB: Iso_IB, Ly: LyRic, Ma: Mandalorion, TL: TALON-LAPA, Sp: Spectra, ST: StringTie2.

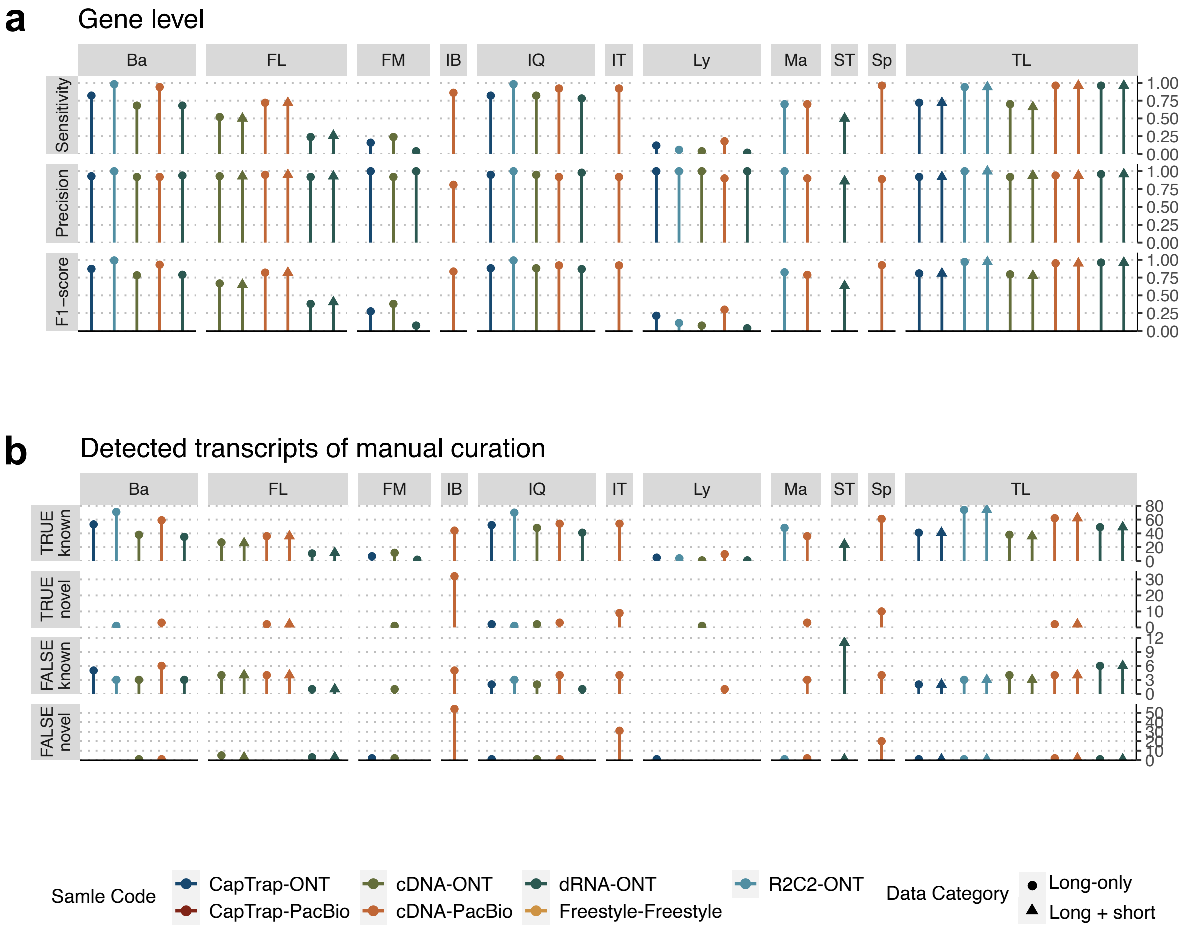

Supplementary Fig. 32. Performance of tools on detection of a) curated transcript, and b) genes from manual annotation of 50 mouse genes manually-annotated by GENCODE. Tools are: Ba: Bambu, FM: Flames, FR: FLAIR, IQ: IsoQuant, IT: IsoTools, IB: Iso_IB, Ly: LyRic, Ma: Mandalorion, TL: TALON-LAPA, Sp: Spectra, ST: StringTie2.

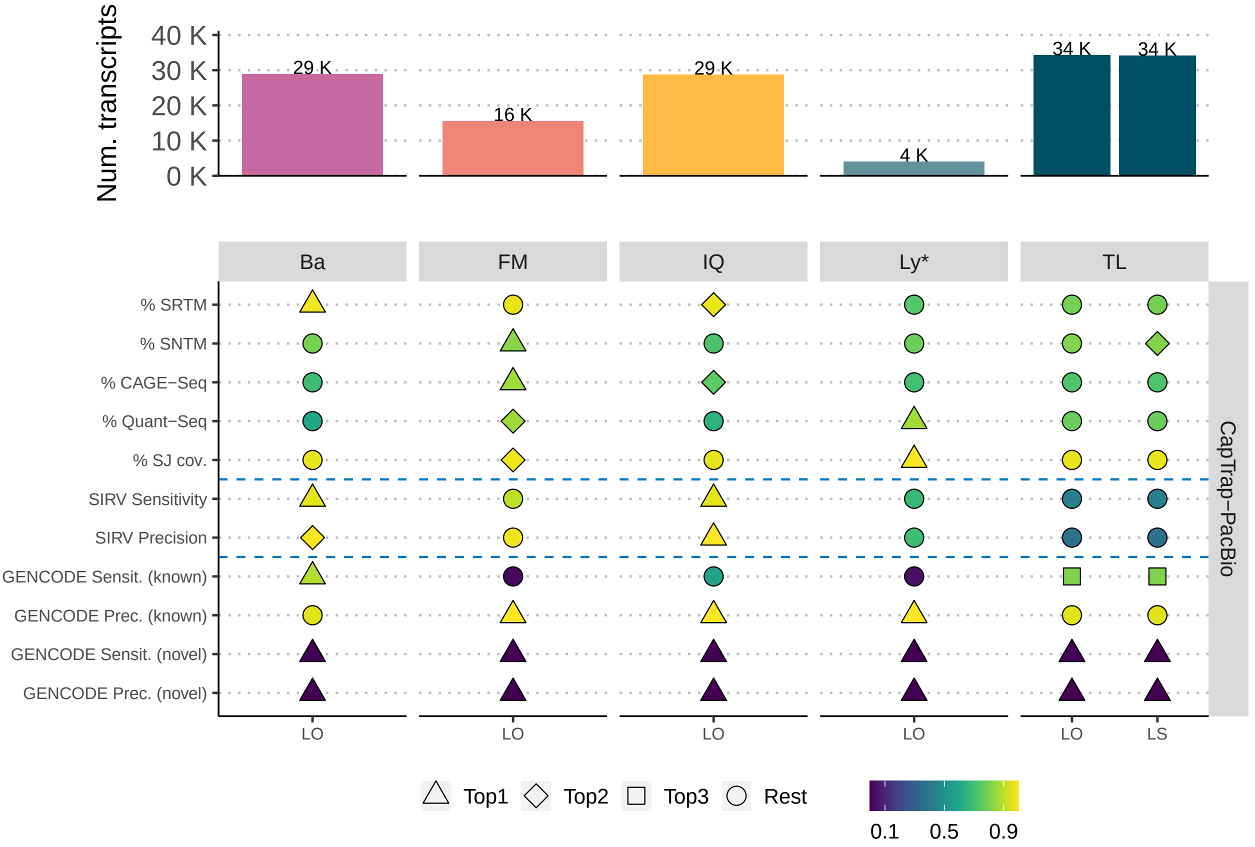

Supplementary Fig. 33. Summary of performance metrics of tools for CapTrap-PacBio benchmarking dataset. Color scale represents the performance value ranging from worse (dark blue) to better (light yellow). Graphic symbol indicates the raking position of the tool for the metric represented in each row. SJ: Splice Junction, UIC: Unique Intron Chain. LO: Long (reads) Only, LS: Long and Short (reads), Sen_kn: Sensitivity for known transcripts, Pre_kn: Precision for known transcripts, Sen_no: Sensitivity for Novel transcripts, Pre_no: Precision for Novel transcripts, 1/Red: inverse of redundancy. Num: number, SRTM: Supported Reference Transcript Model, SNTM: Supported Novel Transcript Model, Ba: Bambu, FM: Flames, FR: FLAIR, IQ: IsoQuant, IT: IsoTools, IB: Iso_IB, Ly: LyRic, Ma: Mandalorion, TL: TALON-LAPA, Sp: Spectra, ST: StringTie2.

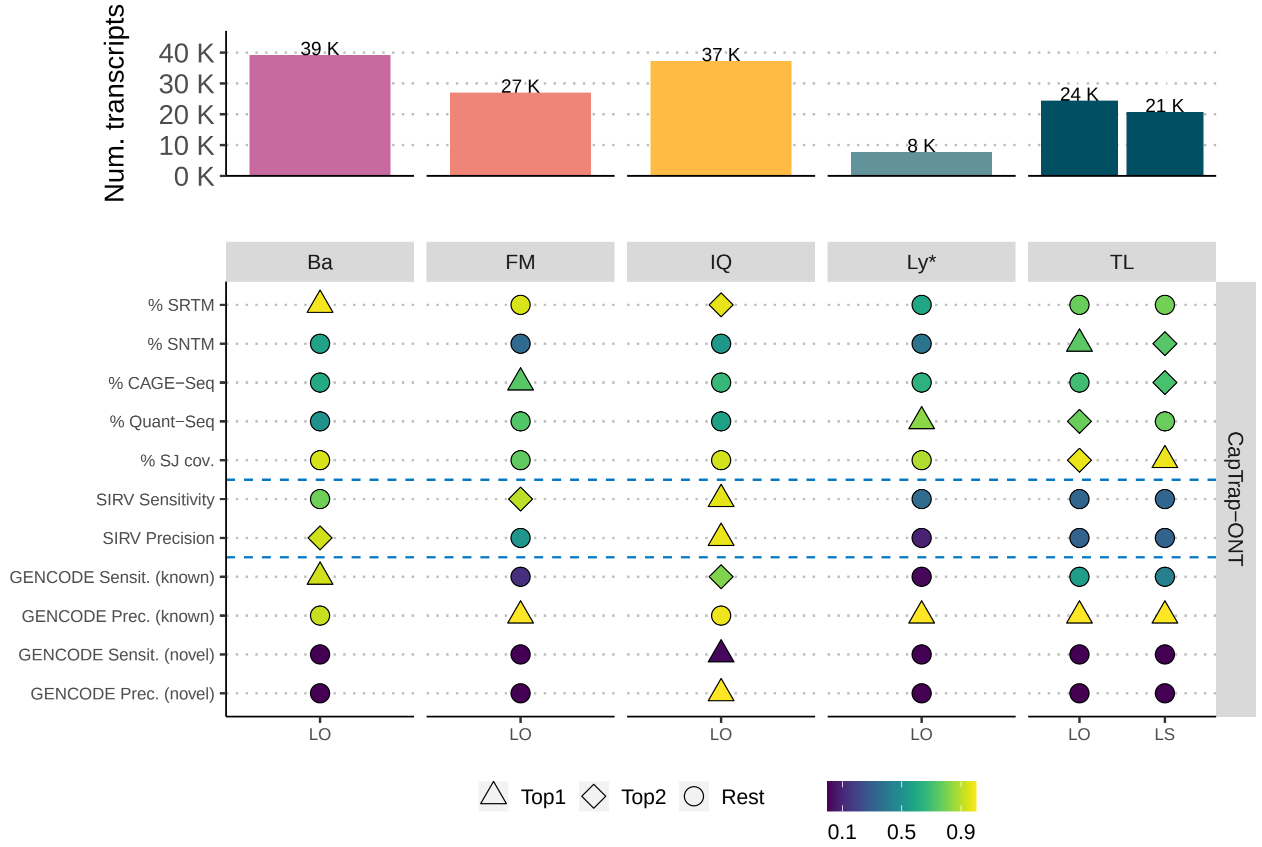

Supplementary Fig. 34. Summary of performance metrics of tools for the CapTrap-ONT benchmarking dataset. Color scale represents the performance value ranging from worse (dark blue) to better (light yellow). Graphic symbol indicates the raking position of the tool for the metric represented in each row. SJ: Splice Junction, UIC: Unique Intron Chain. LO: Long (reads) Only, LS: Long and Short (reads), Sen_kn: Sensitivity for known transcripts, Pre_kn: Precision for known transcripts, Sen_no: Sensitivity for Novel transcripts, Pre_no: Precision for Novel transcripts, 1/Red: inverse of redundancy. Num: number, SRTM: Supported Reference Transcript Model, SNTM: Supported Novel Transcript Model, Ba: Bambu, FM: Flames, FR: FLAIR, IQ: IsoQuant, IT: IsoTools, IB: Iso_IB, Ly: LyRic, Ma: Mandalorion, TL: TALON-LAPA, Sp: Spectra, ST: StringTie2.

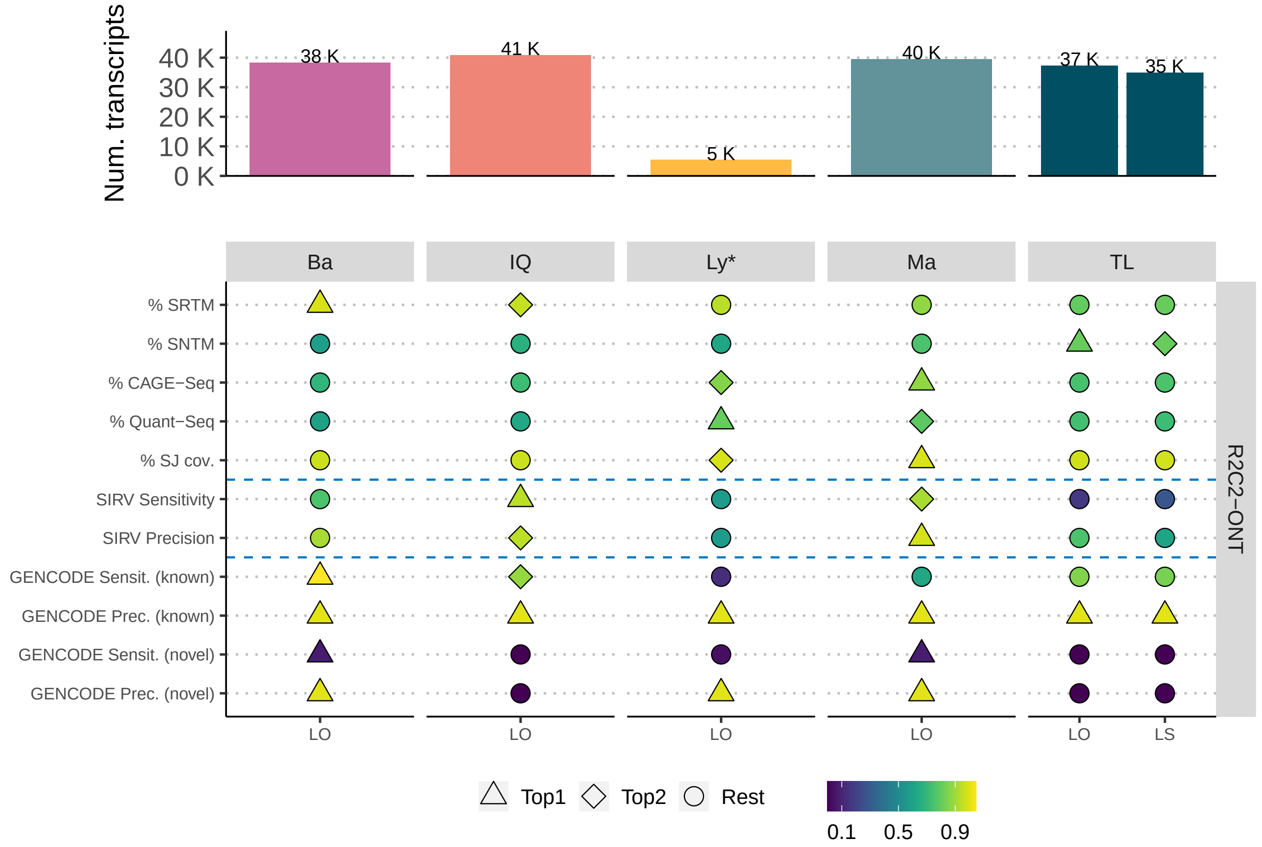

Supplementary Fig. 35. Summary of performance metrics of tools for the R2C2-ONT benchmarking dataset. Color scale represents the performance value ranging from worse (dark blue) to better (light yellow). Graphic symbol indicates the raking position of the tool for the metric represented in each row. SJ: Splice Junction, UIC: Unique Intron Chain. LO: Long (reads) Only, LS: Long and Short (reads), Sen_kn: Sensitivity for known transcripts, Pre_kn: Precision for known transcripts, Sen_no: Sensitivity for Novel transcripts, Pre_no: Precision for Novel transcripts, 1/Red: inverse of redundancy. Num: number, SRTM: Supported Reference Transcript Model, SNTM: Supported Novel Transcript Model, Ba: Bambu, FM: Flames, FR: FLAIR, IQ: IsoQuant, IT: IsoTools, IB: Iso_IB, Ly: LyRic, Ma: Mandalorion, TL: TALON-LAPA, Sp: Spectra, ST: StringTie2.

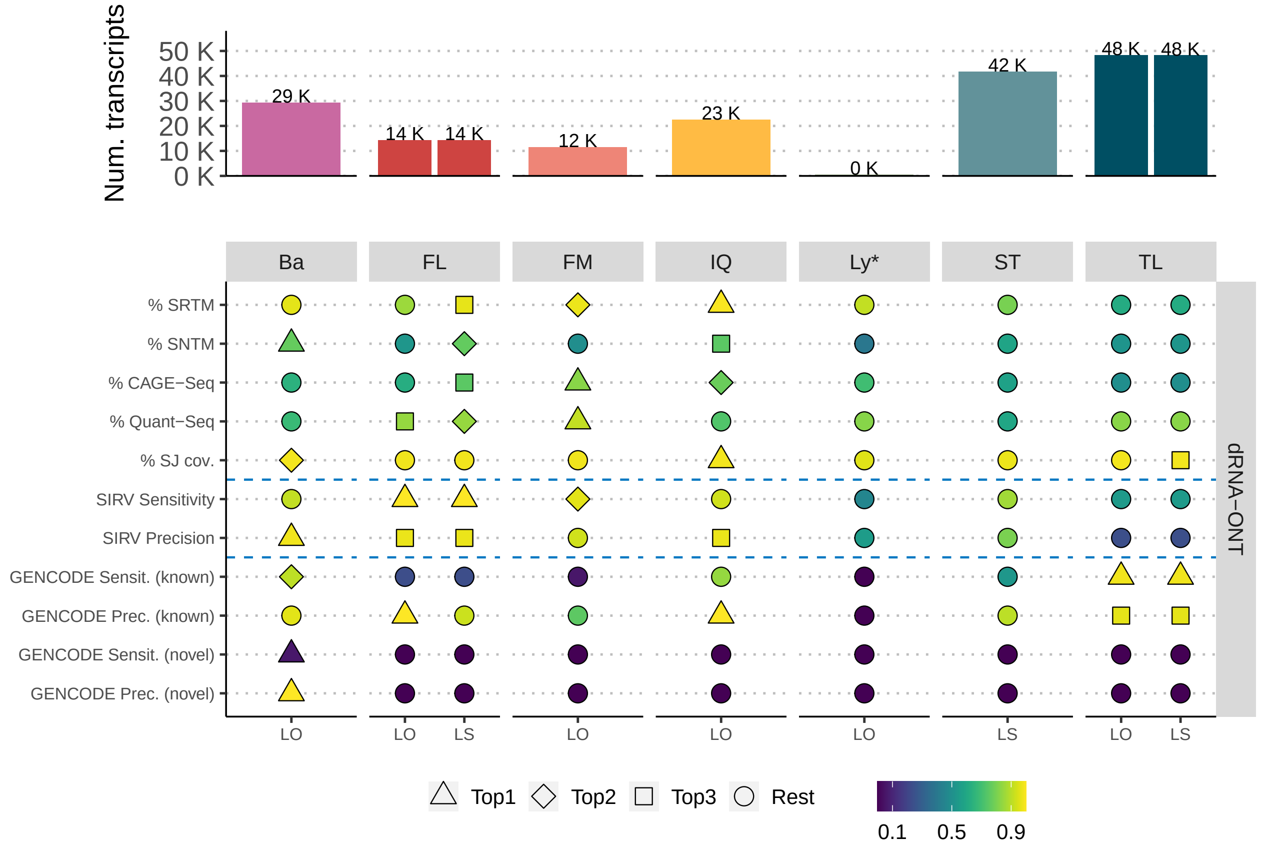

Supplementary Fig. 36. Summary of performance metrics of tools for the dRNA-ONT benchmarking dataset. Color scale represents the performance value ranging from worse (dark blue) to better (light yellow). Graphic symbol indicates the raking position of the tool for the metric represented in each row. SJ: Splice Junction, UIC: Unique Intron Chain. LO: Long (reads) Only, LS: Long and Short (reads), Sen_kn: Sensitivity for known transcripts, Pre_kn: Precision for known transcripts, Sen_no: Sensitivity for Novel transcripts, Pre_no: Precision for Novel transcripts, 1/Red: inverse of redundancy. Num: number, SRTM: Supported Reference Transcript Model, SNTM: Supported Novel Transcript Model, Ba: Bambu, FM: Flames, FR: FLAIR, IQ: IsoQuant, IT: IsoTools, IB: Iso_IB, Ly: LyRic, Ma: Mandalorion, TL: TALON-LAPA, Sp: Spectra, ST: StringTie2.

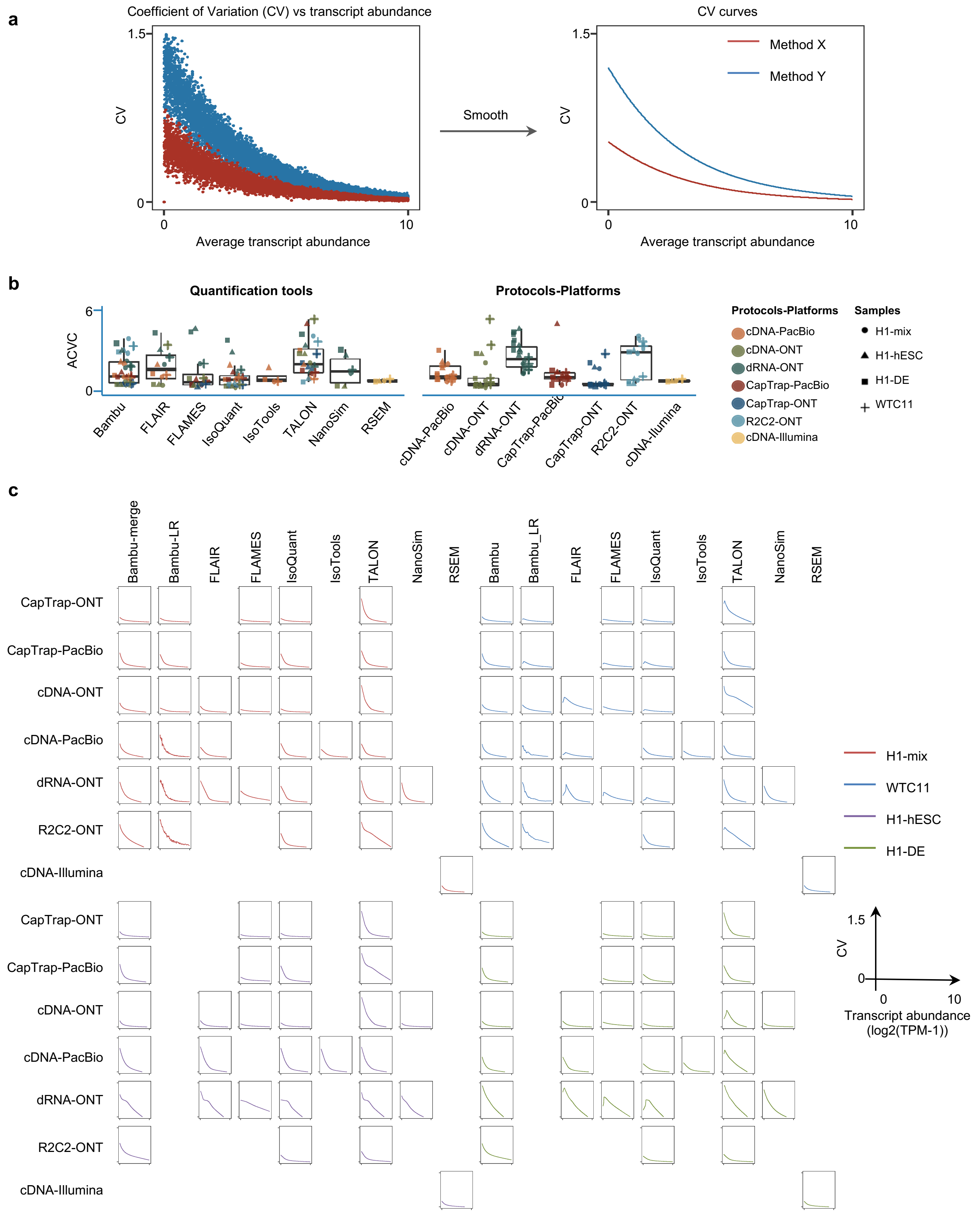

Supplementary Fig. 37. Overall evaluation results of irreproducibility on real data with multiple replicates. The diagram illustrates the calculation of irreproducibility. By fitting the coefficient of variation (CV) versus average transcript abundance into a smooth curve, it can be shown that Method X has lower coefficient of variation and higher reproducibility. Evaluation results of ACVC metric for different quantification tools and protocols-platforms. Box plots are employed to illustrate the five-number summary of evaluation results across various datasets, depicting the minimum, lower quartile, median, upper quartile, and maximum values. The overall results of CV curves with different transcript abundances on four samples (H1-mix, WTC11, H1-hESC and H1-DE) with different protocols and platforms. Here, Bambu-merge represents the transcript quantification using Bambu with GENCODE plus LR-specific annotation. And Bambu-LR represents the transcript quantification using only LR-specific annotation.

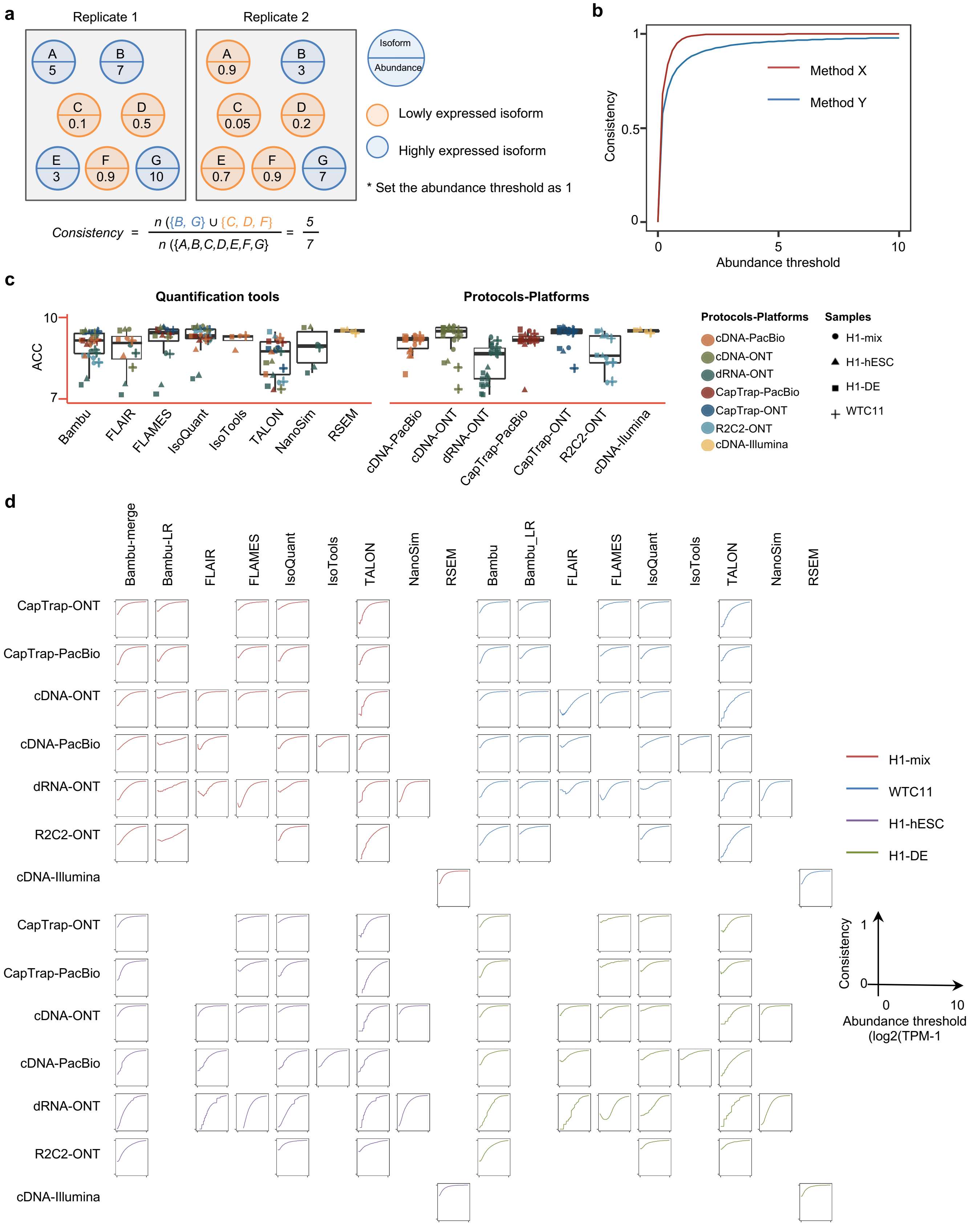

Supplementary Fig. 38. Overall evaluation results of consistency on real data with multiple replicates. a) The diagram illustrates the calculation of consistency. By setting an expression threshold (i.e. 1 in this toy example), we can define which set of transcripts express (in blue) or not (in orange). This statistic is to measure the consistency of the expressed transcripts sets between replicates. b) A toy example to show the consistency curves with different abundance threshold. Here, method X performs the better consistency of transcript abundance estimation across multiple replicates than method Y. c) Evaluation results of ACC metric for different quantification tools and protocols-platforms. Box plots are employed to illustrate the five-number summary of evaluation results across various datasets, depicting the minimum, lower quartile, median, upper quartile, and maximum values. d) The detailed evaluation results of consistency curves with different abundance thresholds on four samples (H1-mix, WTC11, H1-hESC and H1-DE) with different protocols and platforms.

Resolution Entropy

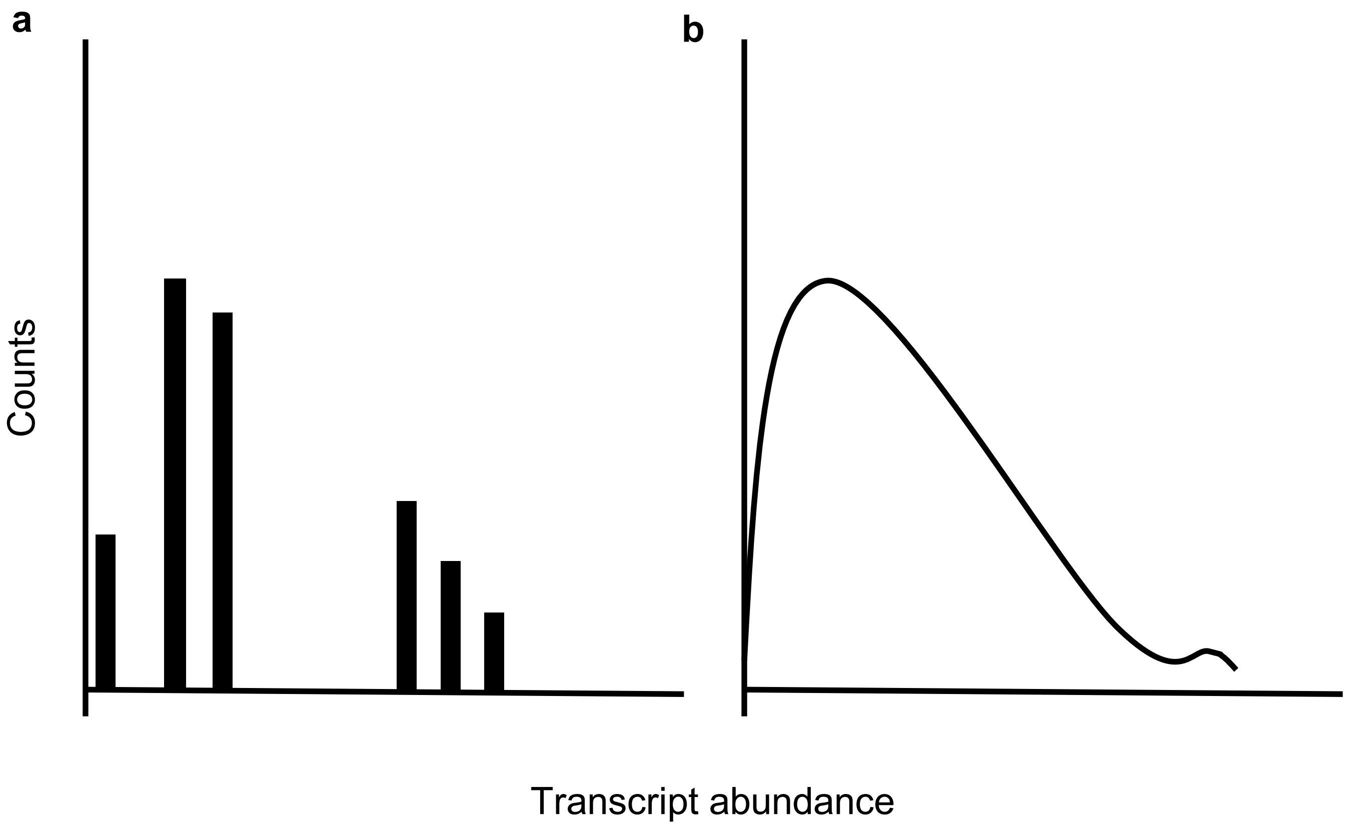

Supplementary Fig. 39. Resolution Entropy. a) The software output only a few certain discrete values has lower resolution entropy as it cannot capture the continuous and subtle difference of gene expressions. b) The software with continuous output values has higher resolution entropy.

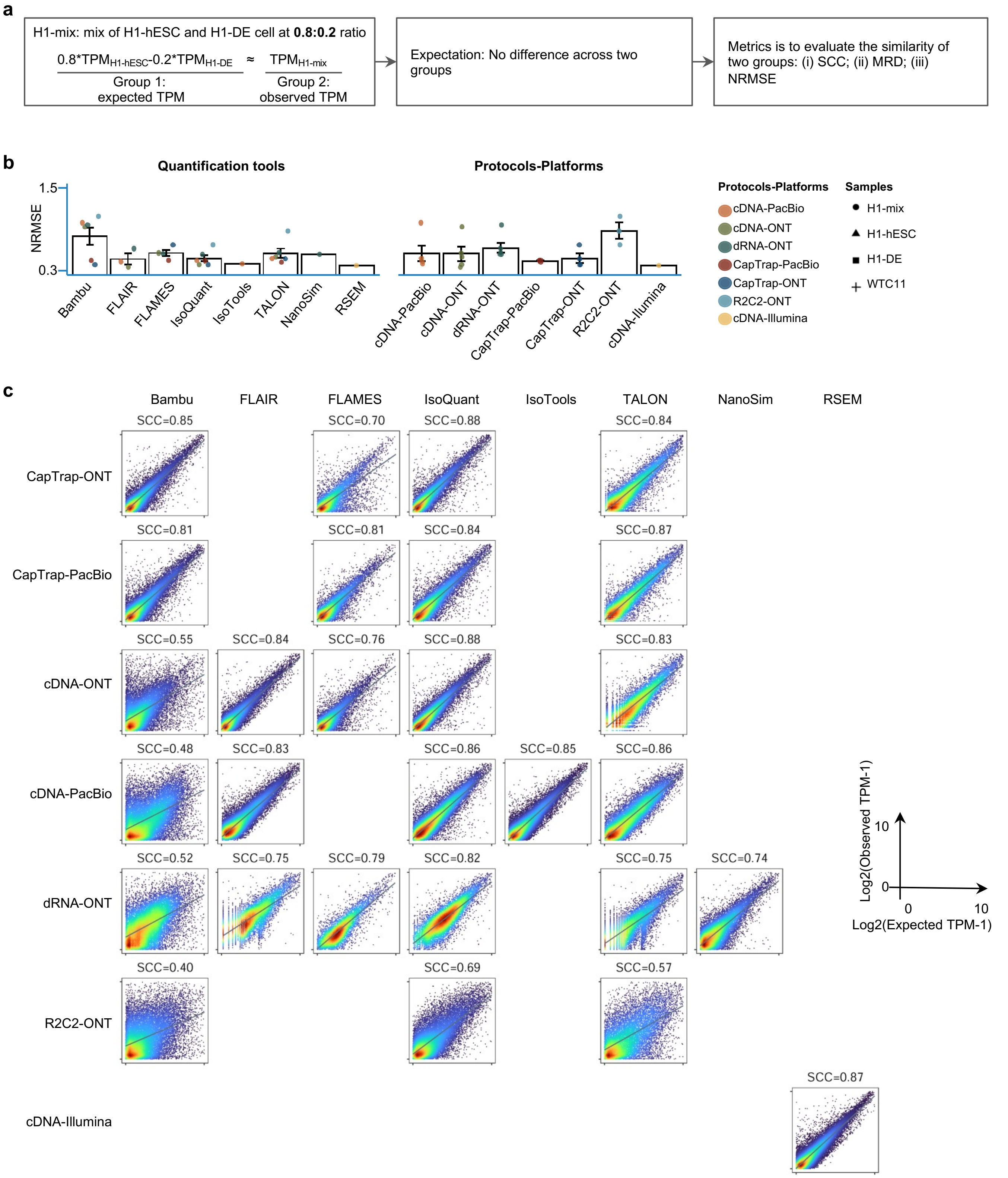

Supplementary Fig. 40. Performance evaluation on cell mixing experiment. a) Schematic diagram of evaluation strategy using the cell mixing experiment. Here, H1-mix was initially provided for quantification which was a mix of H1-hESC cells and H1-DE cells at an undisclosed ratio. After the initial submission, the individual H1-hESC and H1-DE samples were released and participants submitted quantifications for each. b) Evaluation results of NRMSE metric for different quantification tools and protocols-platforms. Bar plots are utilized to visualize the mean values of evaluation results across diverse datasets, with error bars indicating the standard deviation of metrics. c) Scatter plot of expected abundance and observed abundance for seven participant's tools with different protocols and platforms.

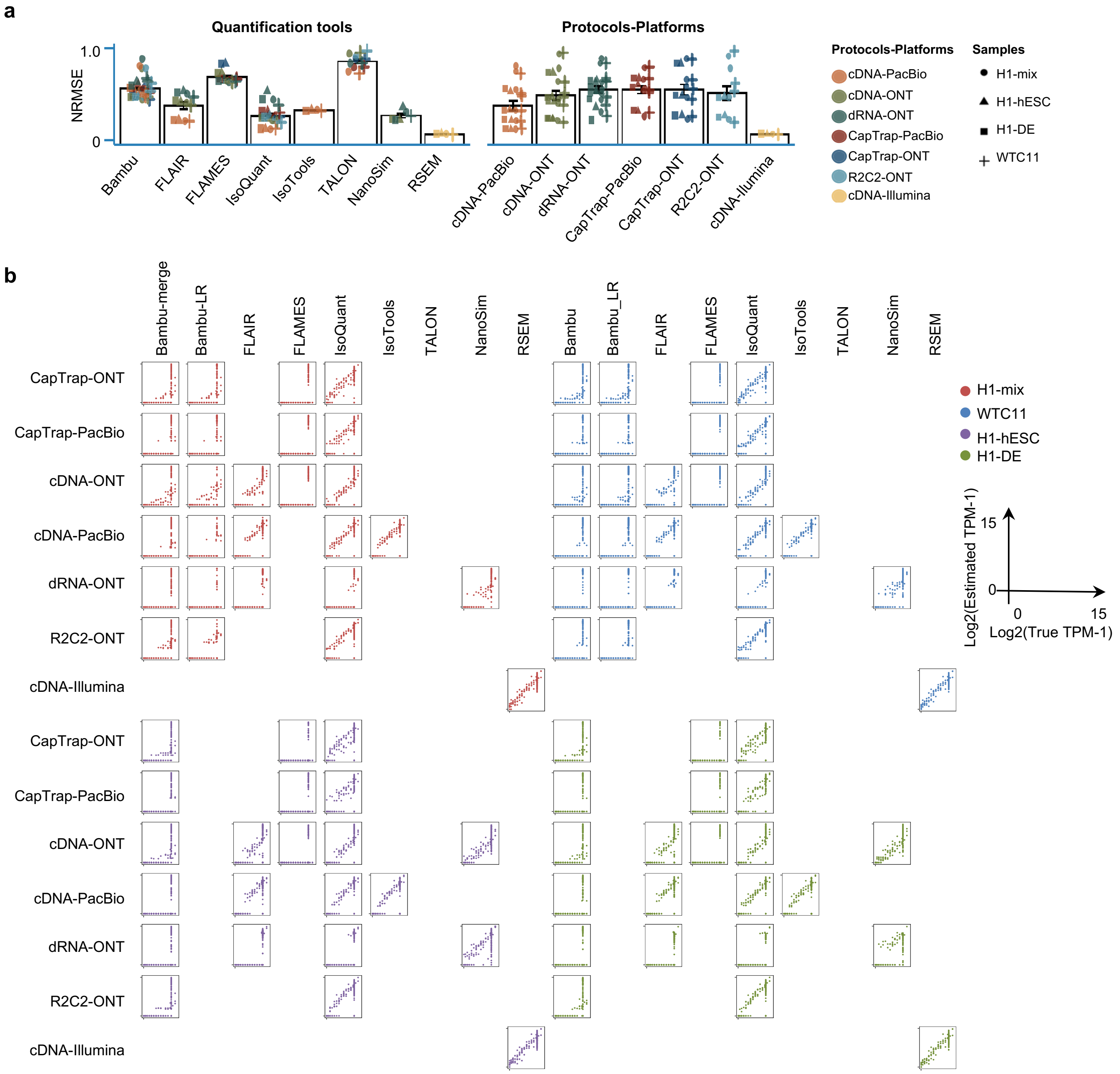

Supplementary Fig. 41. Performance evaluation on SIRV-set 4 data. a) Evaluation results of NRMSE metric for different quantification tools and protocols-platforms. Bar plots are utilized to visualize the mean values of evaluation results across diverse datasets, with error bars indicating the standard deviation of NRMSE metric. b) Scatter plot of true abundance and estimated abundance on SIRV-set 4 data with different protocols and platforms.

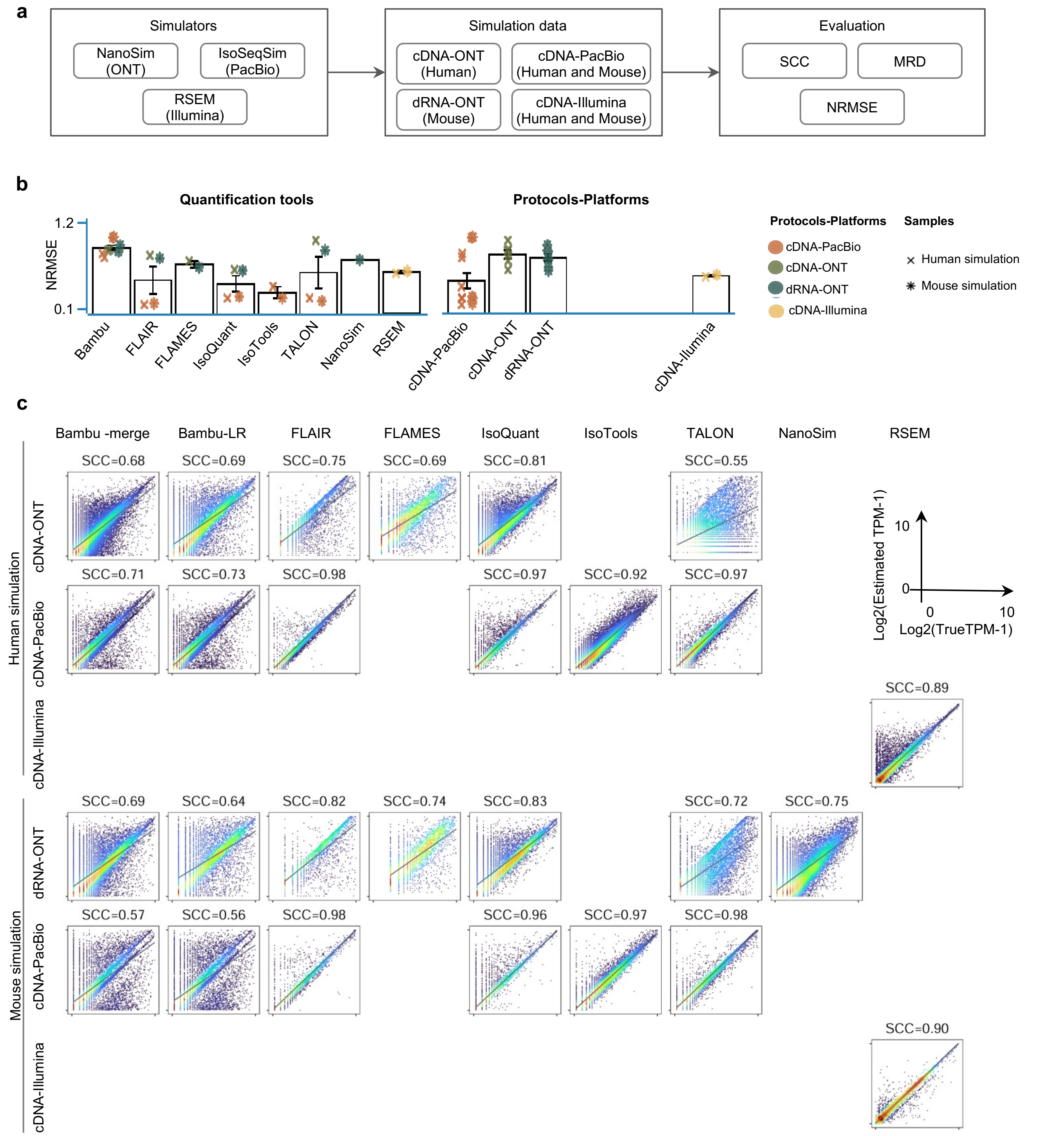

Supplementary Fig. 42. Performance evaluation on simulation data. a) The flow chart of simulation study. b) Evaluation results of NRMSE metric for different quantification tools and protocols-platforms. Bar plots are utilized to visualize the mean values of evaluation results across diverse datasets, with error bars indicating the standard deviation of NRMSE metric. c) Scatter plot of true abundance and estimated abundance on simulation data.

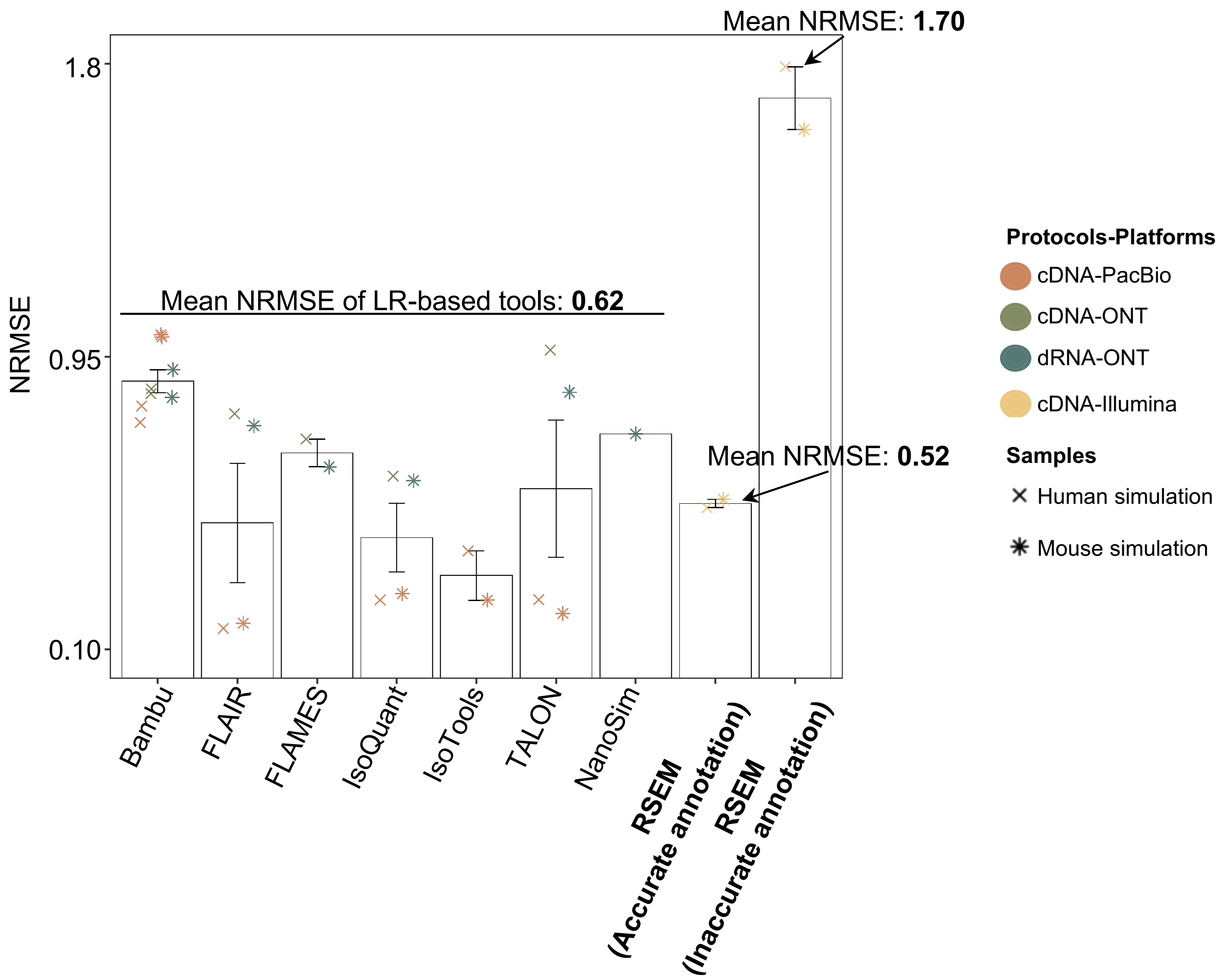

Supplementary Fig. 43. Impact of annotation accuracy on transcript quantification. We assessed the performance of RSEM and LR-based tools (Bambu, FLAIR, FLAMES, IsoQuant, IsoTools, TALON, and NanoSim) with different annotations. The NRMSE metric was used to evaluate their performance on simulated data for human and mouse. For LR-based tools, the transcript quantification annotations were derived from sample-specific annotations identified by the participant using long-read RNA-seq data. As for RSEM, we present quantification results based on two annotations: a completely accurate annotation (i.e., the ground truth transcripts generated by the simulation data) and an inaccurate annotation (i.e., the common GENCODE reference annotation, which contains numerous false negative and false positive transcripts specific to the sample). Bar plots are utilized to visualize the mean values of evaluation results across diverse datasets, with error bars indicating the standard deviation of NRMSE metric.

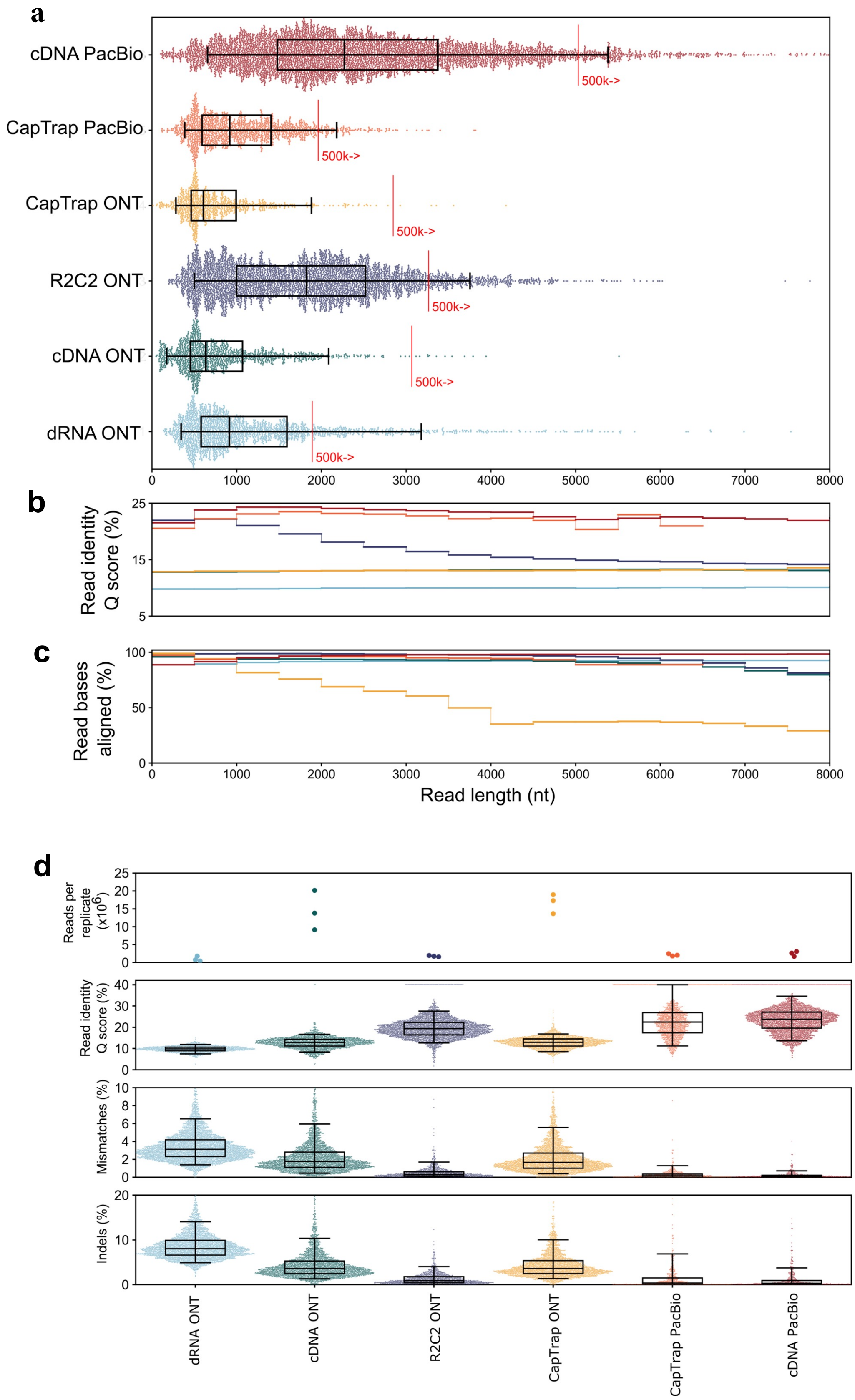

Supplementary Fig. 44. Read characteristics for the WTC-11 sample. a) Read lengths for the different library prep and technology combinations. The 500,000 longest reads for each library prep and technology combination fall to the right of a labeled red line overlapping each plot. b-c) The read identity and percent of aligned read bases of the different library prep and technology combinations is shown for reads of different length in 500nt bins. d) Read number for the three replicates, read identity, mismatch, and indel percentages for the different library prep and technology combinations are shown as vertical swarmplots. The box overlays for the swam plots percentiles are 5%, 25%, 50%, 75%, and 95%.

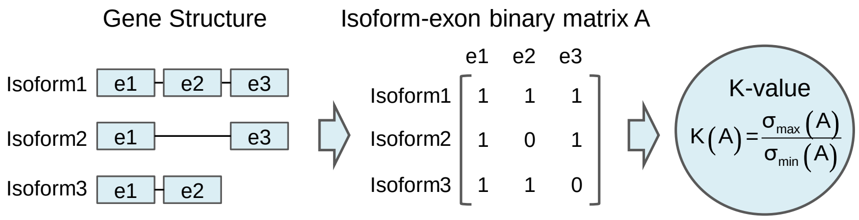

Supplementary Fig. 45. Description of K-value. A measure of the complexity of exon-isoform structures for each gene.

a)

Statistics	Before Polishing	After polishing
Number of Contigs (>= 5000 bp)	13,731	13,730
Total Length (>=50000 bp)	2,983,091,294	2,985,484,301
Number of Contigs (>= 1000 bp)	15,266	15,267
Number of Contigs (>= 10000 bp)	12,884	12,883
Number of N's per 100 kpb	0.12	0.09
Total Length	3,086,629,787	3,088,859,971
GC (%)	40.66	40.66
L75	4,050	4,050
Largest Contig	4,157,475	4,160,815
Total Length (>= 10000 bp)	3,075,586,967	3,077,805,048
Number of Contigs (>= 0 bp)	15,617	15,617
L50	1,801	1,801
Number of Contigs	15,602	15,602
Total Length (>= 1000 bp)	3,086,395,947	3,088,627,062
Total Length (>= 0 bp)	3,086,634,846	3,088,865,031
Total Length (>= 5000 bp)	3,081,880,108	3,084,103,900
Total Length (>= 25000 bp)	3,045,366,563	3,047,614,467
Number of Contigs (>= 50000 bp)	9,355	9,359
N50	488,759	488,962
N75	239,915	239,985
Number of Contigs (>=25000 bp)	11,062	11,063

b)

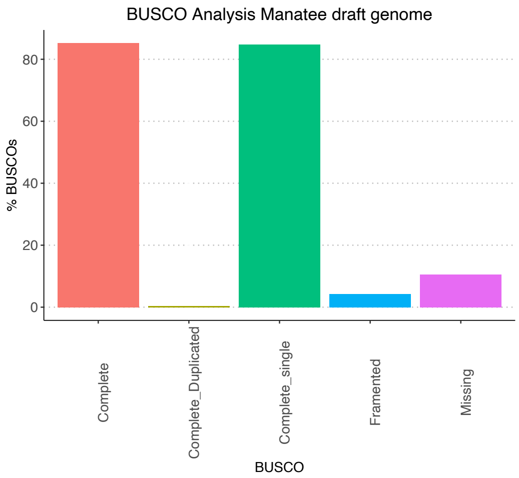

Supplementary Fig. 46. Manatee genome assembly statistics. a Nanopore reads were used to obtain a draft genome of the Floridian manatee with Flye. The resulting assembly was polished with existing Illumina reads using Pilon. b BUSCO completeness.

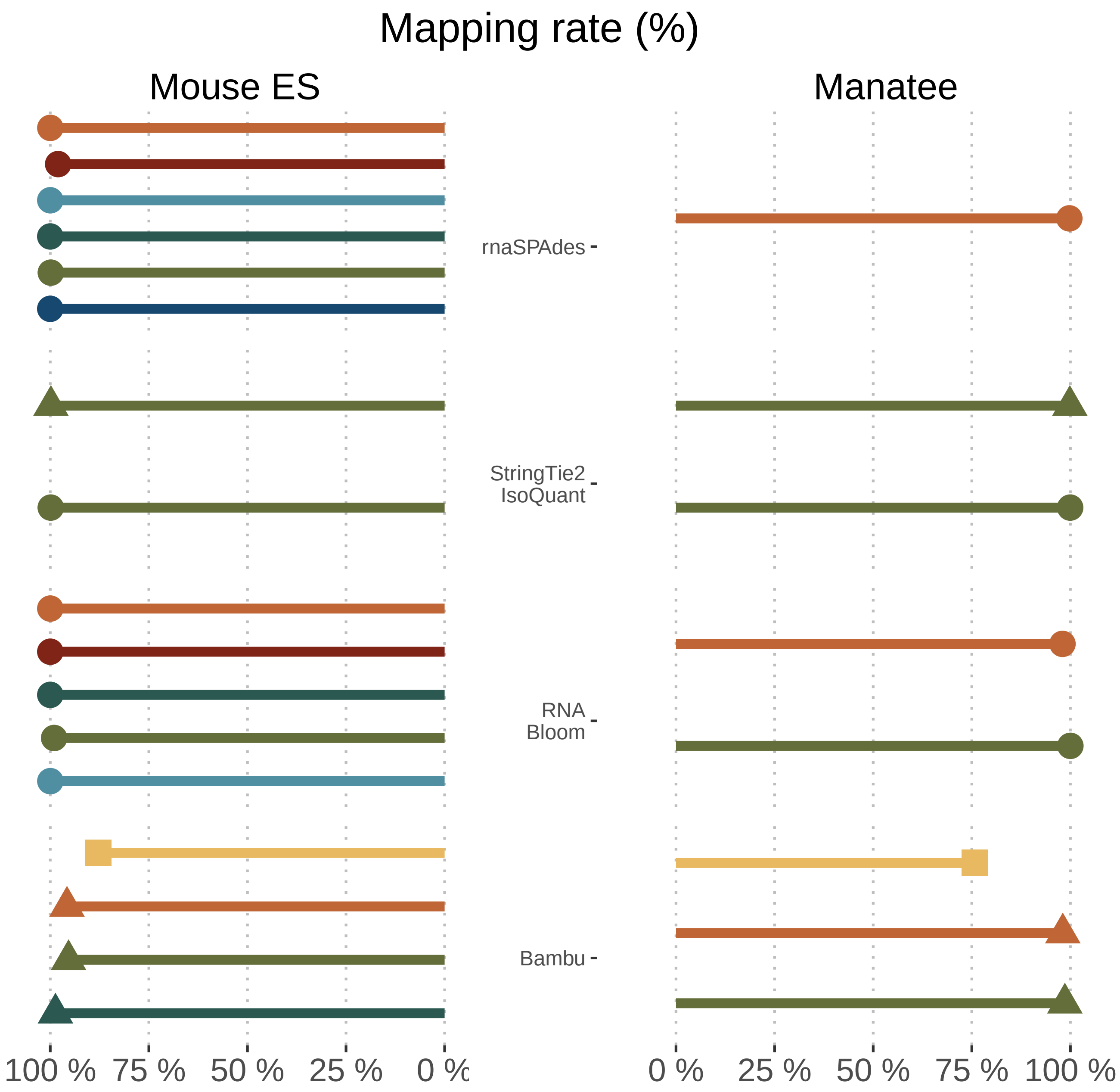

Supplementary Fig. 47. Mapping rate of transcript detected by Challenge 3 submissions.

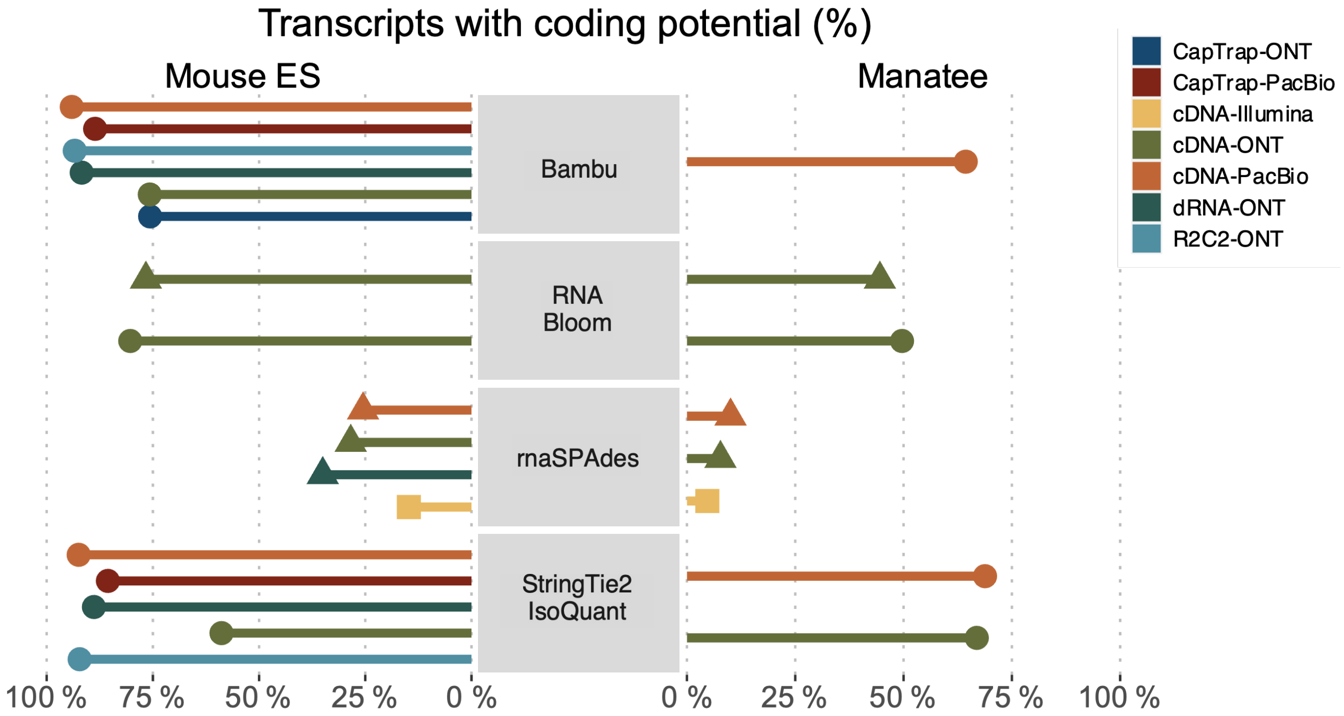

Supplementary Fig. 48. Coding potential of transcripts detected by Challenge 3 submissions.

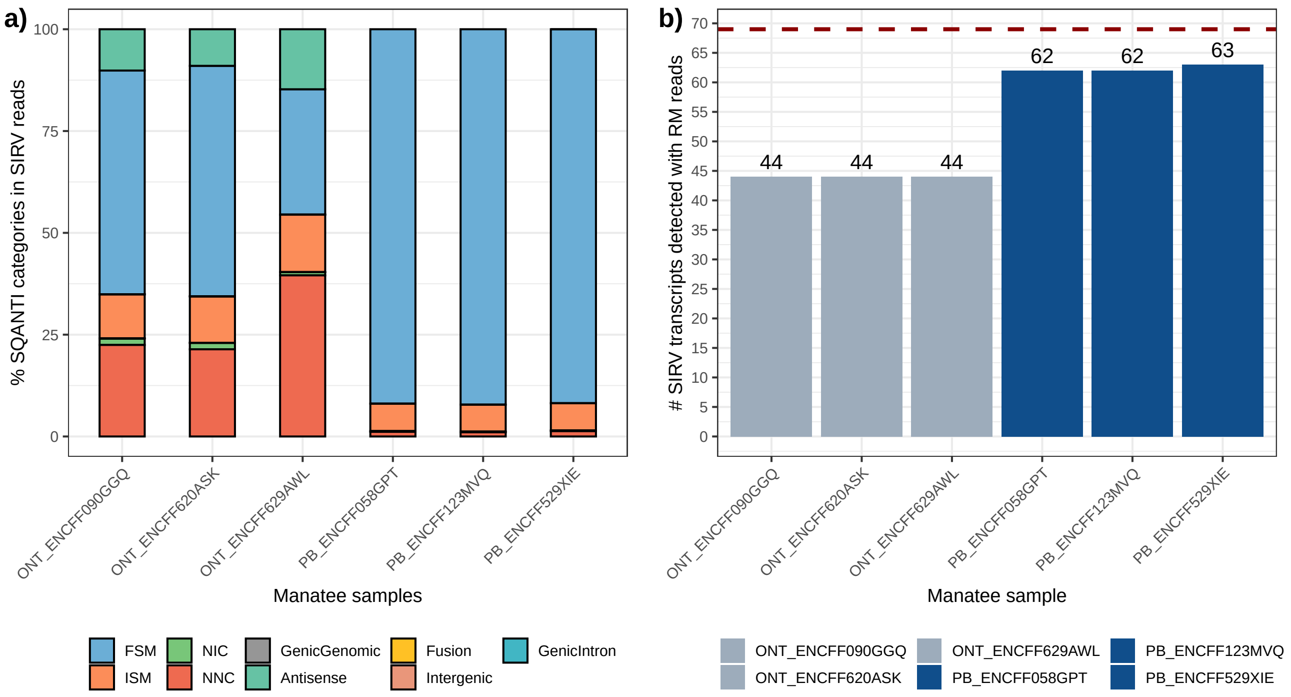

Supplementary Fig. 49. SQANTI3 analysis of SIRV reads in manatee samples. a) SQANTI3 categories for reads mapping to SIRVs in cDNA-PacBio and cDNA-ONT replicates. b) Number of SIRV transcripts with at least one Reference Match (RM) read in cDNA-PacBio and cDNA-ONT replicates.

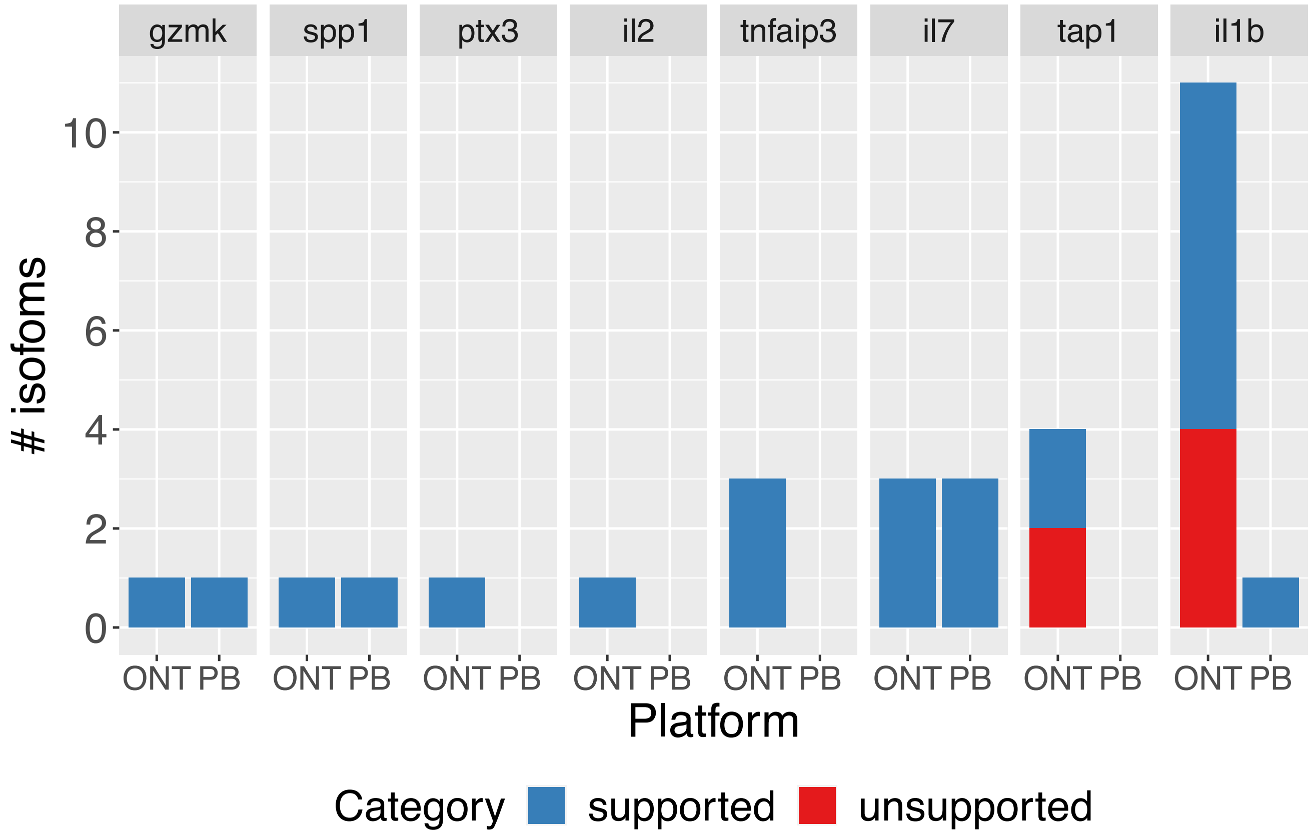

Supplementary Fig. 50. PCR validation results for manatee isoforms for seven target genes (data shown in Figure 5l) broken down by the platform (ONT or PacBio) underlying the pipelines that led to the identification of the isoform.

a chr1:63,405,342-63,413,931

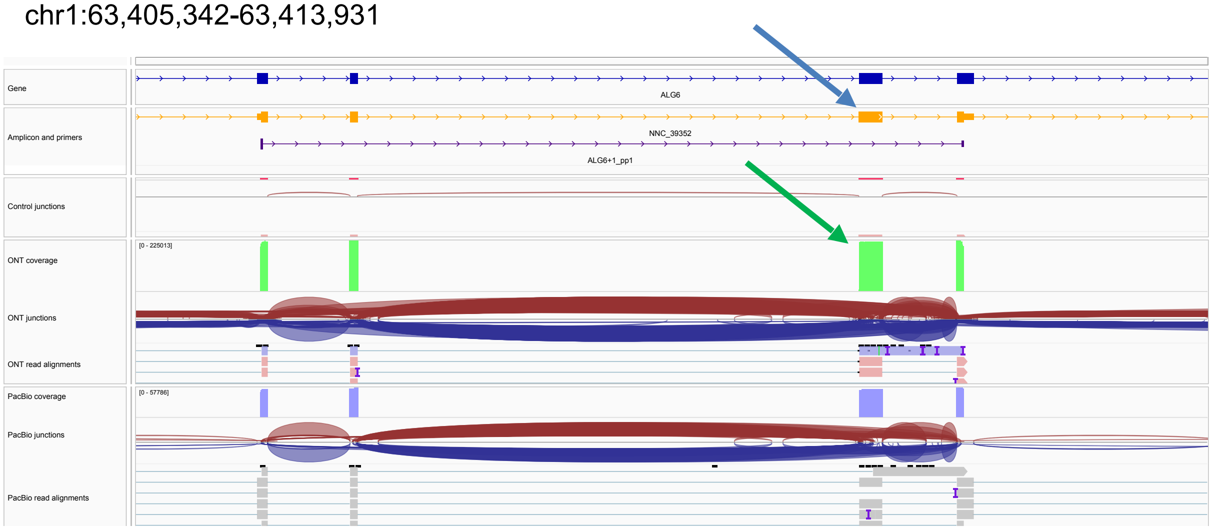

b chr1:63,411,123-63,411,162

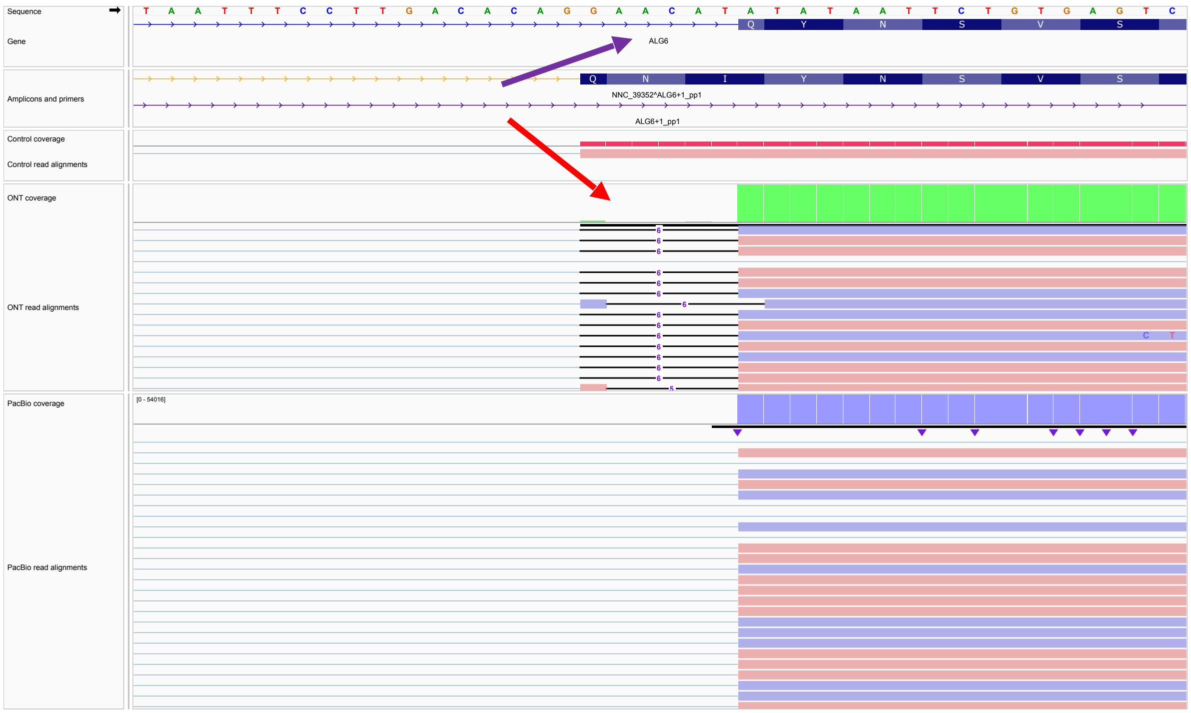

Supplementary Fig. 51. Validation of ALG6 U12 Intron with WTC11 Reads. In panel (a), a novel transcript model, NCC_39352 (blue arrow), appears to corroborate the exon within the ALG6 GENCODE annotation. The mapped amplicon in the control junction tracks provides evidence of the preceding intron. The green arrow indicates the ONT and PacBio read alignment coverage over the exon, but the junction tracks shows a lack of support for the splice junction at the exon's 5' end. In panel (b), GENCODE's annotation of a rare U12 GT-AT intron (purple arrow), which is unsupported by minimap2. Instead, minimap2 forces a GT-AG intron by reporting a six-base deletion in the reference genome (red arrow). As all pipelines relied on minimap2, correct annotation of this transcript was unattainable, illustrating the challenges difficult-to-align regions can pose to annotation with long-read transcripts.

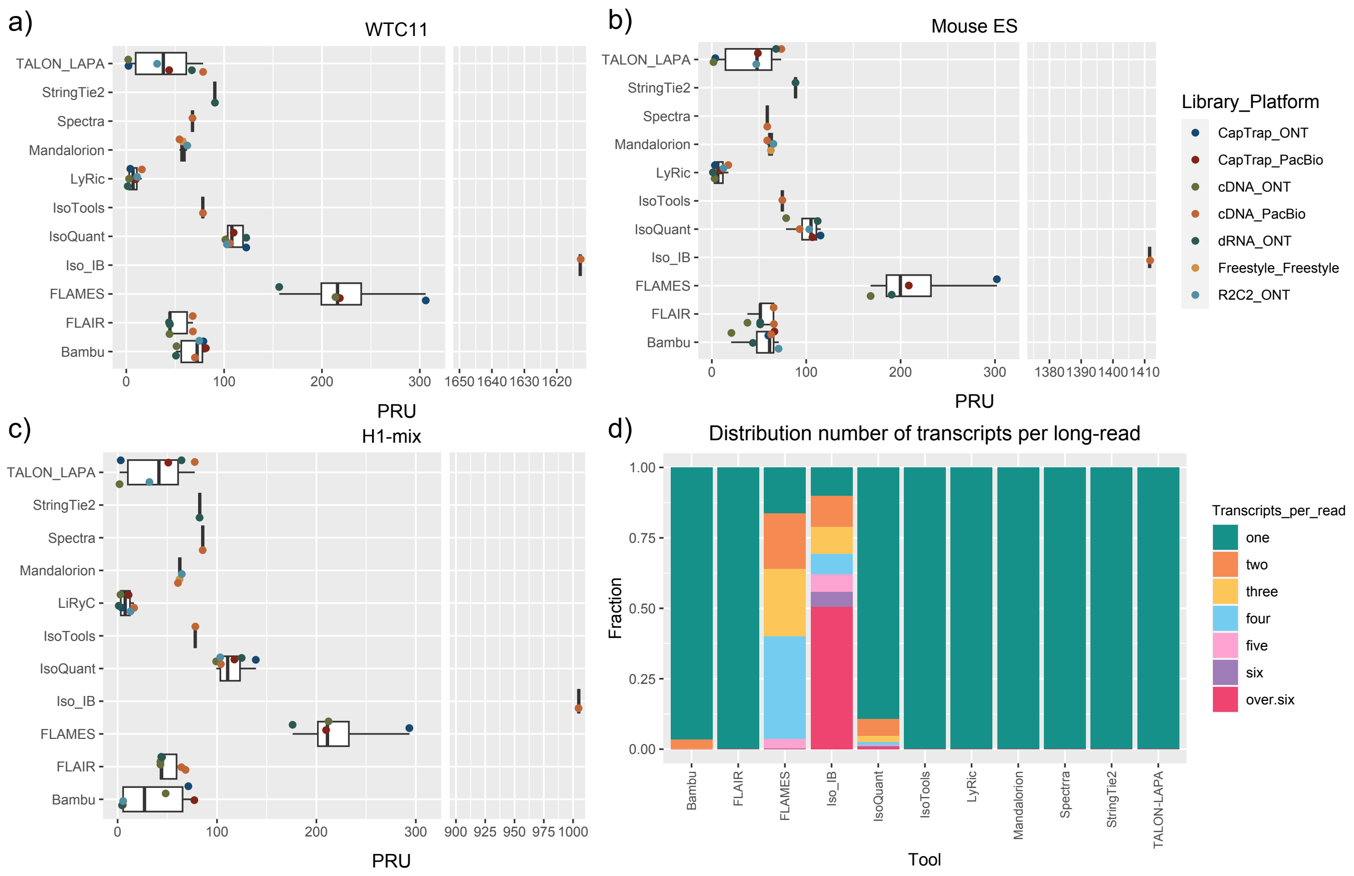

Supplementary Fig. 52. Read usage by analysis tool. a-c) The Percentage of Reads Used (PRU) is calculated as the fraction between the number of reads in transcript models provided in the submission of each pipelines and the number of available reads in the dataset. Values ¿ 100 indicate the same read is assigned to more than one transcript model. Values ¡ 100 indicate that not all available reads were used to predict transcript models. d) Distribution of the number of transcripts assigned to each long-read in the submitted reads2transcripts files. Values are aggregated for all submissions of the same tool.

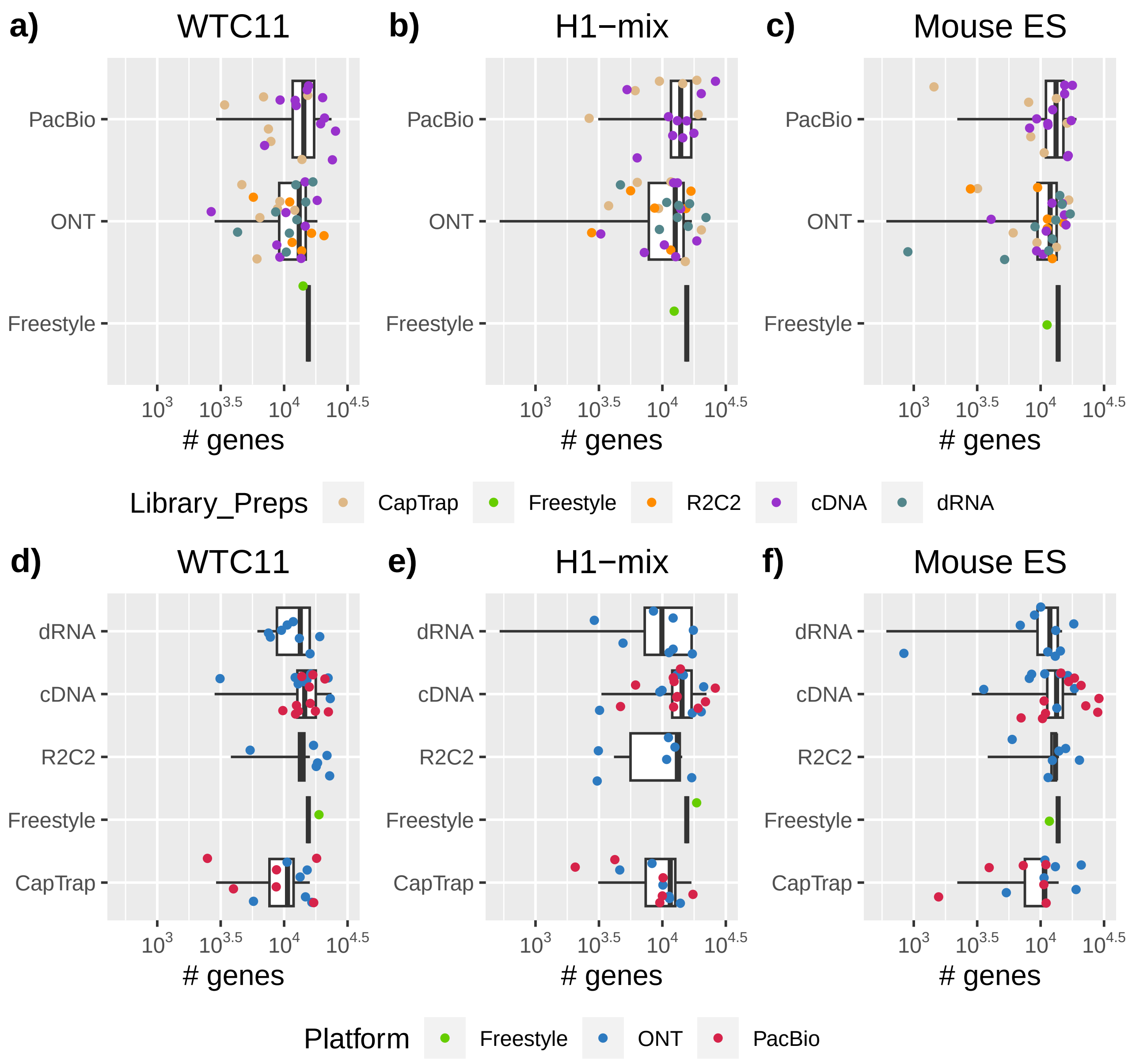

Supplementary Fig. 53. Number of detected genes per Platform and Library Preparation. a-c) Platform, d-f) Library Preparation.

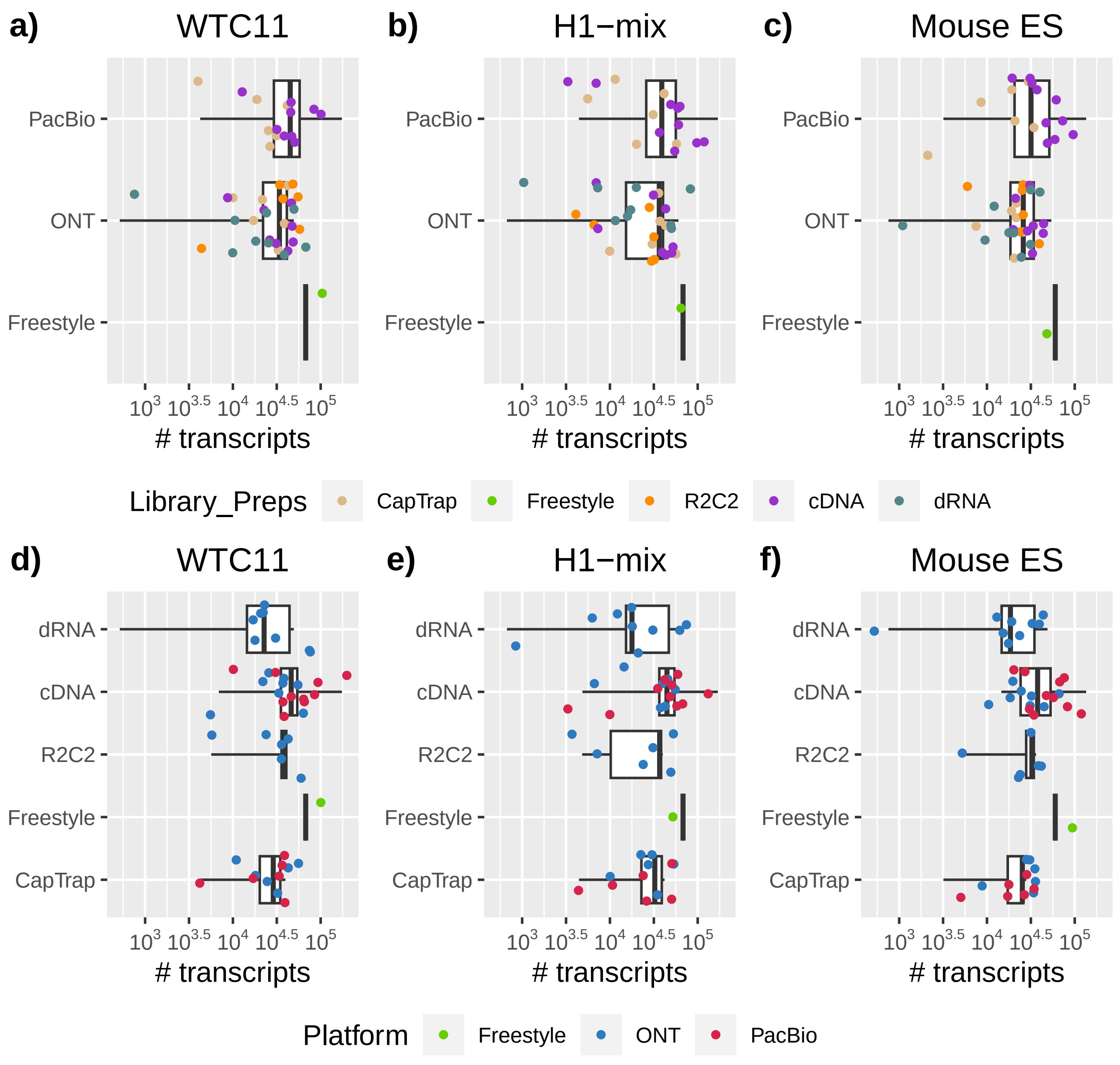

Supplementary Fig. 54. Number of detected transcripts per Platform and Library Preparation.

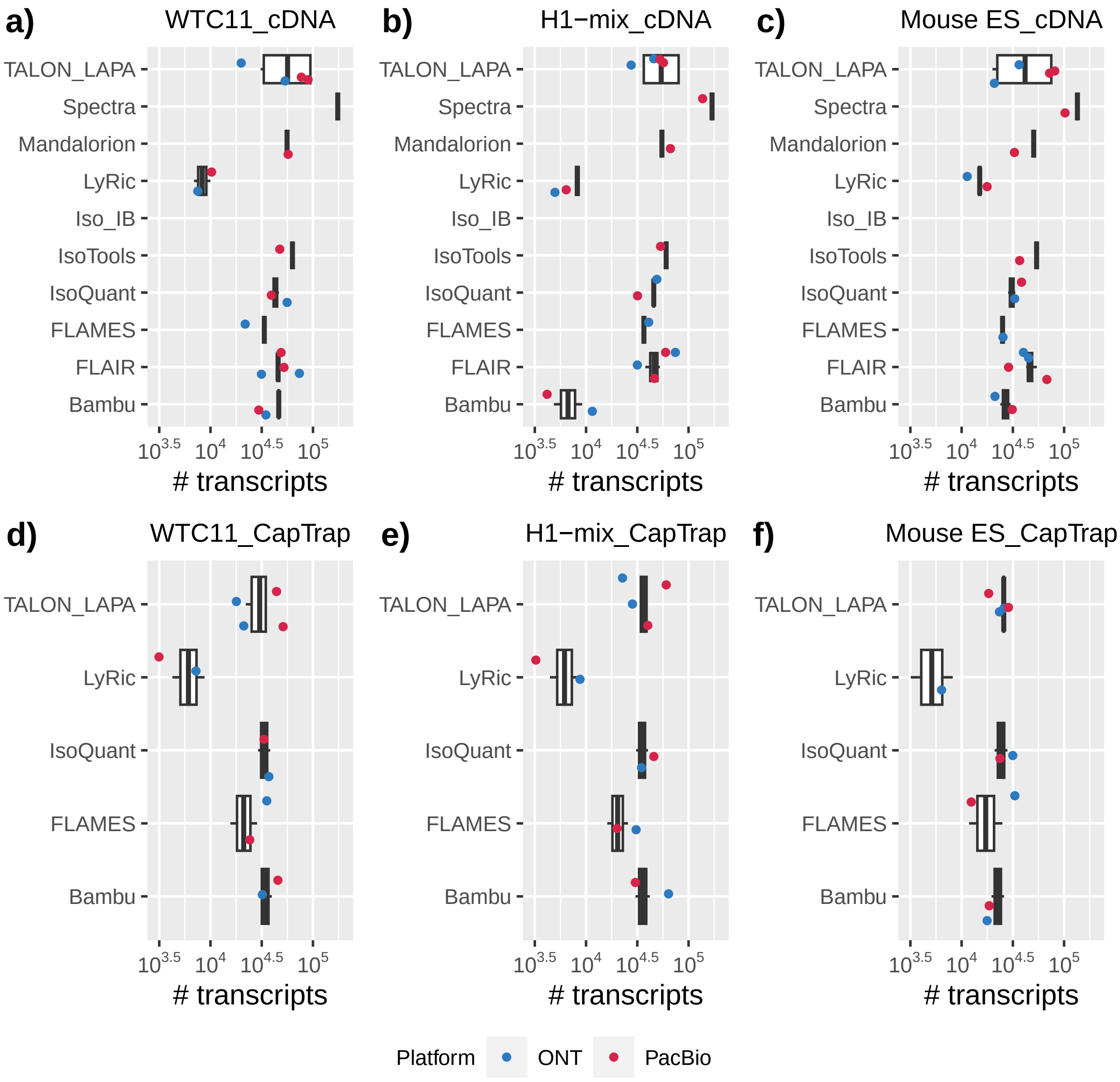

Supplementary Fig. 55. Number of detected transcripts in cDNA and CapTrap libraries. a-c) cDNA, d-f) CapTrap.

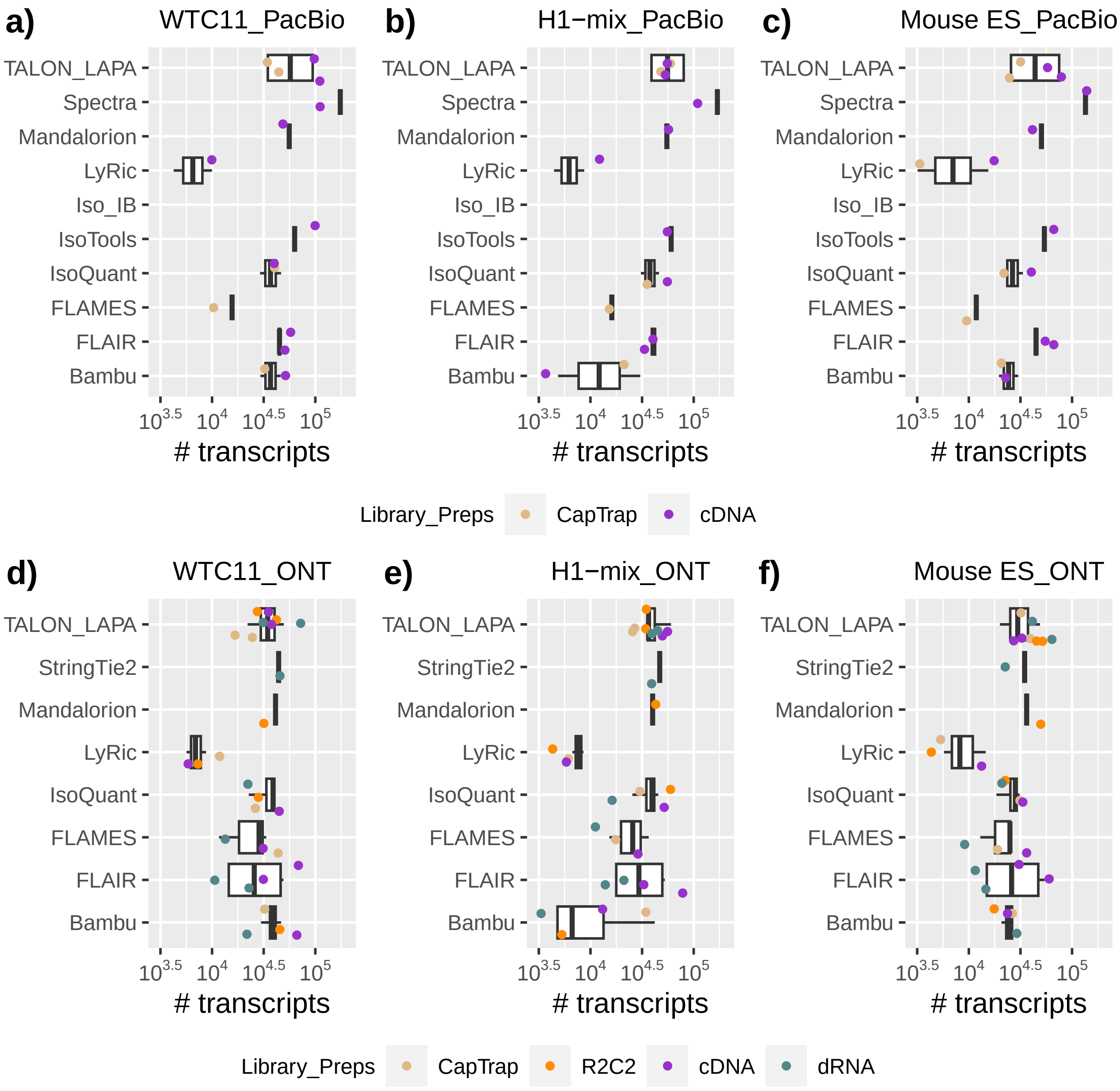

Supplementary Fig. 56. Number of detected transcripts in PacBio and Nanopore platforms. a-c) PacBio, d-f) Nanopore.

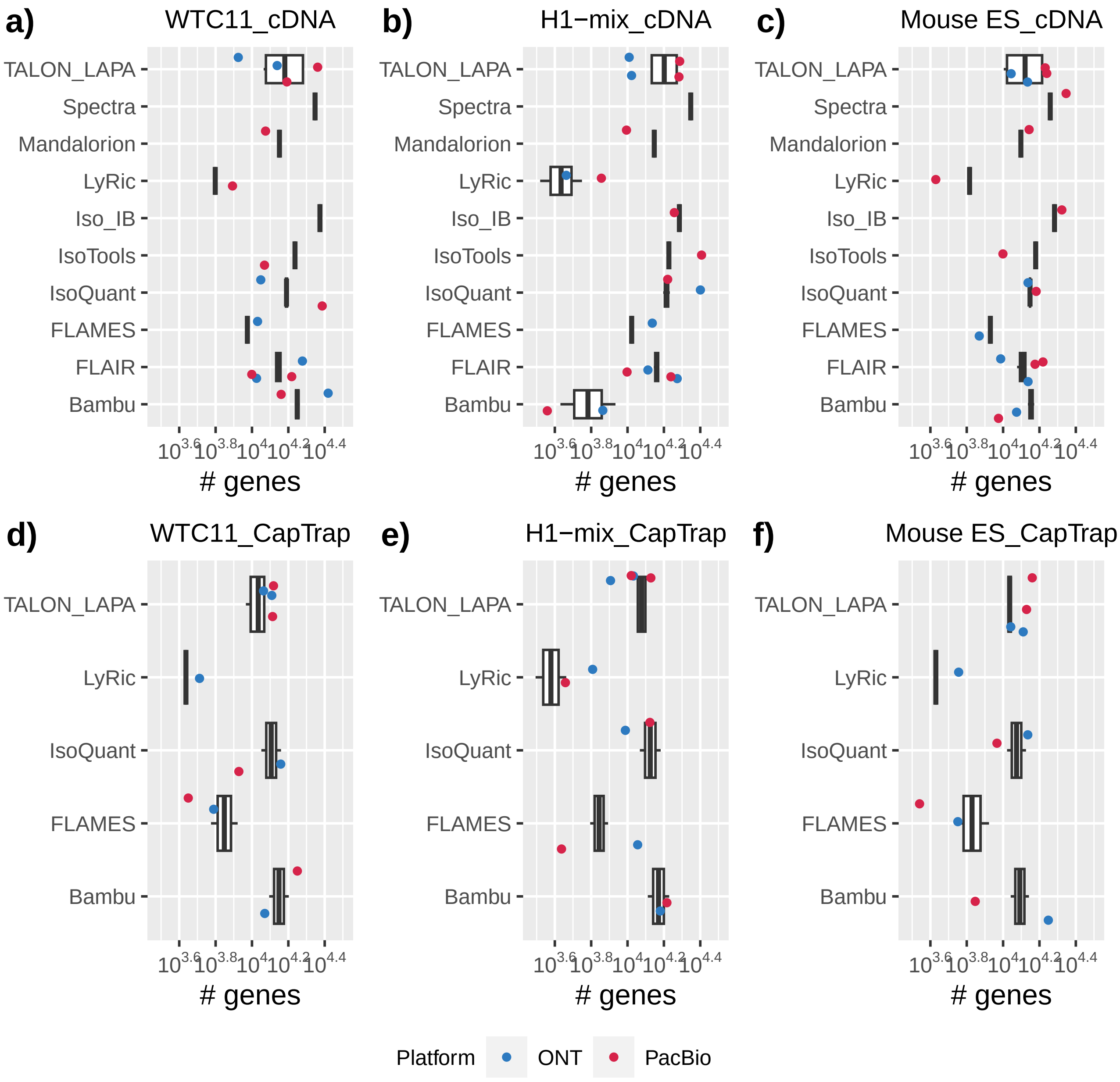

Supplementary Fig. 57. Number of detected genes in cDNA and CapTrap libraries. a-c) cDNA, d-f) CapTrap.

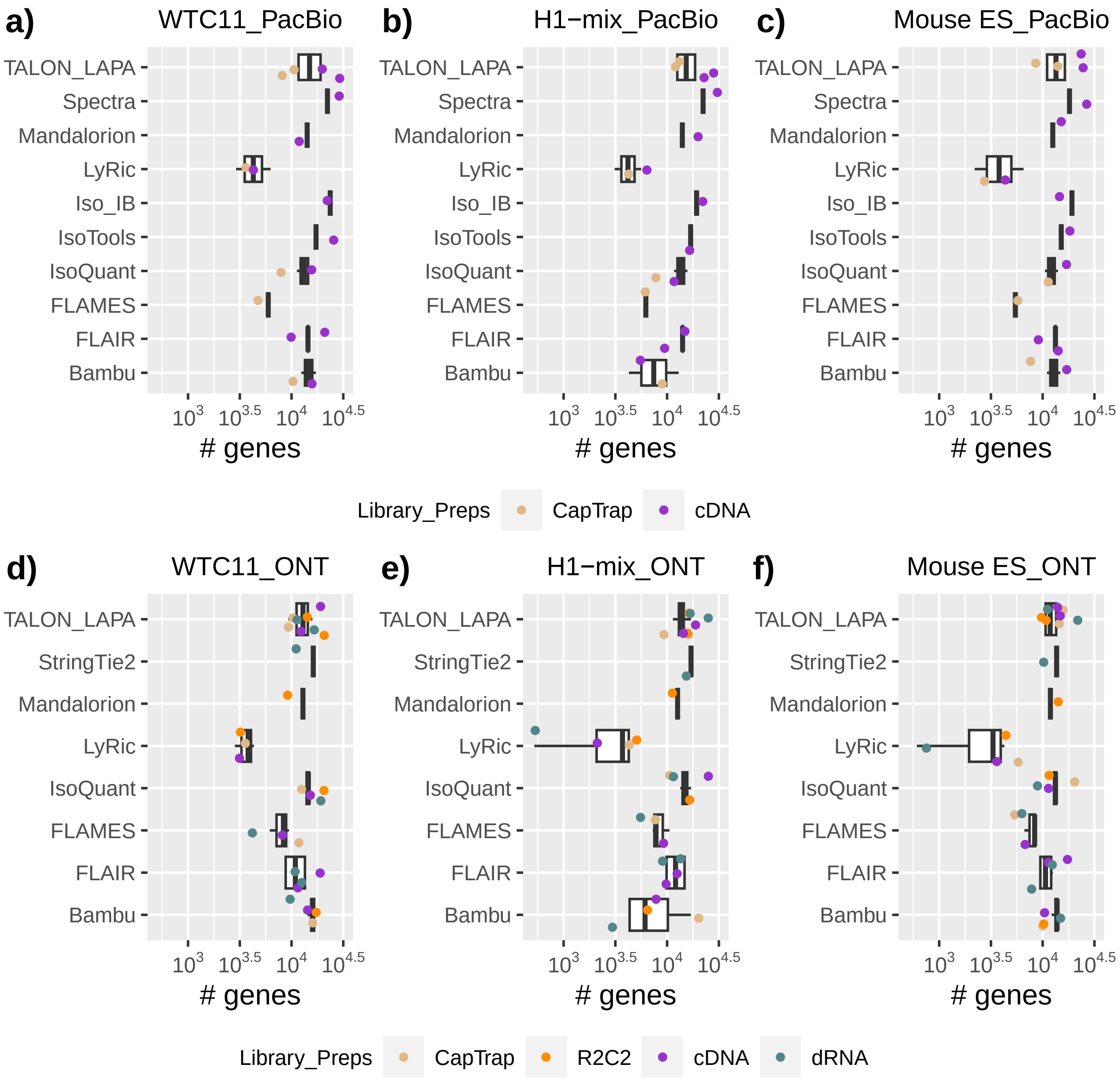

Supplementary Fig. 58. Number of detected genes in PacBio and Nanopore platforms. a-c) PacBio, d-f) Nanopore.

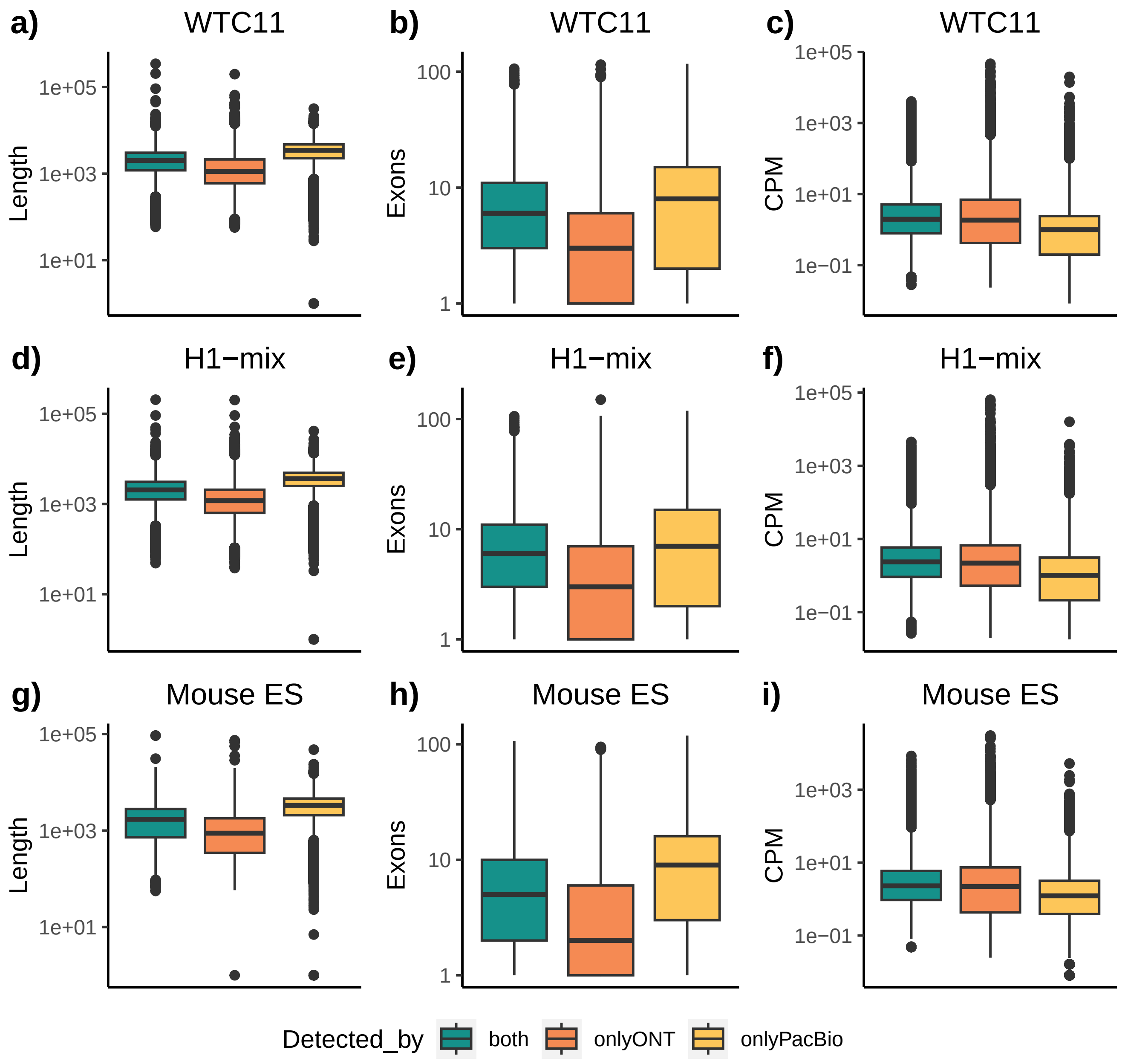

Supplementary Fig. 59. Properties of detected transcripts by library preparation. a,d,g) Length distribution. b,e,h) Exon number distribution. c,f,i) Counts per million

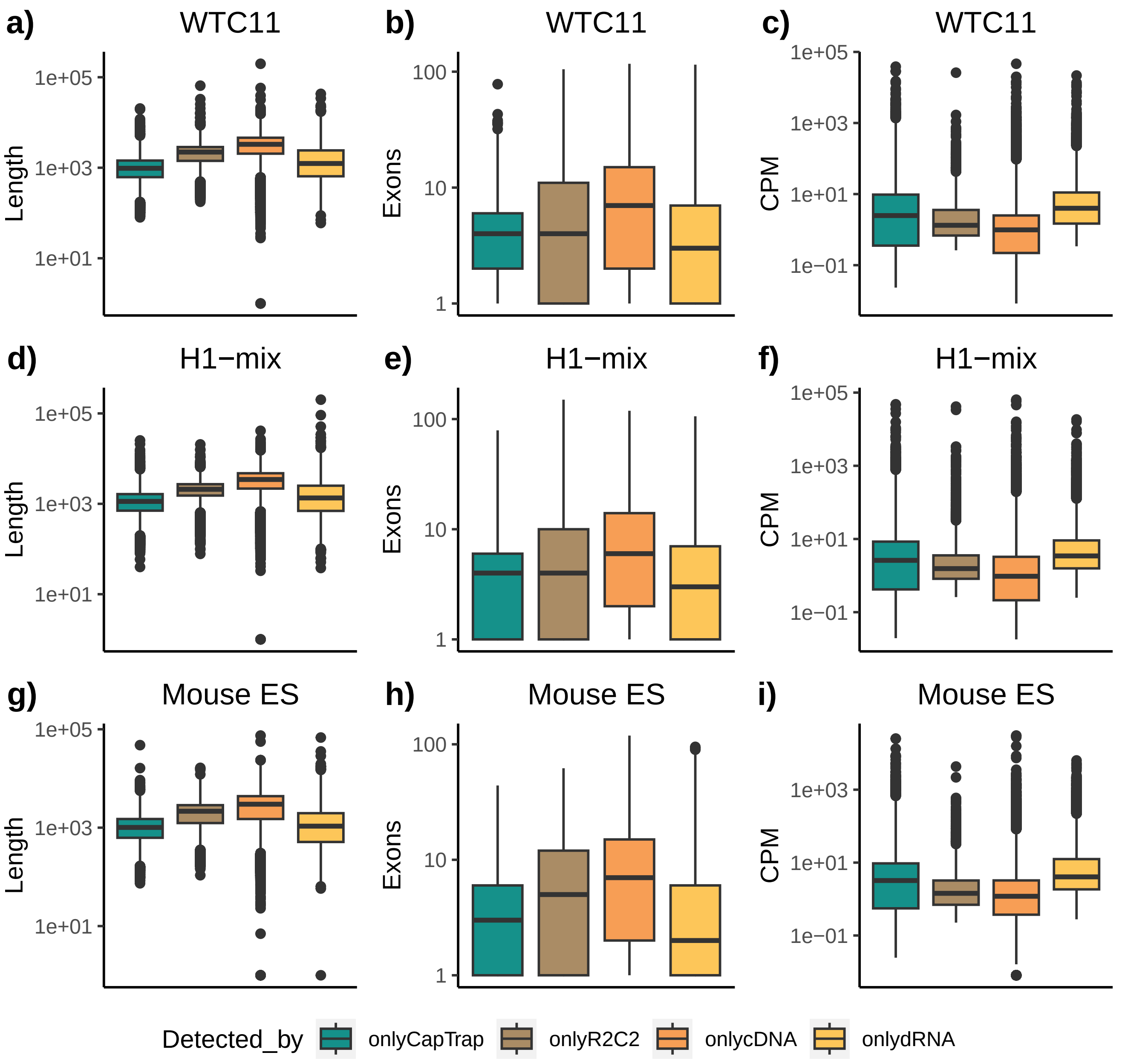

Supplementary Fig. 60. Properties of detected transcripts by platform. a,d,g) Length distribution. b,e,h) Exon number distribution. c,f,i) Counts per million

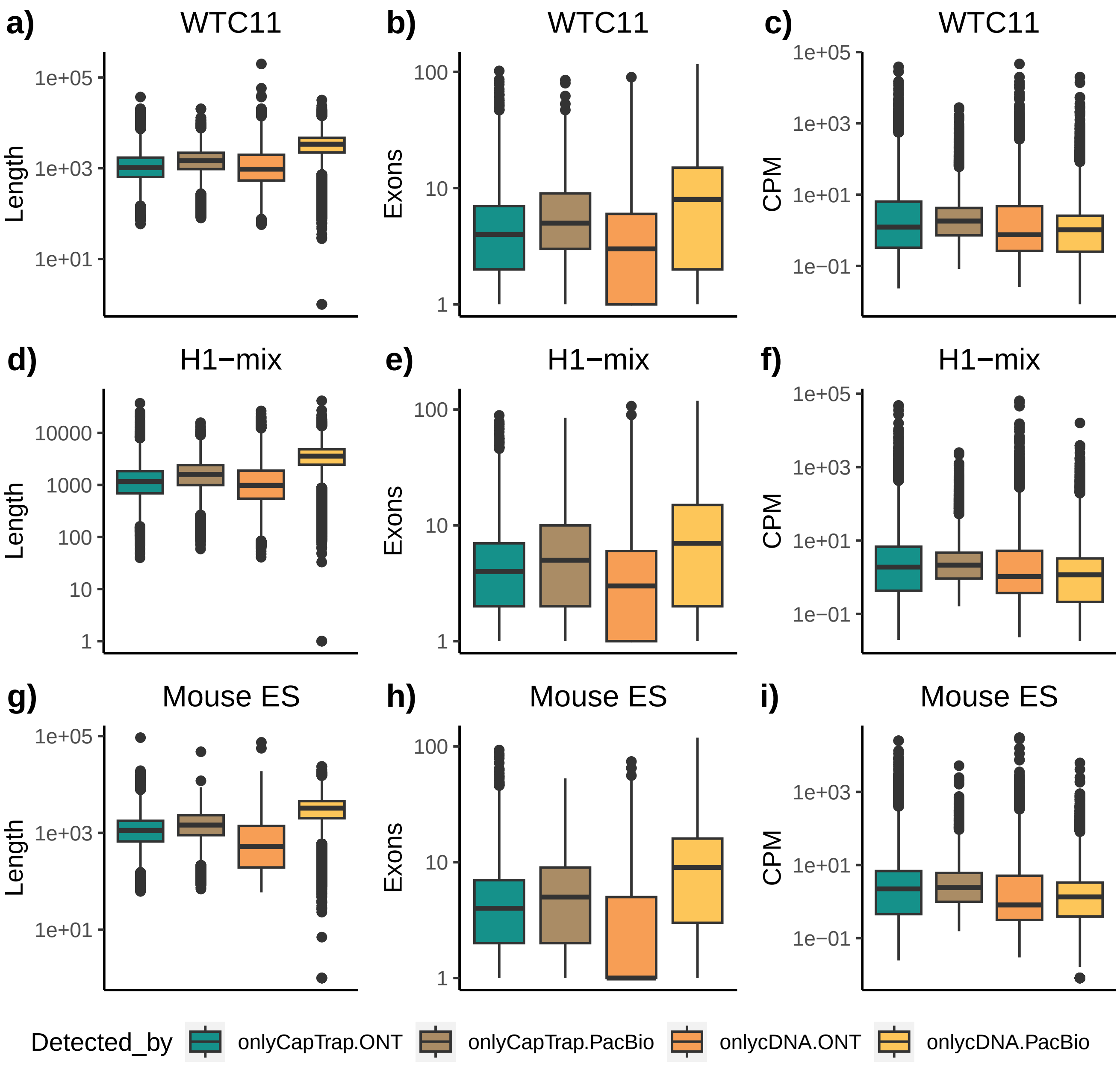

Supplementary Fig. 61. Properties of detected transcripts by experimental protocol. a,d,g) Length distribution. b,e,h) Exon number distribution. c,f,i) Counts per million

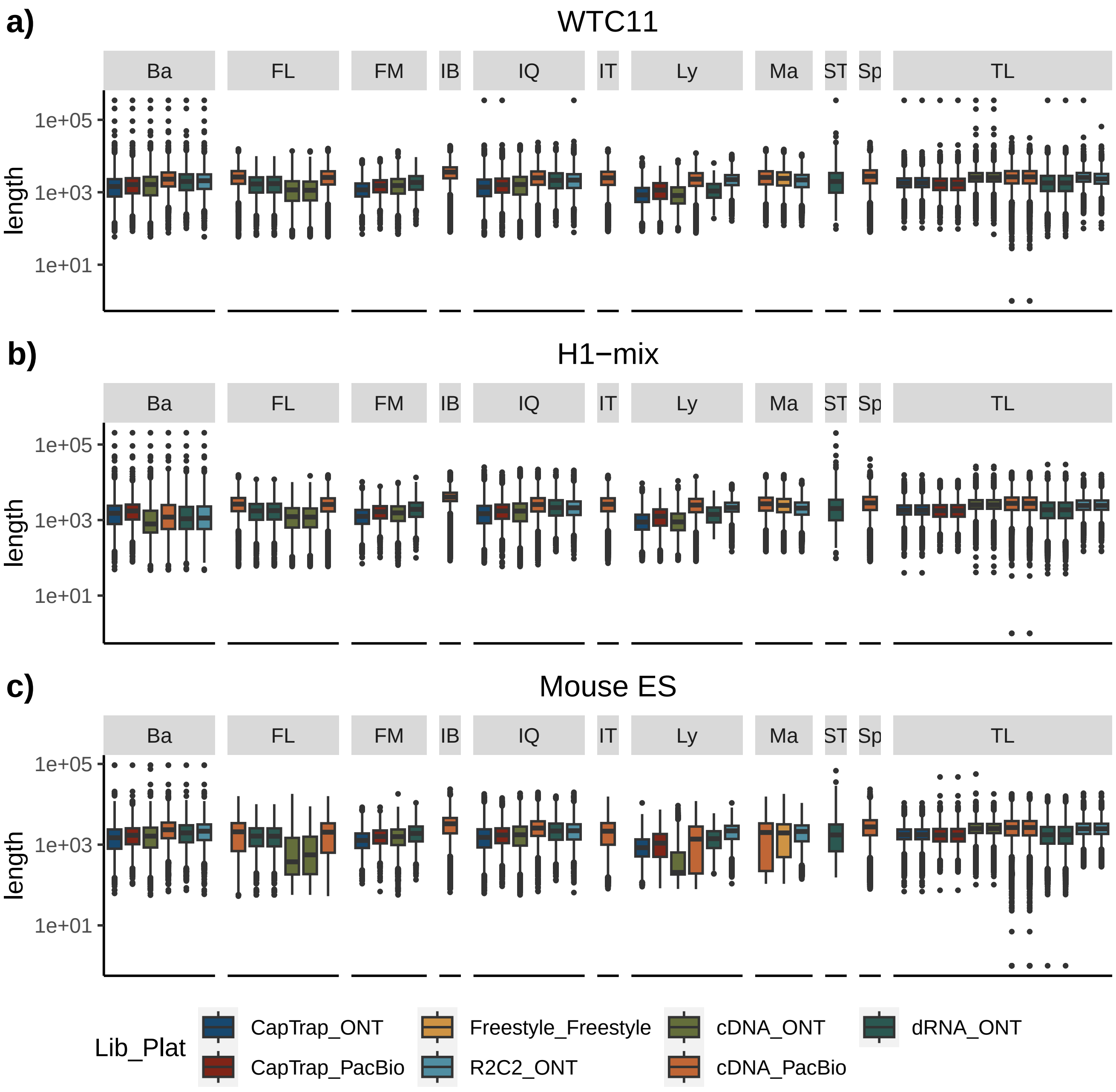

Supplementary Fig. 62. Distribution of transcript length by analysis tool.

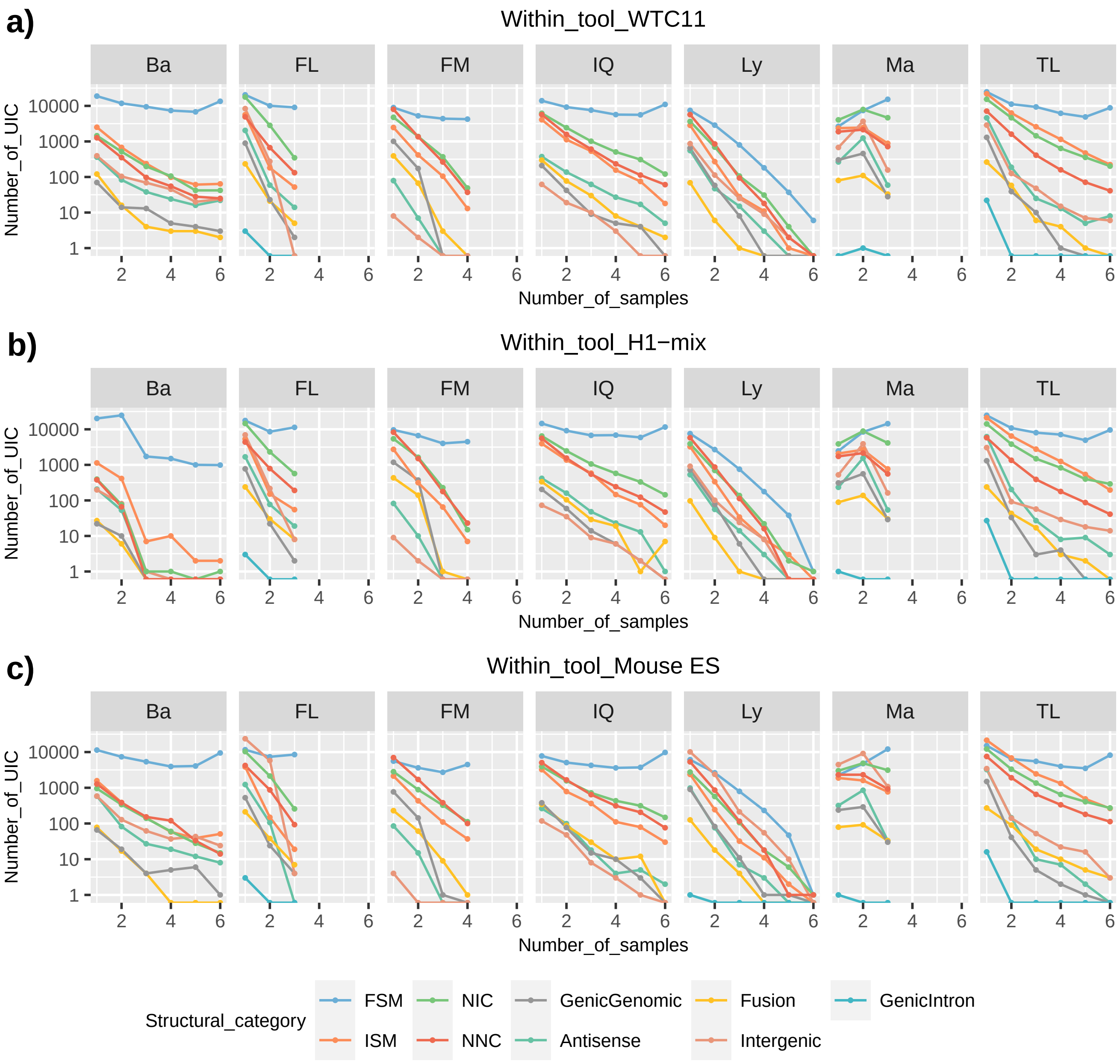

Supplementary Fig. 63. Number of UIC consistently detected by a tool across samples. a) WTC11, c) H1-mix, c) Mouse ES

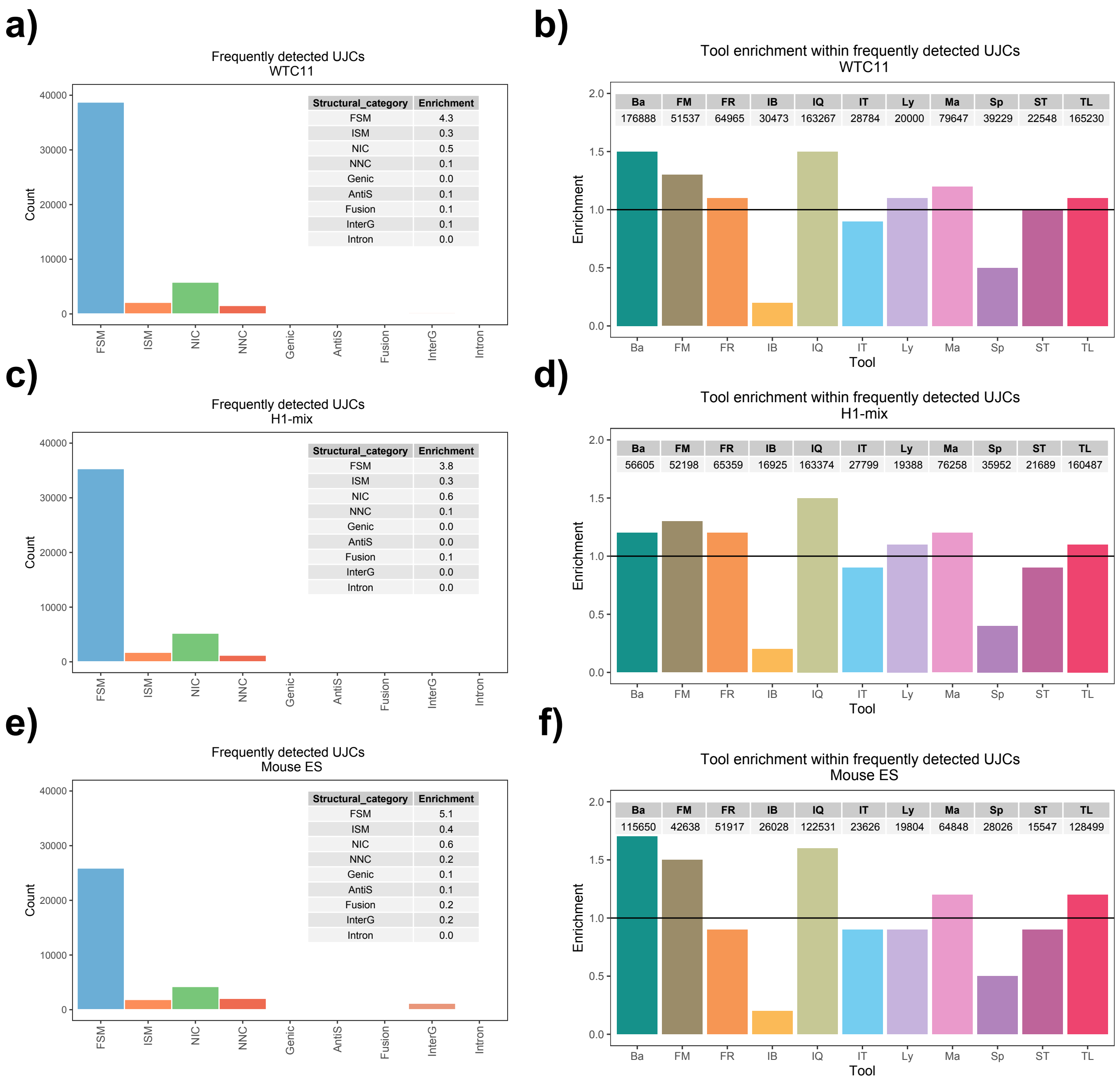

Supplementary Fig. 64. Characterization of frequently detected UICs (FDU). a,c,e) Structural category distribution of FDU. The table indicates the fold enrichment of each structural category within the frequently detected transcripts respect to their global count. b,d,f) Tools identifying FDU. The graph shows the enrichment in the number FDU found by a tool with respect to their global number of reported transcripts. The table reports the total number of FDU detected by the tool.

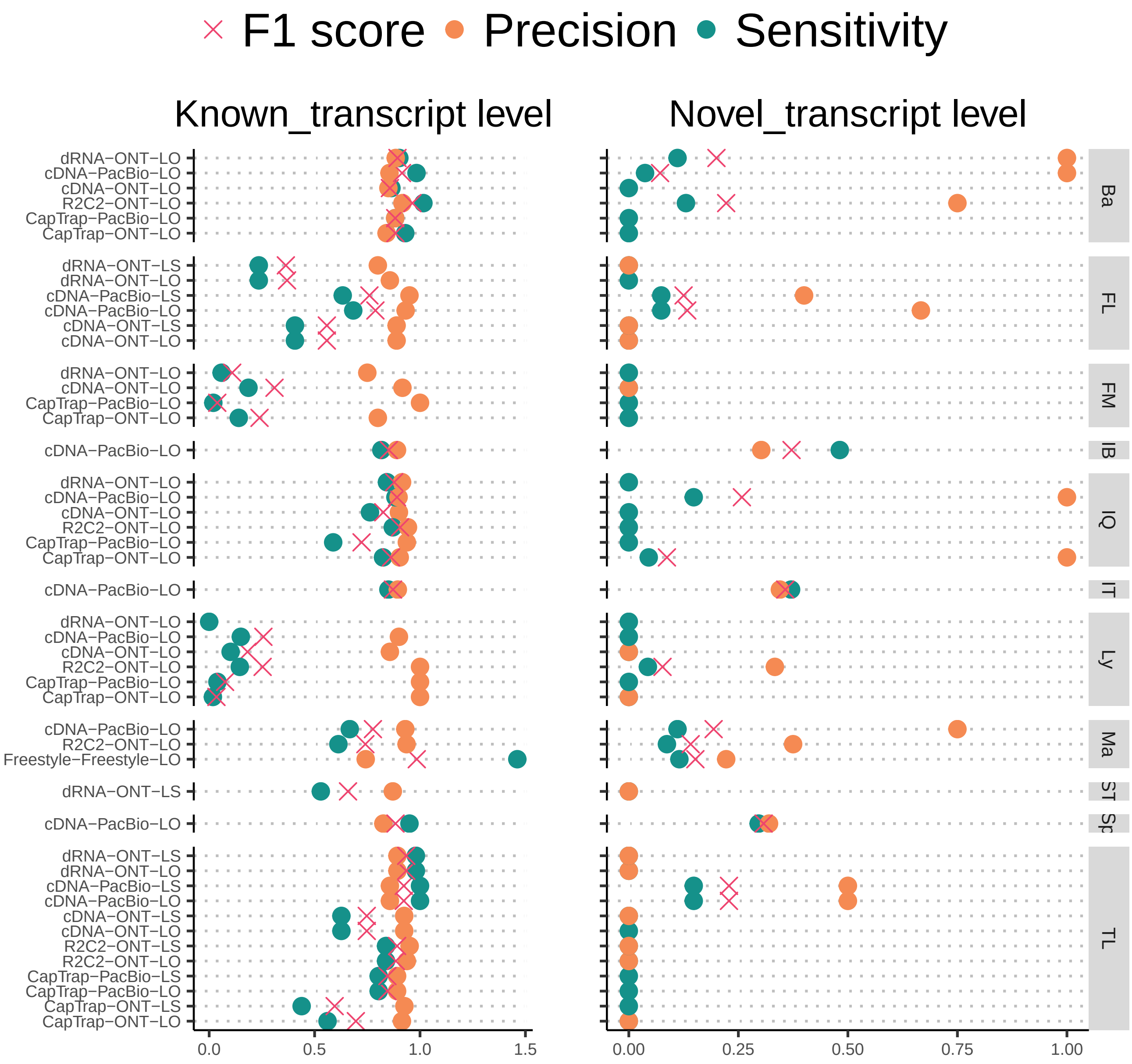

Supplementary Fig. 65. Performance on GENCODE manually curated data. Curated transcripts selected to be present in at least two experimental datasets. Ba: Bambu, FM: Flames, FL: FLAIR, IQ: IsoQuant, IT: IsoTools, IB: Iso_IB, Ly: LyRic, Ma: Mandalorion, TL: TALON-LAPA, Sp: Spectra, ST: StringTie2.

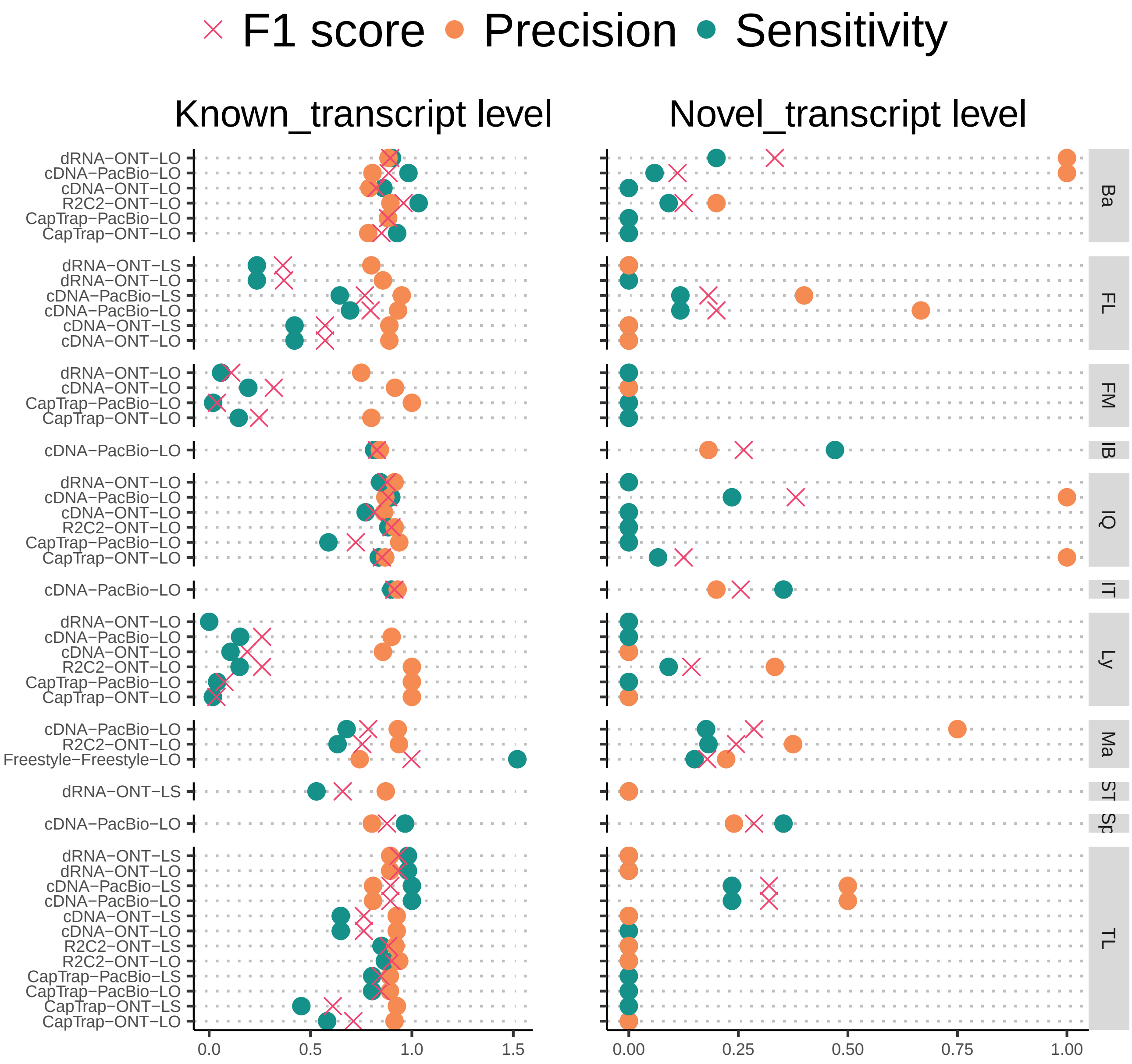

Supplementary Fig. 66. Performance on GENCODE manually curated data. The ground truth is the set of manually annotated transcripts with more than two reads. Ba: Bambu, FM: Flames, FL: FLAIR, IQ: IsoQuant, IT: IsoTools, IB: Iso_IB, Ly: LyRic, Ma: Mandalorion, TL: TALON-LAPA, Sp: Spectra, ST: StringTie2.

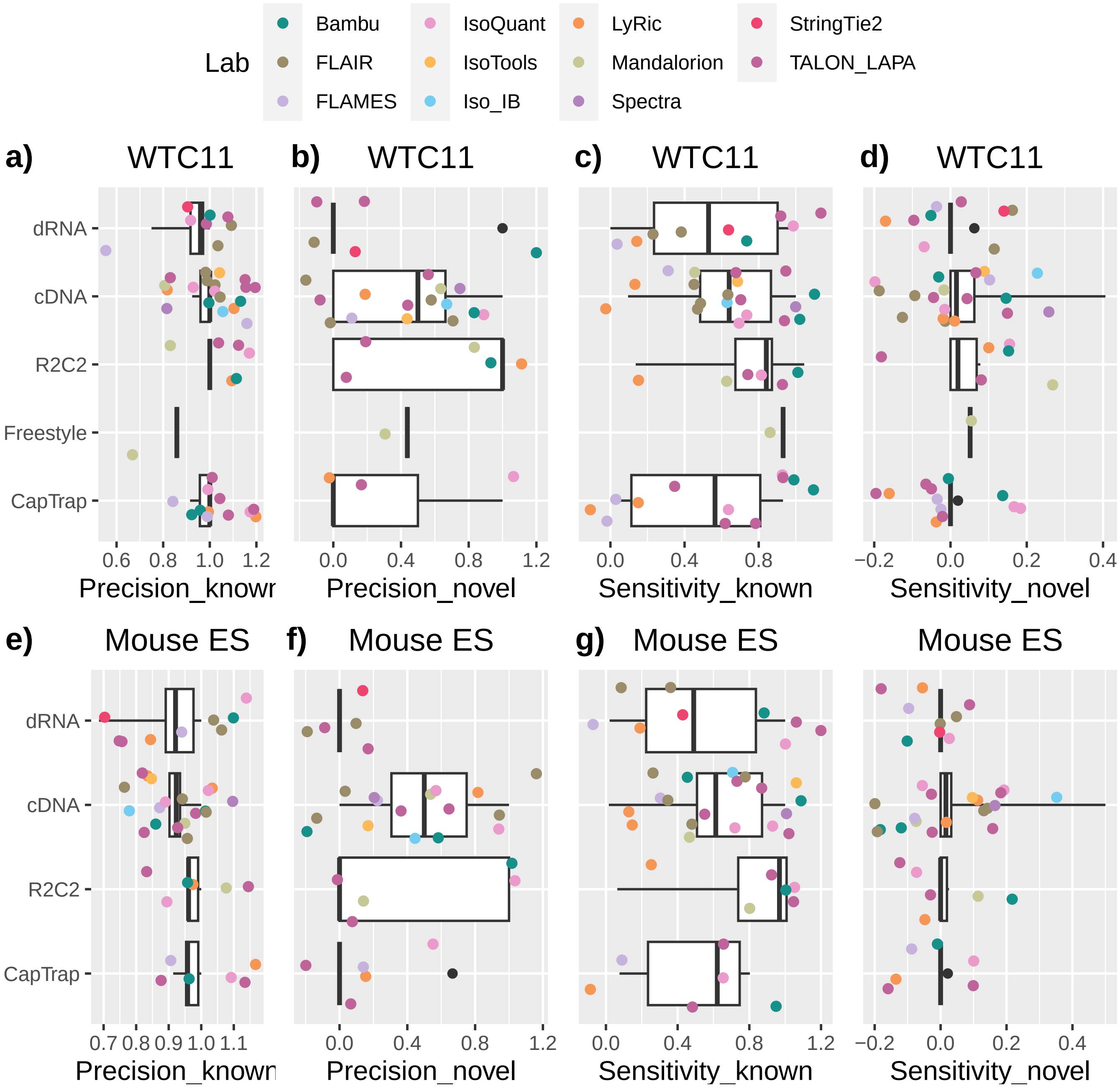

Supplementary Fig. 67. Performance on GENCODE manually curated data by Library Preparation.

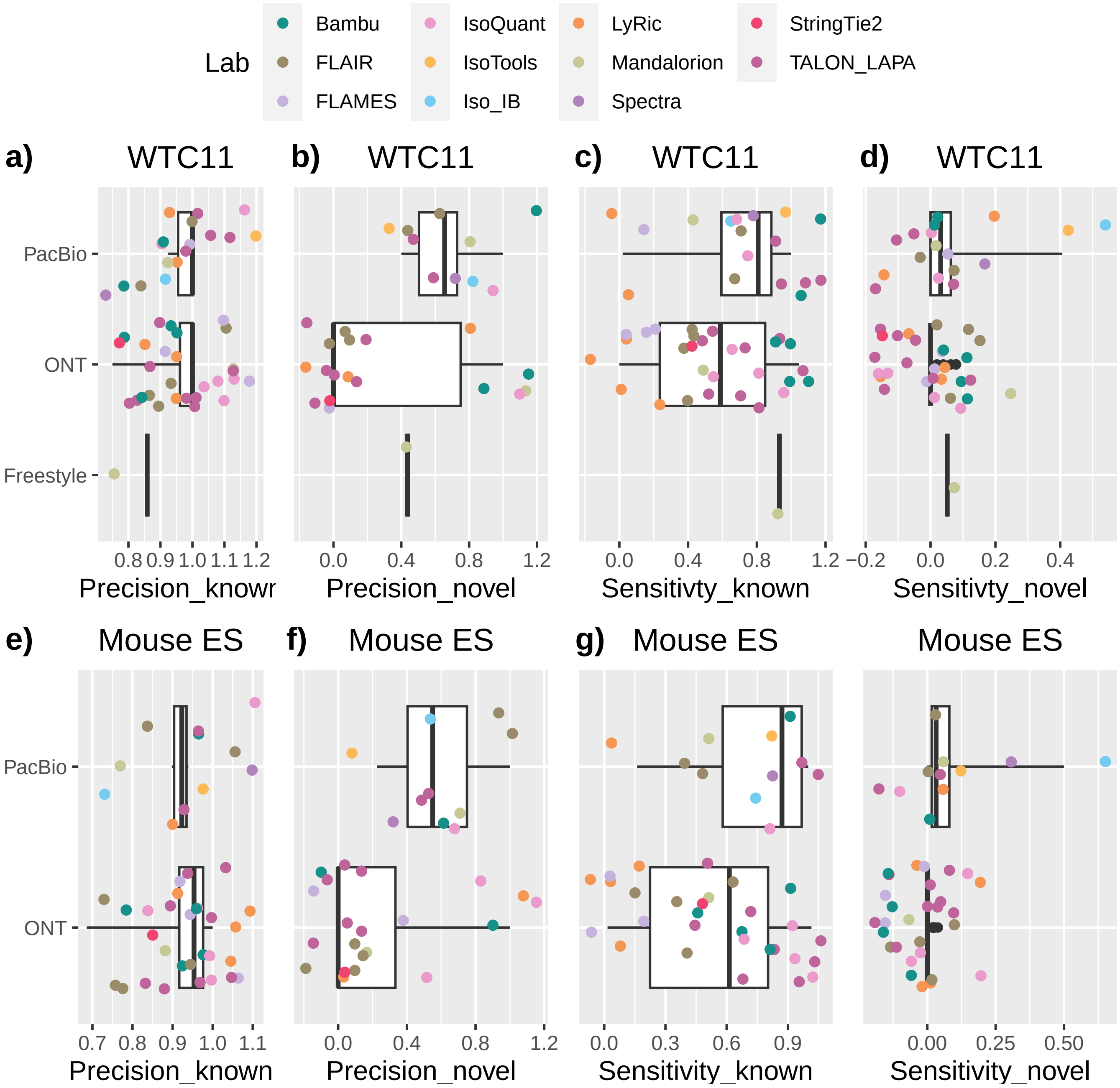

Supplementary Fig. 68. Performance on GENCODE manually curated data by Platform.

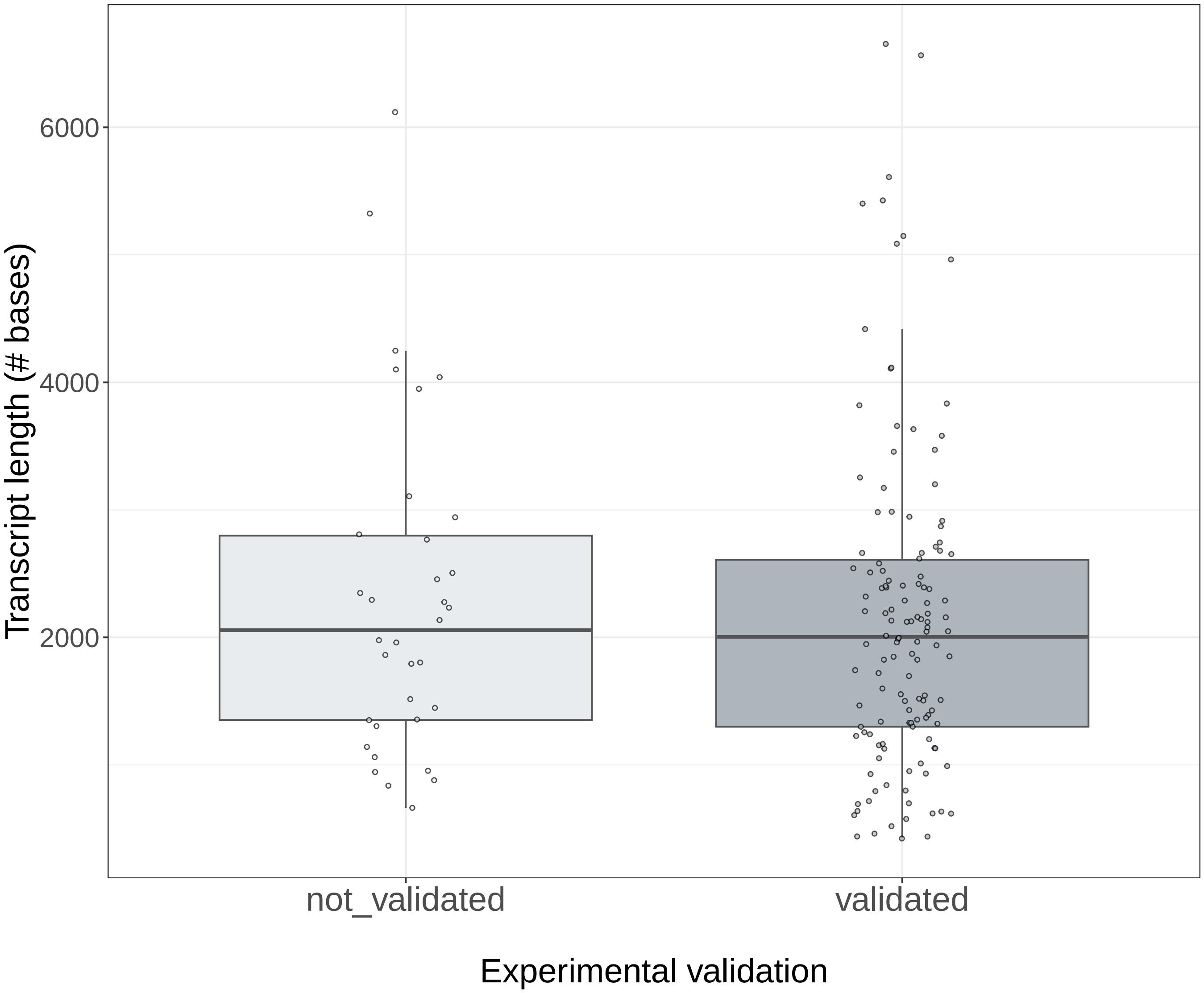

Supplementary Fig. 69. The distribution of lengths corresponding to the target transcript isoform across the entire validation experiment (including GENCODE, Platform, and Consistency groups), broken down by their validation status.

a

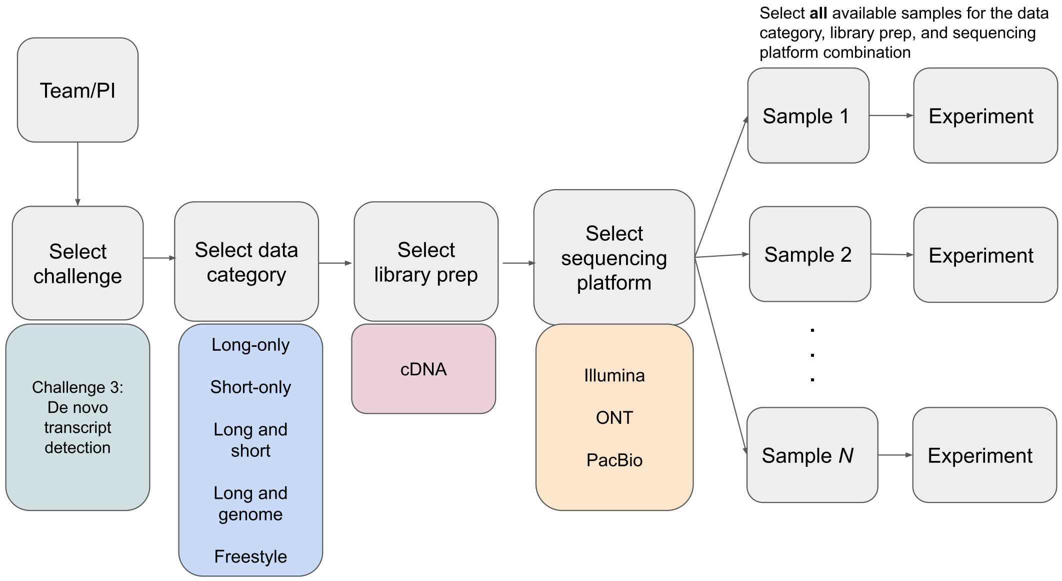

b

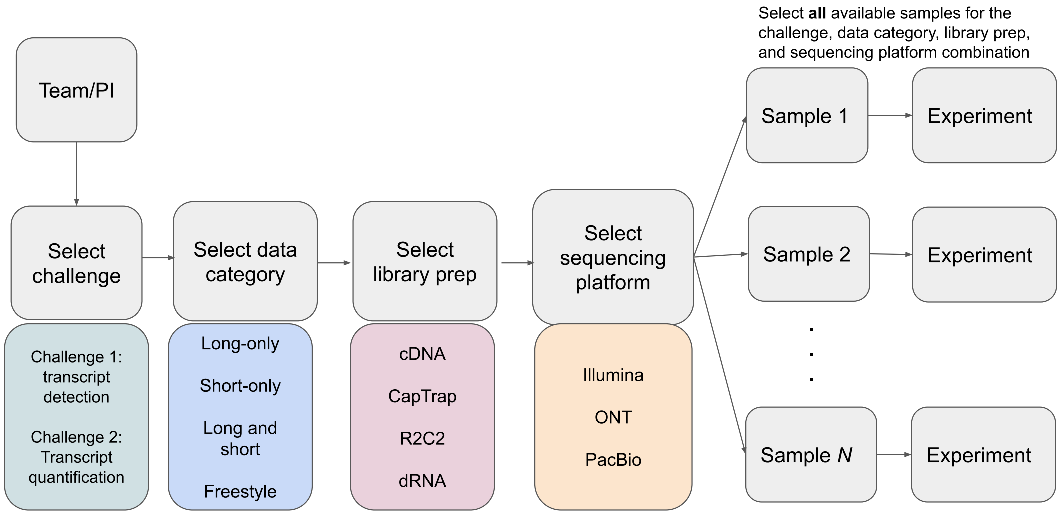

Supplementary Fig. 70. Challenge submission. a) Overview of submissions to Challenges 1 and 2. Each entry was derived from a specific data category, library prep, and sequencing platform combination. All available samples for the selected combination must be included in an entry. b) Overview of submissions for Challenge 3.

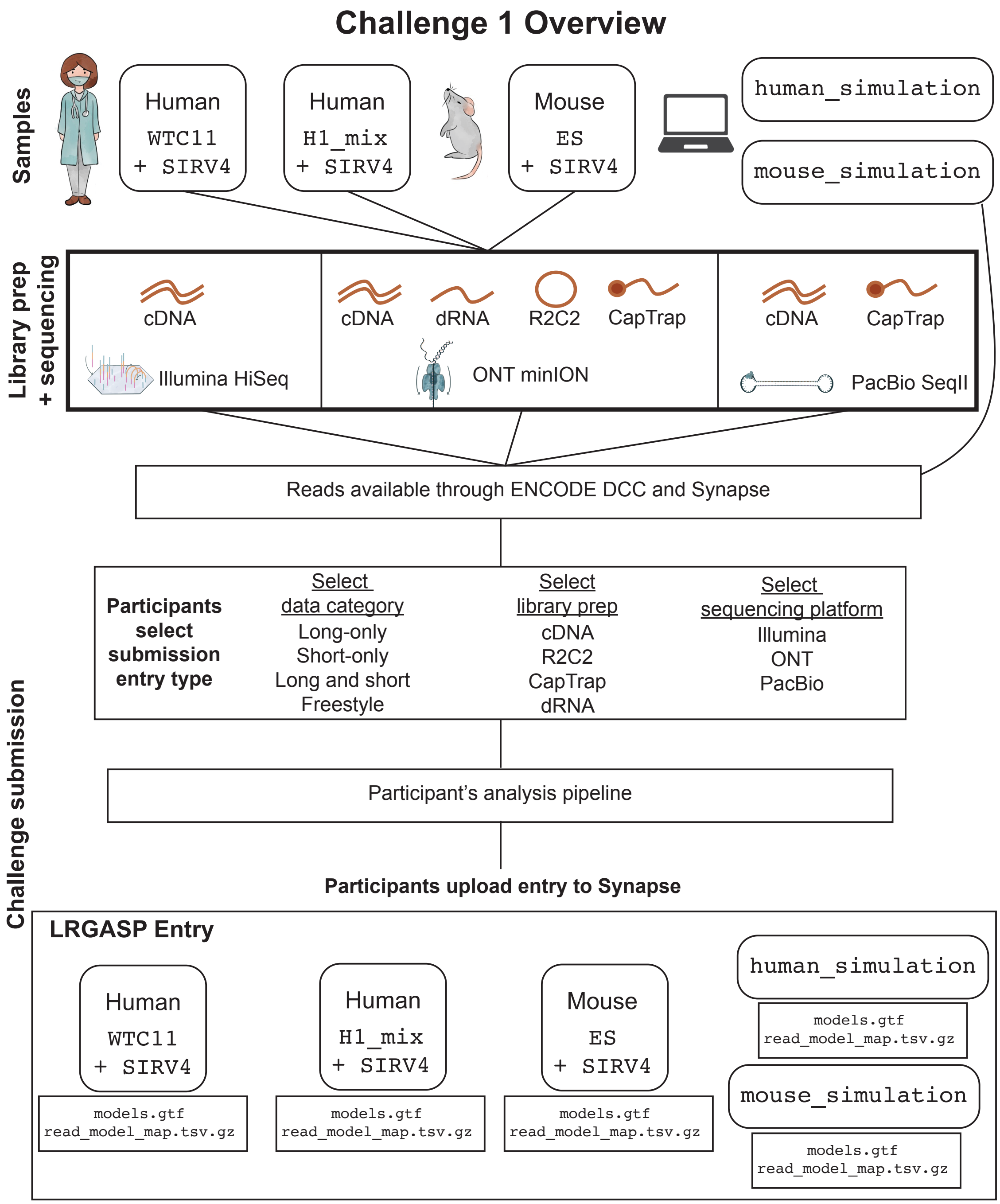

Supplementary Fig. 71. Flow diagram of Challenge 1: Transcript isoform detection with a high-quality genome. Samples, library prep methods, and sequencing platforms used in the challenge are indicated at the top. Participants select which data category, library prep, and sequencing platform to analyze, run their pipelines to generate transcript predictions, and submit an entry which includes predictions for all samples. The entries include a GTF file of the transcript models and a TSV file that assigns reads that supported each transcript model.

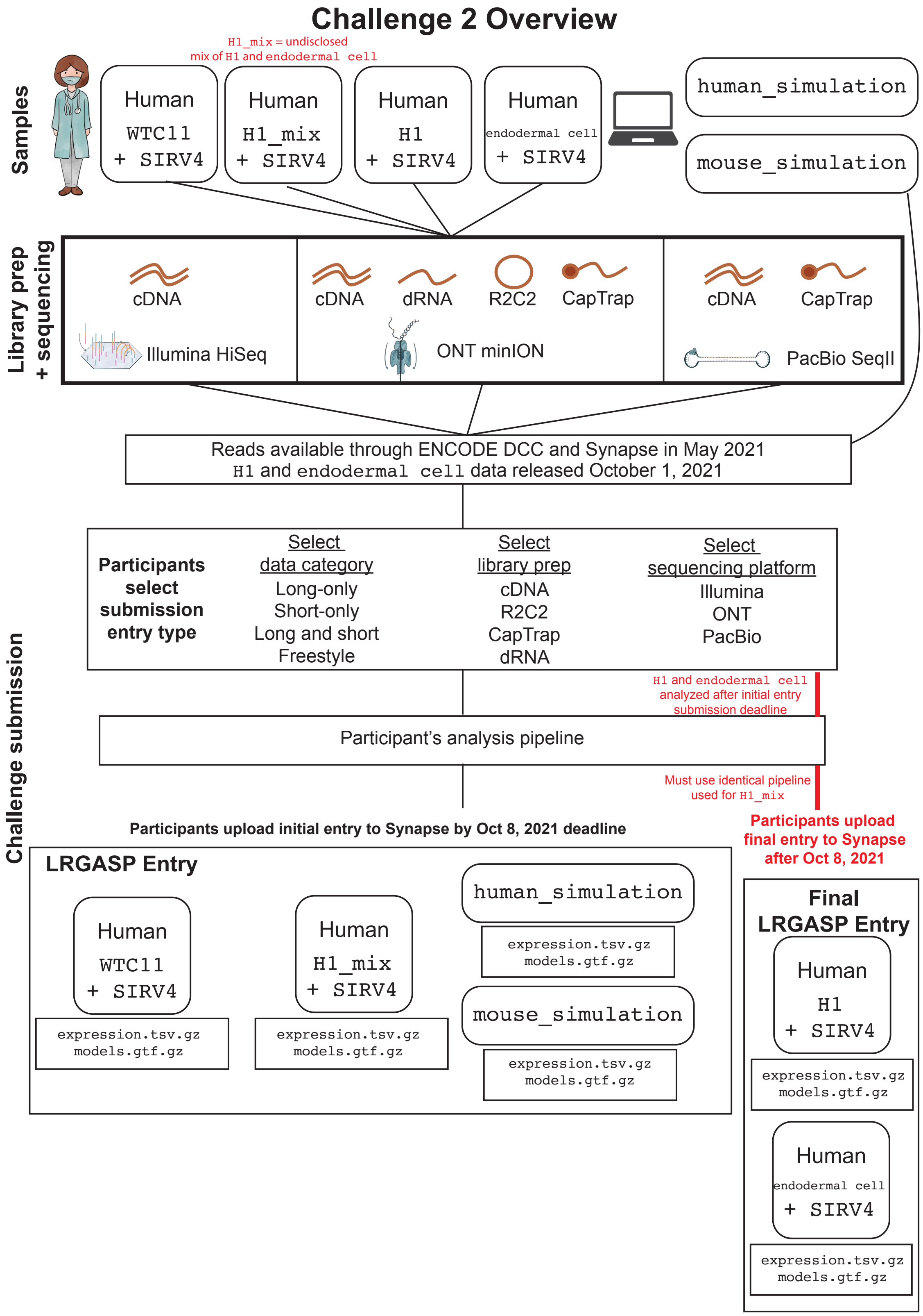

Supplementary Fig. 72. Flow diagram of Challenge 2: Transcript isoform quantification. Samples, library prep methods, and sequencing platforms used in the challenge are indicated at the top. Participants select which data category, library prep, and sequencing platform to analyze, run their pipelines to generate transcript predictions, and submit an entry which includes predictions for all samples. The entries include a GTF file of the transcript models that are quantified and a TSV file of the expression quantification. The H1 and endodermal cell samples were released after the initial submission deadline and participants were required to submit the quantification after the deadline.

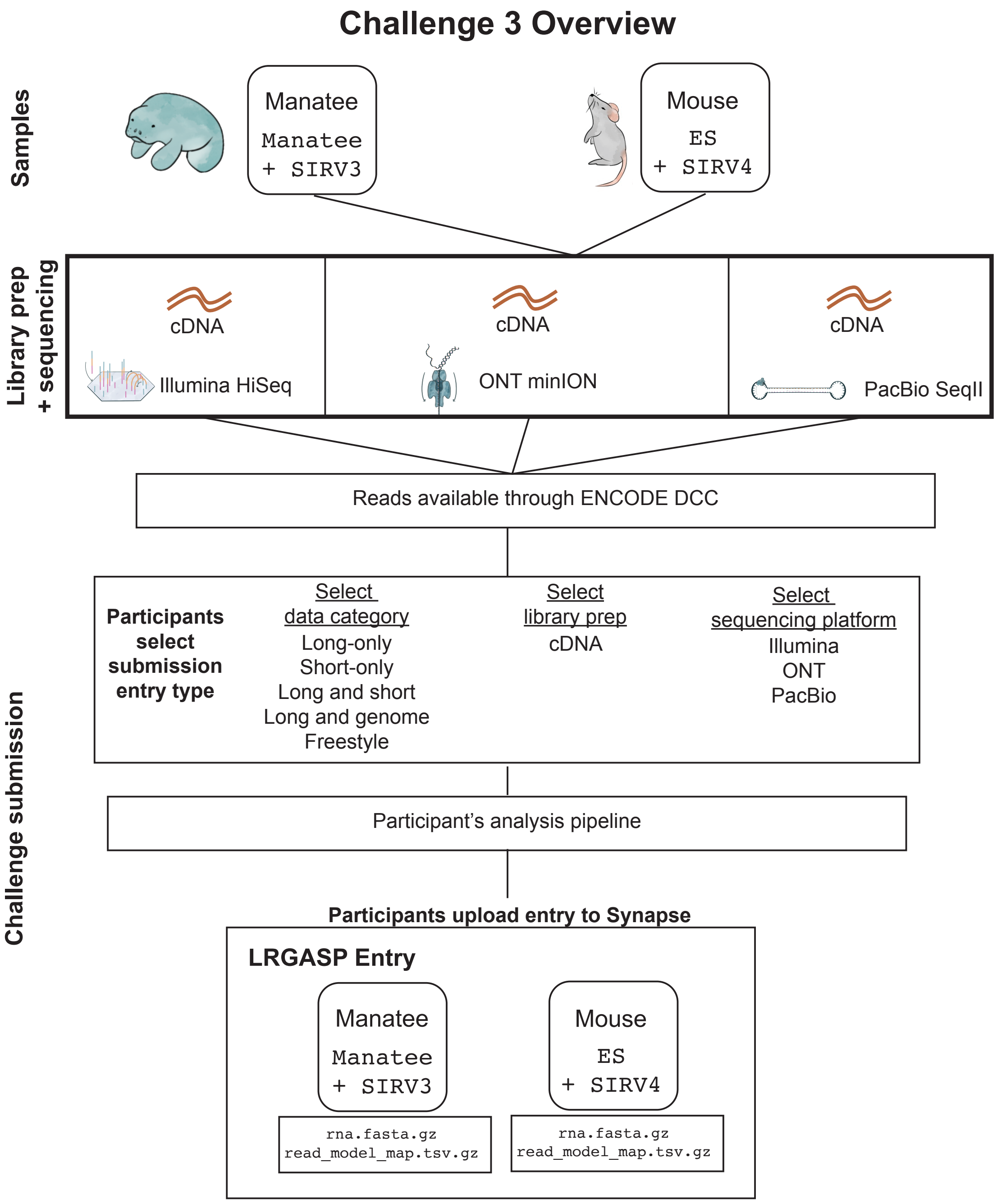

Supplementary Fig. 73. Flow diagram of Challenge 3. Samples, library prep methods, and sequencing platforms used in the challenge are indicated at the top. Participants select which data category and sequencing platform to analyze, run their pipelines to generate transcript predictions, and submit an entry which includes predictions for all samples. The entries include a FASTA file of the transcript models and a TSV file that assigns reads that supported each transcript model.

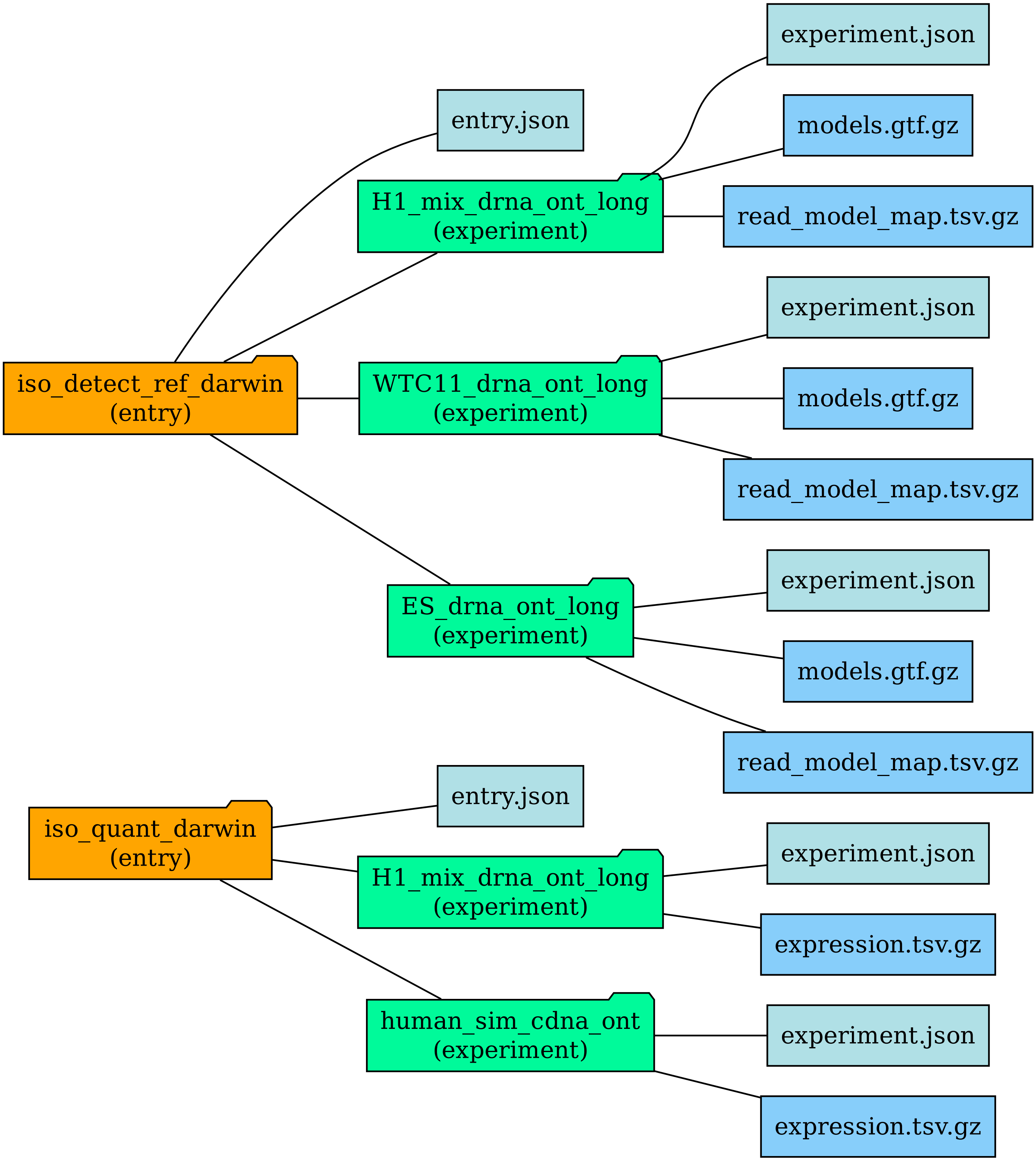

Supplementary Fig. 74. Schematic of directory structure and files that are included in each entry.

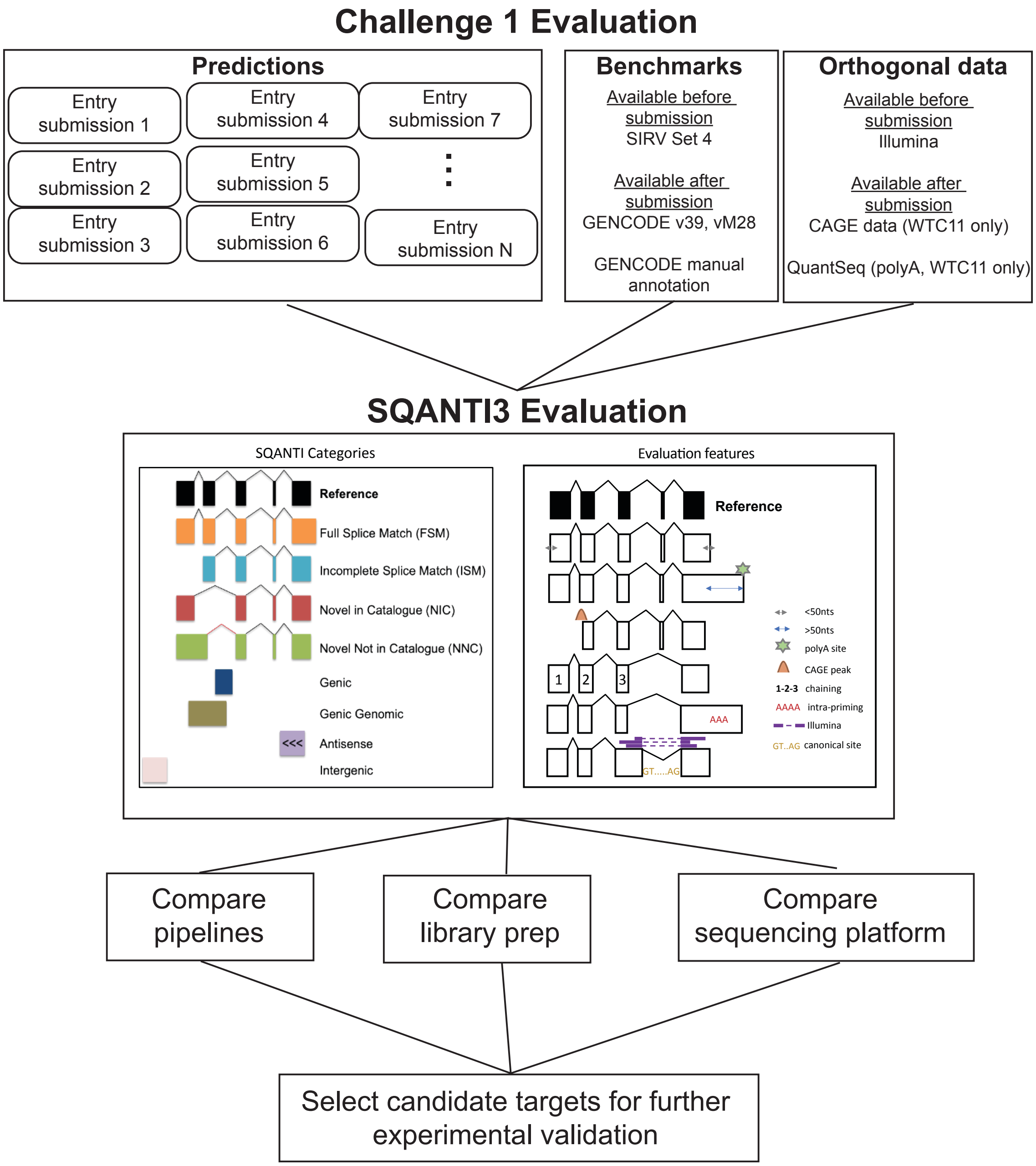

Supplementary Fig. 75. Flow diagram of the evaluation for Challenge 1. Benchmarks and additional orthogonal data that was used for the evaluation are indicated. For example, CAGE and QuantSeq data from WTC11 cells were generated and made available only after participant submissions; therefore, they represent "hidden" data. These was used to define 5' transcript starts and 3' ends.

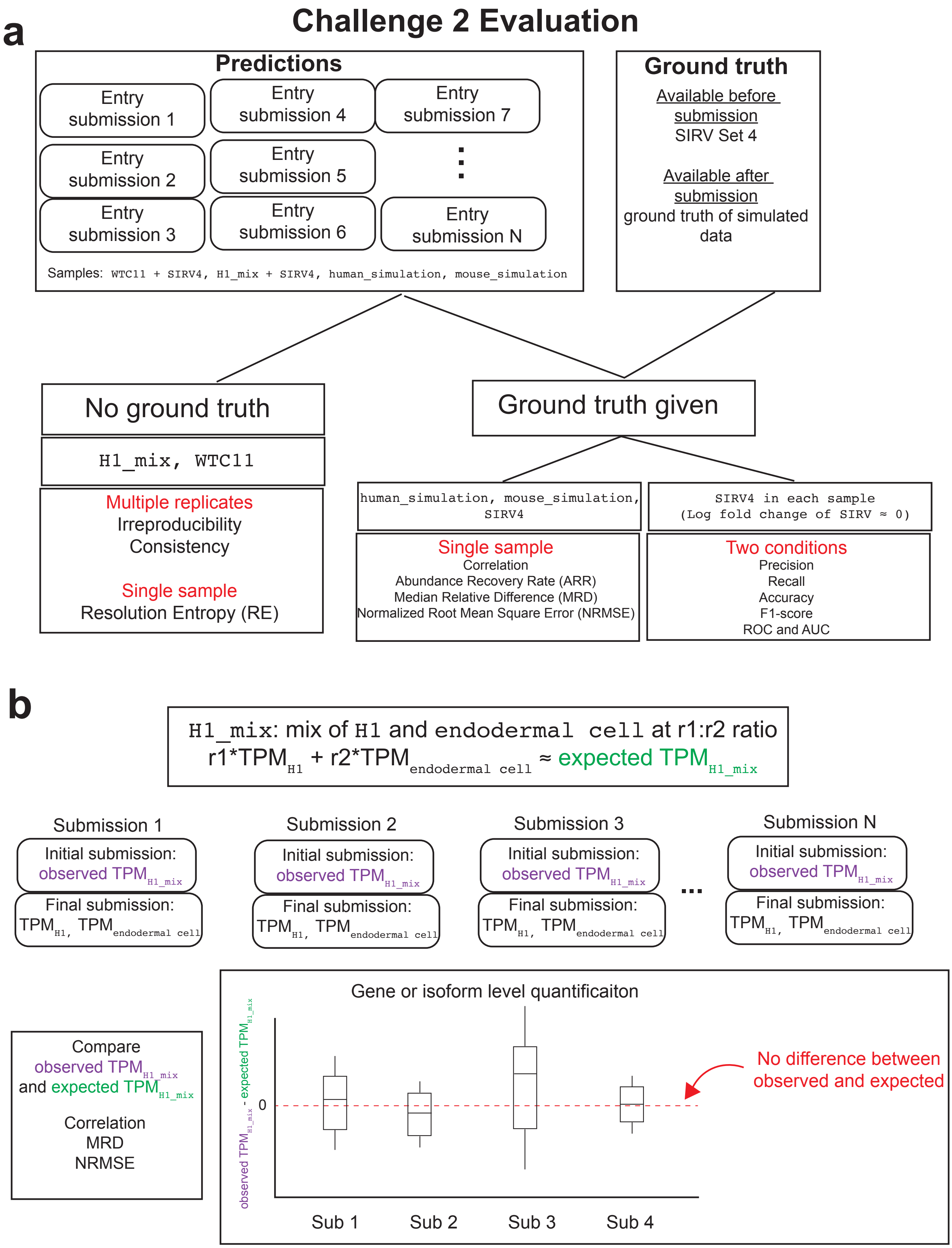

Supplementary Fig. 76. Flow diagram of the evaluation for Challenge 2. a) Evaluation of Challenge 2 can be separated into metrics when a ground truth is known or a ground truth is unknown. b) Example analyses to evaluate transcript expression using the cell mixing experiment. A sample, H1_mix, was initially provided for quantification which was a mix of H1 cells and endodermal cells at an undisclosed ratio. After the initial submission, the individual H1 and endodermal cell samples were released and participants submitted quantifications for each.

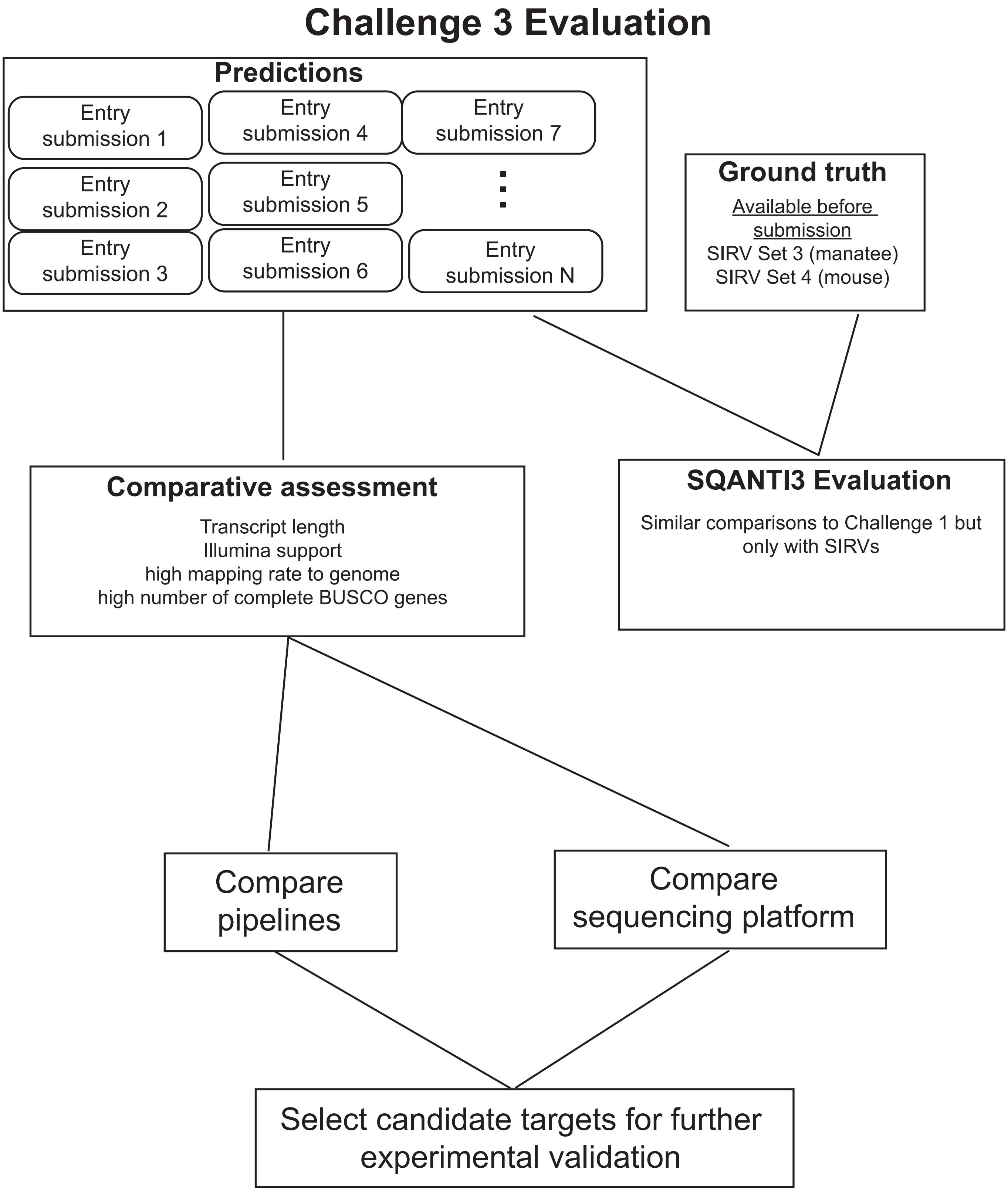

Supplementary Fig. 77. Flow diagram of the evaluation for Challenge 3. Only SIRVs are available for ground truth information. The evaluation was based on a comparative assessment of the predictions followed by targeting specific candidates for further validation.

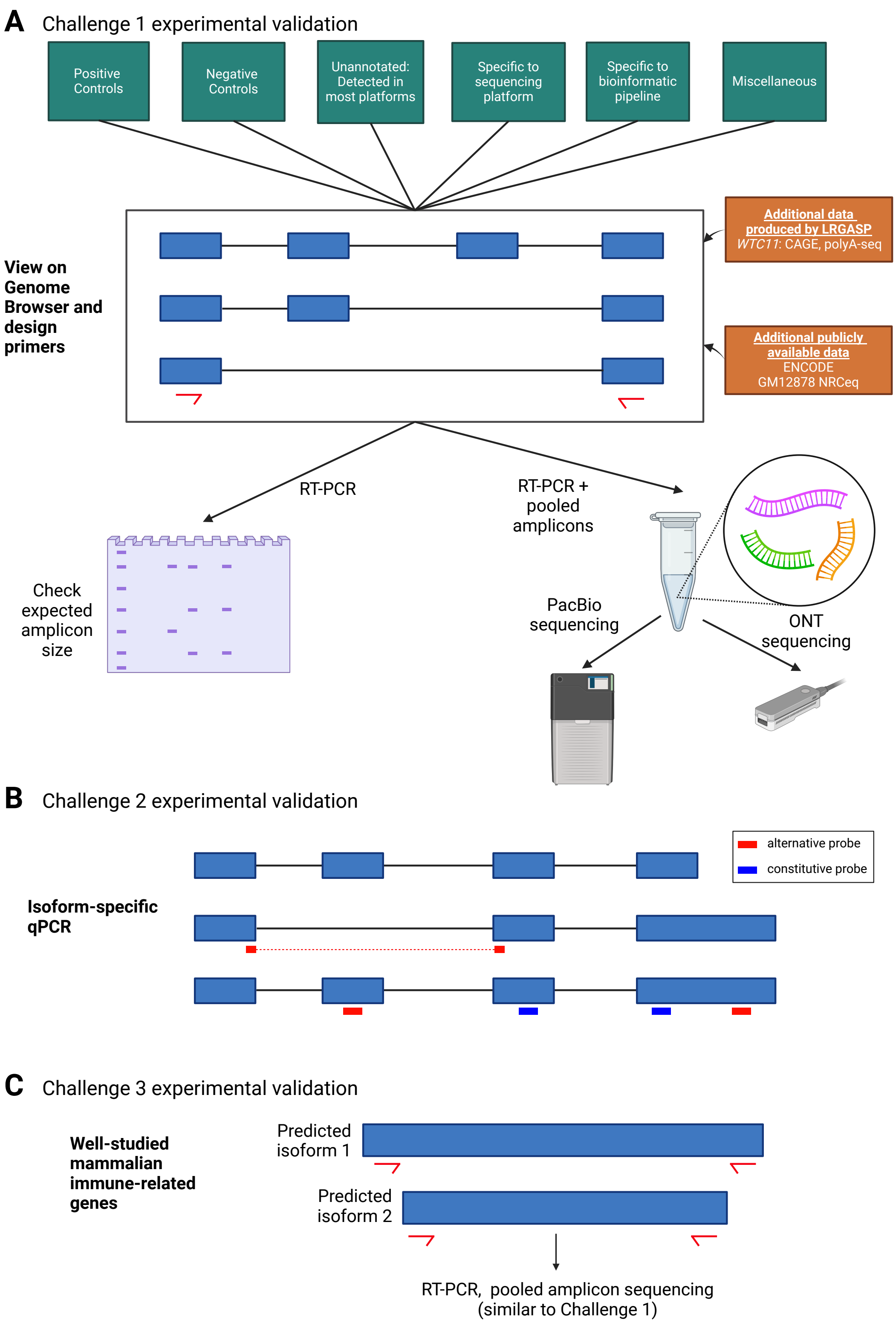

Supplementary Fig. 78. Experimental validation approaches for the LRGASP challenges. (A) Multiple categories of types of transcript were selected for validation (shown in green boxes). These loci will be viewed in the UCSC Genome Browser along with additional datasets to aid in the manual design of primers. Amplicons will be analyzed by fragment size and pooled to perform long-read sequencing with PacBio and ONT (B) A select number of genes were selected for transcript isoform-specific qPCR. A combination of probes detecting constitutive and alternative regions will be used. (C) RT-PCR validation will be performed similar to Challenge 1, except transcript were selected from well-studied mammalian immune-related genes.

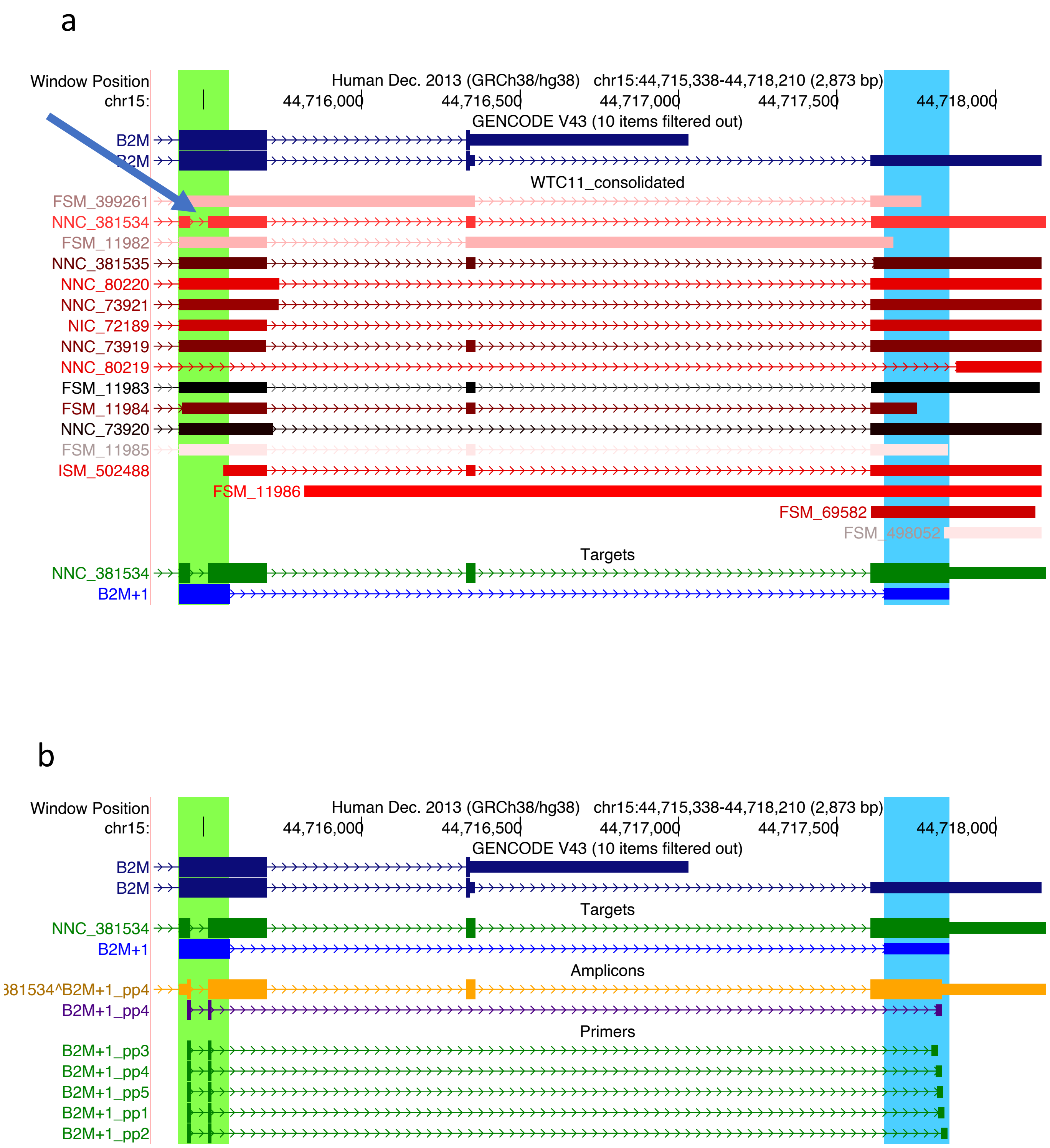

Supplementary Fig. 79. Designing validation primers. a) An example of a unique intron in transcript NNC_381534 to validation. The green and blue region vertical highlights indicate the manually selected primer pair regions. The 'Targets' track, produced by Primers-Juju, recapitulates the region as blue item B2M+1, and transcript with the maximal possible amplicon drawn in thick boxes. b) The Primers-Juju track hub with the addition of the primer pairs design. This adds Primer3 results (Primers track) and the most stable primer along with the amplicon sequence for the target transcript (Amplicons track).
